# Supplementary material for: Health improvement framework for actionable treatment planning using a surrogate Bayesian model
Source: Nat Commun. 2021 May 25;12:3088. doi: 10.1038/s41467-021-23319-1 (PMC8149666; doi:10.1038/s41467-021-23319-1)
Supplement: Supplementary file 1 — Supplementary Information [file 41467_2021_23319_MOESM1_ESM.pdf]

Supplementary Information

Health improvement framework for actionable treatment planning using a surrogate Bayesian model

Kazuki Nakamura[1,2], Ryosuke Kojima[2], Eiichiro Uchino[2], Koh Ono[3], Motoko Yanagita[4,5], Koichi Murashita[6], Ken Itoh[7], Shigeyuki Nakaji[8] and Yasushi Okuno[2*]

[1] Research & Business Development Department, Kyowa Hakko Bio Co., Ltd., Tokyo, 100-0004, Japan.

[2] Department of Biomedical Data Intelligence, Graduate School of Medicine, Kyoto University, Kyoto, 606-8507, Japan.

[3] Department of Cardiovascular Medicine, Graduate School of Medicine, Kyoto University, Kyoto, 606-8507, Japan.

[4] Department of Nephrology, Graduate School of Medicine, Kyoto University, Kyoto, 606-8507, Japan.

[5] Institute for the Advanced Study of Human Biology, Kyoto University, Kyoto, 606-8507, Japan.

[6] Center of Innovation Research Initiatives Organization, Hirosaki University, Zaifu-cho, Hirosaki, Aomori, 036-8562, Japan.

[7] Department of Stress Response Science, Hirosaki University Graduate School of Medicine, Zaifu-cho, Hirosaki, Aomori, 036-8562, Japan.

[8] Department of Social Health, Hirosaki University Graduate School of Medicine, Zaifu-cho, Hirosaki, Aomori, 036-8562, Japan.

[*] Address correspondence to Yasushi Okuno, Graduate School of Medicine, Kyoto University, Yoshida-Konoe-cho, Sakyo-ku, Kyoto, 606-8507, Japan, TEL: +81-75-751-4881, FAX: +81-75-751-4881, E-mail: okuno.yasushi.4c@kyoto-u.ac.jp

Supplementary Notes

Four supplementary experiments are described below. First, we conducted a more extensive evaluation of our framework with a five-dimensional (5D) synthetic dataset considering the real-life applications than the three-dimensional (3D) synthetic dataset. Second, details of the clinicians' evaluations of the improvement paths planned using our framework are described. Third, applications on the hypertension risk classification task and estimated glomerular filtration rate (eGFR) regression task using Iwaki Health Promotion Project (IHPP) dataset are described. Fourth, we applied our framework on a non-health dataset and another simple health dataset because the prediction model based on the health dataset used in the main text had low-prediction scores.

Validation of framework on 5D synthetic dataset

We generated a 5D dataset to verify whether our framework can plan paths by transiting the nodes with high probabilities in the variable space. This synthetic dataset was generated from five, 5D normal distributions (Supplementary Fig. 42). Assuming the correlation between explanatory variables, covariance was randomly set. Each distribution generated 200 data points that consisted of x_i $(i \in \{1, \dots, 5\})$. The response variables were set to the sum of x_i with Gaussian noise (σ = 2). The dataset consisted of 1,000 data points and randomly split into training data (80%) and test data (20%).

We built regression models based on XGBoost, random forest (RF), and support vector machine (SVM) (Supplementary Fig. 12a–c). Subsequently, stochastic surrogate models using hierarchical Bayesian modeling were constructed (Supplementary Fig. 12d–f). Using the stochastic surrogate models with lowest widely applicable Bayesian information criterion (WBIC), we planned paths to decrease the value of the response variable. All explanatory variables were selected for intervention variables, and the unit cell size of the grid was set to 0.5 σ in the training data for each explanatory variable. The path search algorithm was executed with L = 20,000 for each instance, and an optimal path to the node with the lowest predictive value was acquired. Planned paths were more actionable than the baseline paths (Supplementary Fig. 12g–i). The planned paths of two randomly selected instances for each prediction model are shown in Supplementary Figs. 13–15. We demonstrated that our framework applied to the dataset with covariance and could be used to plan paths with five intervention variables.

Details of utility assessments of health improvement paths planned by framework

The clinician assessments were performed on the utility of the framework related to the systolic blood pressure (SBP) regression task and chronic kidney disease (CKD) risk classification task as described in the main text. This section provides a discussion of suggestive instances in clinicians' evaluation.

Regarding instance 11 in the SBP regression task, the clinician evaluated that while lowering blood glucose might be reasonable in lowering blood pressure, the destination value of blood glucose was too low, which could pose other risks associated with hypoglycemia (Supplementary Fig. 43a–c). In this study, the search range, i.e., upper and lower limit, for the intervention variables were not defined to perform the objective path planning in the path search. While this can lead to more effective blood pressure-improving effects with interventions that clinicians do not usually take, it can also have side effects on the patient's health. Therefore, for example, regarding the blood glucose level, it would be better to avoid undesirable intervention by setting the lower limit based on the guideline or clinician's knowledge in practical application. Regarding instance 12, the clinicians disagreed with the framework-proposed path in which blood glucose and gamma glutamyl transferase (γ-GTP) were intervened instead of clearly high body mass index (BMI) (Supplementary Fig. 43d–f). As is shown in the heatmap, there were few instances where the BMI exceeds 30 in the dataset, which might cause overfitting on the original nonlinear prediction model construction. For example, if there are accidentally a few instances with obesity but low blood pressure, the prediction model may present predictive blood pressure lower than the actual regarding obesity instances. Since our framework presents the optimal path to the node with the most improved response variable value in the search iteration count based on the predictive model, the prediction model has a major impact on the destination node selection. Although we used health checkup data for a specific region, it would be desired to build a robust predictive model using a larger sample size dataset.

Regarding instance 13 in the CKD risk classification task, the framework-proposed path was composed of the alternating interventions in high triglyceride and right ankle diastolic blood pressure (RADIA), which is blood pressure-related indicators (Supplementary Fig. 44a–c). Also, the path was consistent with the distribution of actual data for these variables. From these results, the clinicians

evaluated that this health-improvement path was plausible and could lead to behavioral changes in the patient. In this study, we used a grid graph for the path search algorithm, and only one variable is intervened at a time. Paths would be planned by repeating interventions, and simultaneous fluctuations of multiple variables could be represented by alternating fluctuations. Regarding instance 14, the clinicians evaluated that intervention on overweight and blood pressure would be necessary though the framework-proposed path consisted of intervention in triglyceride and hemoglobin (Hb) (Supplementary Fig. 44d–f). While the path planned to improve the response variable without specifying target values of intervention variables might present new possibilities, it could be better in some cases that the clinician set target values of intervention variables. By changing the search end condition in the path search algorithm, our framework can present the optimal path to the node of the targeted-intervention variable values determined by the clinicians.

Application of framework on hypertension risk classification task using IHPP dataset

We applied our framework to the hypertension risk classification task. According to the guideline for hypertension, the SBP cutoff value for the classification task was set to 140 mmHg[1]. As in the main text, we performed explanatory variable reduction using recursive feature elimination (RFE). Important features selected by XGBoost-based RFE comprised items related to hypertension, such as age, body composition (leg score and BMI), blood glucose, and γ-GTP[2–10] (Supplementary Fig. 22a). Following our framework, classification models were built after the replacement of missing values with the use of multiple imputations. Area under the curve (AUC) of the models are summarized in Supplementary Table 6. Next, we constructed stochastic surrogate models based on multiple imputed RFE-selected features and predicted values (Supplementary Fig. 22b–d). Though we initially set the range of mixture components to 1–8, the lowest WBIC values were obtained when the numbers of mixture components were eight in XGBoost and RF. Therefore, we expanded the range to 16 in these cases. Subsequently, we performed path planning using the stochastic surrogate models selected based on WBIC. The top-five variables that could be intervened were selected as intervention variables based on feature importance in a data-driven manner: γ-GTP, blood glucose, aspartate transaminase (AST [GOT]), leg score, and immunoglobulin G (IgG). Assuming a scenario wherein the task is to improve participants with higher SBP, relevant instances were selected according to the following

criteria: XGBoost-predictive class of SBP ≥140 mmHg[1], and no missing values in the intervention variables. The number of applicable instances was 162. We executed the path-search algorithm with L = 20,000 for each instance and acquired a path to the node with the lowest predictive SBP value in count L. The histograms of the actionability score are shown in Supplementary Fig. 22e–g. The paths of the two randomly selected instances are shown in Supplementary Fig. 23–25.

Application of framework on eGFR regression task using IHPP dataset

We applied our framework to the eGFR regression task. As in the main text, we performed explanatory variable reduction using RFE. Important features selected by RFE comprised items related to renal function, such as age, uric acid, blood urea nitrogen (BUN), anemia-related factors (Hb and erythrocyte count), and body composition-related factors (right arm X 50 kHz and basal metabolic rate [BMR] score)[11–15] (Supplementary Fig. 26a). Following our framework, regression models were built after the replacement of missing values with the use of multiple imputations. The scores of the prediction models are shown in Supplementary Fig. 26b–d. Next, we constructed stochastic surrogate models based on hierarchical Bayesian modeling based on multiple imputed RFE-selected features and predicted values (Supplementary Fig. 26e–g). Subsequently, we performed path planning using the stochastic surrogate models selected based on WBIC. The top-five variables that could be intervened were selected as intervention variables based on feature importance in a data-driven manner: uric acid, BUN, right arm X 50 kHz, BMR score, and erythrocyte count. Assuming a scenario wherein the task is to intervene the participants with decreased renal functions, relevant instances were selected according to the following criteria: XGBoost-predictive eGFR <60 mL/min/1.73 m^2 [16], and no missing values in the intervention variables. The number of applicable instances was 14. We executed the path-search algorithm with L = 20,000 for each instance and acquired a path to the node with the highest predictive eGFR value in count L. The histograms of the actionability score are shown in Supplementary Fig. 26h–j. The paths of the two randomly selected instances are shown in Supplementary Figs. 27–29.

Evaluation on real estate dataset

We obtained a dataset from the UCI Machine Learning Repository[17,18]. This dataset was used to conduct a regression analysis of the house price of a unit area based on six continuous explanatory variables of real estate data. There were no missing values in the dataset. The dataset was randomly split into training data (80%) and test data (20%).

The trained XGBoost regression model yielded a root-mean-square error (RMSE) of 6.78 and an R-squared value of 0.735 in the test data (Supplementary Fig. 45a). A surrogate model was constructed by hierarchical Bayesian modeling using the original data and the predicted values of the regression model. The lowest WBIC value was obtained when the number of mixture components was two (Supplementary Fig. 45b).

Subsequently, path planning was performed using the surrogate model. Among the six explanatory variables, X1 (the transaction date) was a variable that was difficult to intervene. Therefore, X1 was fixed, and the remaining five variables were selected as intervention variables. The unit cell size of the grid was set to 0.2 σ in the training data for each explanatory variable. We executed the path search algorithm with L = 20,000 for each instance, and the path with the highest predicted value was acquired. The histogram of the actionability score for each instance is shown in Supplementary Fig. 45c. The actionability scores were greater than zero in 75/82 instances, and the median was 5.25.

From these results, we have demonstrated that our framework can be applicable to datasets with higher regression model scores.

Evaluation on public dataset for disease progression

To evaluate the feasibility of our framework on another small health dataset, we used a public dataset on diabetes progression[19,20]. This dataset was used to conduct a regression of the quantitative measure of diabetes progression one year after the baseline from nine continuous and one discrete explanatory variable (Supplementary Table 7). The dataset is openly available on Trevor Hastie's Software page at https://web.stanford.edu/~hastie/Papers/LARS/. This dataset contains no missing values. The dataset was randomly split into training data (80%) and test data (20%).

The feature importance of the trained model is shown in Supplementary Fig. 46a. The XGBoost regression model yielded an RMSE value of 62.19 and an R-squared value of 0.246 in the test data

(Supplementary Fig. 46b). A surrogate model was constructed by hierarchical Bayesian modeling using the original data and the predicted values of the regression model. The lowest WBIC value was obtained when the number of mixture components was two (Supplementary Fig. 46c).

Subsequently, path planning was performed using the surrogate model. Regarding the intervention variables, five variables were selected from the top of the feature importance of the regression model: body mass index (BMI), blood pressure, T-cells, high-density lipoproteins, and lamotrigine (Supplementary Fig. 46a). The remaining variables were fixed. The unit cell size of the grid was set to 0.2 σ in the training data for each explanatory variable. We executed the path search algorithm with $L = 20{,}000$ in each instance, and the path with the lowest predicted value was acquired. The histogram of the actionability score for each instance is shown in Supplementary Fig. 46d. The actionability scores were greater than zero in 83/87 instances, and the median was 2.06. Examples of the paths planned by using the proposed framework are shown in Supplementary Fig. 47. This experiment indicates the feasibility of using the proposed framework in planning actionable paths to improve the predictions of the regression model.

Supplementary References

1. Umemura, S. *et al.* The Japanese Society of Hypertension Guidelines for the Management of Hypertension (JSH 2019). *Hypertension Research* **42**, 1235–1481 (2019).
2. Kannel, W. Fifty years of Framingham Study contributions to understanding hypertension. *Journal of Human Hypertension* **14**, 83–90 (2000).
3. Rockwood, M. R. H. & Howlett, S. E. Blood pressure in relation to age and frailty. *Canadian Geriatrics Journal* **14**, 2–7 (2011).
4. Foulds, H. J. A., Bredin, S. S. D. & Warburton, D. E. R. The relationship between hypertension and obesity across different ethnicities. *Journal of Hypertension* **30**, 359–367 (2012).
5. Ye, S. *et al.* Associations of Body Composition with Blood Pressure and Hypertension. *Obesity* **26**, 1644–1650 (2018).
6. Wada, T., Kawasaki, Y. & Inaji, J. Establishing Borderline and At-risk Regions for Estimated Skeletal Muscle Mass of Legs Determined with a Body Composition Meter. *Ningen Dock International* **2**, 14–18 (2014).
7. Tatsumi, Y. *et al.* Fasting Blood Glucose Predicts Incidence of Hypertension Independent of HbA1c Levels and Insulin Resistance in Middle-Aged Japanese: The Saku Study. *American Journal of Hypertension* **32**, 1178–1185 (2019).
8. Kuwabara, M. *et al.* Fasting blood glucose is predictive of hypertension in a general Japanese population. *Journal of Hypertension* **37**, 167–174 (2019).
9. Shimizu, Y. *et al.* Gamma-glutamyl transpeptidase (γ-GTP) has an ambivalent association with hypertension and atherosclerosis among elderly Japanese men: A cross-sectional study. *Environmental Health and Preventive Medicine* **24**, 1–7 (2019).
10. Yamada, Y. *et al.* The Relationship between Serum Gamma-Glutamyl Transpeptidase Levels and Hypertension: Common in Drinkers and Nondrinkers. *Hypertension Research - Clinical and Experimental* **18**, 295–301 (1995).
11. Wan, C. S. *et al.* Bioelectrical impedance analysis to estimate body composition, and change in adiposity, in overweight and obese adolescents: Comparison with dual-energy x-ray absorptiometry. *BMC Pediatrics* **14**, 1–10 (2014).
12. Weinstein, J. R. & Anderson, S. The Aging Kidney: Physiological Changes. *Advances in Chronic Kidney Disease* **17**, 302–307 (2010).
13. Obermayr, R. P. *et al.* Elevated uric acid increases the risk for kidney disease. *Journal of the American Society of Nephrology* **19**, 2407–2413 (2008).
14. Rossert, J. *et al.* Effect of Early Correction of Anemia on the Progression of CKD. *American Journal of Kidney Diseases* **47**, 738–750 (2006).
15. Hall, J. *et al.* Obesity, hypertension, and chronic kidney disease. *International Journal of Nephrology and Renovascular Disease* 75 (2014).
16. Japanese Society of Nephrology. Evidence-based Clinical Practice Guideline for CKD 2013. *Clinical and Experimental Nephrology* **18**, 346–423 (2014).

17. Dua, D. & Graff, C. UCI Machine Learning Repository. http://archive.ics.uci.edu/ml (2019).
18. Yeh, I. C. & Hsu, T. K. Building real estate valuation models with comparative approach through case-based reasoning. *Applied Soft Computing Journal* **65**, 260–271 (2018).
19. Tibshirani, R., Johnstone, I., Hastie, T. & Efron, B. Least angle regression. *The Annals of Statistics* **32**, 407–499 (2004).
20. Hastie, T. & Efron, B. lars: Least Angle Regression, Lasso and Forward Stagewise. *R package version 1.2* (2013).
21. Matsuo, S. *et al.* Revised Equations for Estimated GFR From Serum Creatinine in Japan. *American Journal of Kidney Diseases* **53**, 982–992 (2009).
22. Leib, E. S., Lewiecki, E. M., Binkley, N. & Hamdy, R. C. Official Positions of the International Society for Clinical Densitometry. *Journal of Clinical Densitometry* **7**, 1–5 (2004).
23. Hye, S. C., Cheon, W. C., Myung, J. P., Hong, M. K. & Yoo, J. H. Clinical value of a desktop spirometer (HI-801) for spirometry screening. *Tuberculosis and Respiratory Diseases* **62**, 276–283 (2007).
24. Kubota, M. *et al.* Reference values for spirometry, including vital capacity, in Japanese adults calculated with the LMS method and compared with previous values. *Respiratory Investigation* **52**, 242–250 (2014).
25. Momma, H. *et al.* Physical fitness tests and type 2 diabetes among Japanese: A longitudinal study from the niigata wellness study. *Journal of Epidemiology* **29**, 139–146 (2019).

Supplementary Figures

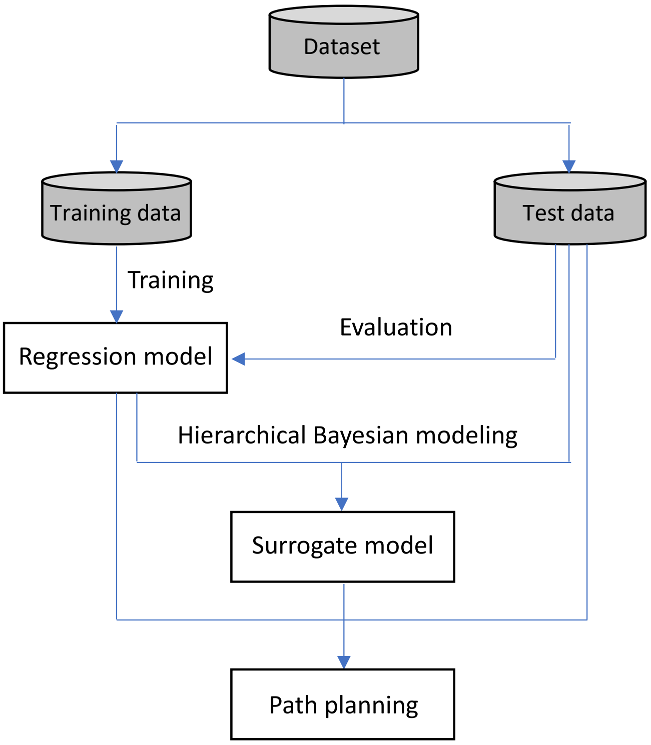

Supplementary Figure 1. Detailed workflow of proposed framework.

$$\begin{bmatrix} x_1 \\ x_2 \\ x_3 \end{bmatrix} \sim \mathcal{N}(\boldsymbol{\mu}_k, \boldsymbol{\Sigma}_k) \quad k \in \{1, 2, 3\}$$

$$\boldsymbol{\mu}_1 = \begin{bmatrix} 0 \\ -5 \\ -5 \end{bmatrix} \quad \boldsymbol{\Sigma}_1 = \begin{bmatrix} 5 & 0 & 0 \\ 0 & 1 & 0 \\ 0 & 0 & 1 \end{bmatrix}$$

$$\boldsymbol{\mu}_2 = \begin{bmatrix} 5 \\ 0 \\ -5 \end{bmatrix} \quad \boldsymbol{\Sigma}_2 = \begin{bmatrix} 1 & 0 & 0 \\ 0 & 5 & 0 \\ 0 & 0 & 1 \end{bmatrix}$$

$$\boldsymbol{\mu}_3 = \begin{bmatrix} 5 \\ 5 \\ 0 \end{bmatrix} \quad \boldsymbol{\Sigma}_3 = \begin{bmatrix} 1 & 0 & 0 \\ 0 & 1 & 0 \\ 0 & 0 & 5 \end{bmatrix}$$

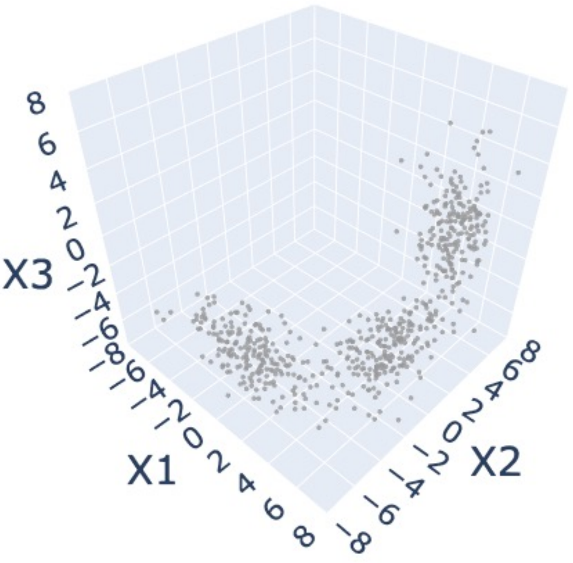

Supplementary Figure 2. Generation of three-dimensional (3D) synthetic dataset. The three 3D normal distributions generated 200 data points that consisted of x_1, x_2, and x_3. Subsequently, a response variable was set to the sum of x_1, x_2, and x_3 with Gaussian noise ($\sigma = 2$). The synthetic dataset consisted of a total of 600 data points with explanatory variables (X1, X2, and X3) and a response variable.

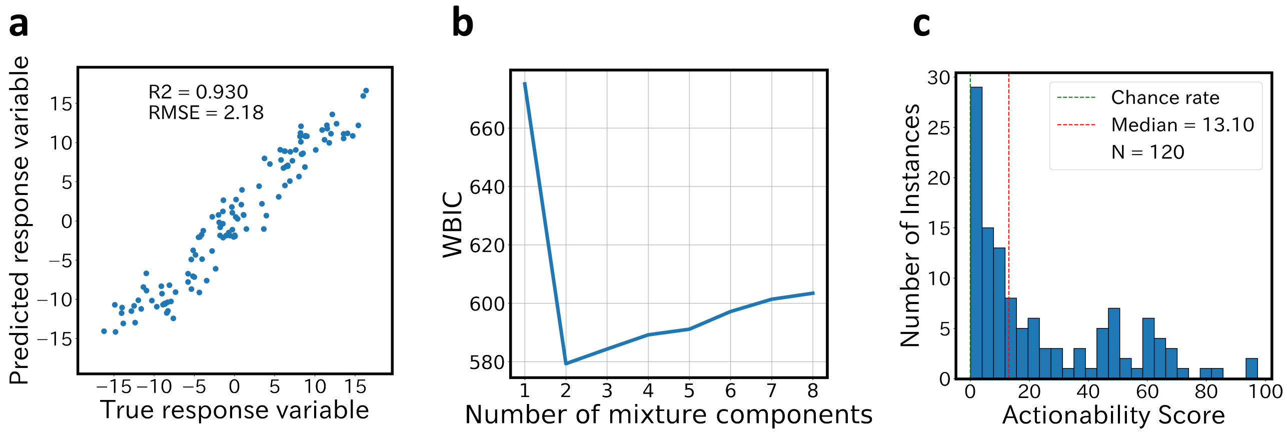

Supplementary Figure 3. Results of the proposed framework on three-dimensional (3D) synthetic dataset. a Plot for prediction vs. true response variable. **b** Widely applicable Bayesian information criterion (WBIC) values of the stochastic surrogate models with 1–8 mixture components. **c** Histogram of actionability scores at different instances. The unit cell size of the grid was set to 0.5 σ in the training data for each explanatory variable. The path search algorithm was executed with L = 20,000 for each instance and acquired a path with the lowest predictive value. An actionability score of zero indicates that the actionability of the optimal path is equivalent to that of the baseline path.

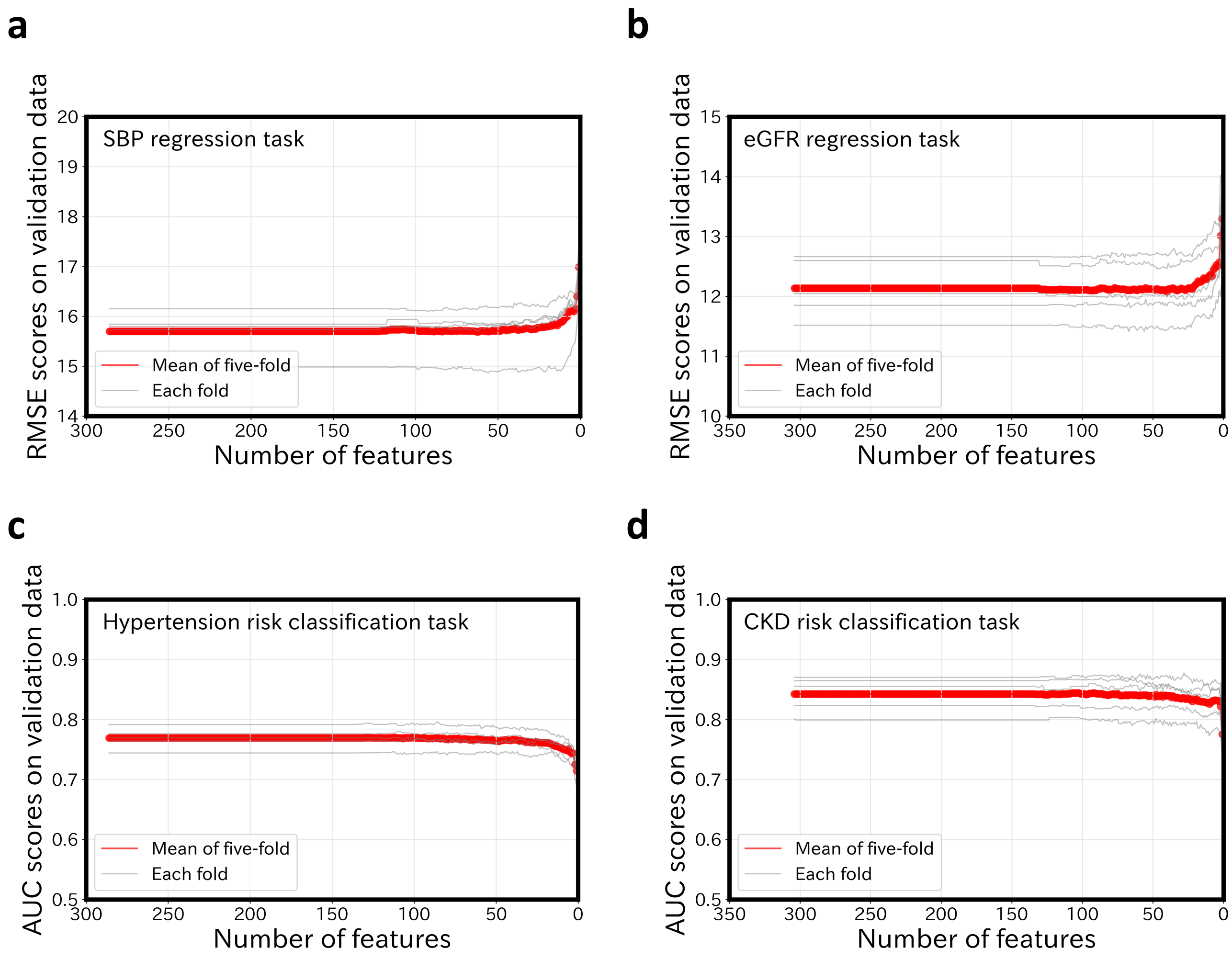

Supplementary Figure 4. XGBoost model scores during recursive feature elimination (RFE). Features are gradually reduced in RFE, and the scores on the validation data at each stage are shown. **a** Root-mean-squared error (RMSE) scores on systolic blood pressure (SBP) regression. **b** RMSE scores on estimated glomerular filtration rate (eGFR) regression. **c** Area under the curve (AUC) scores on hypertension risk classification. **d** AUC scores on CKD risk classification.

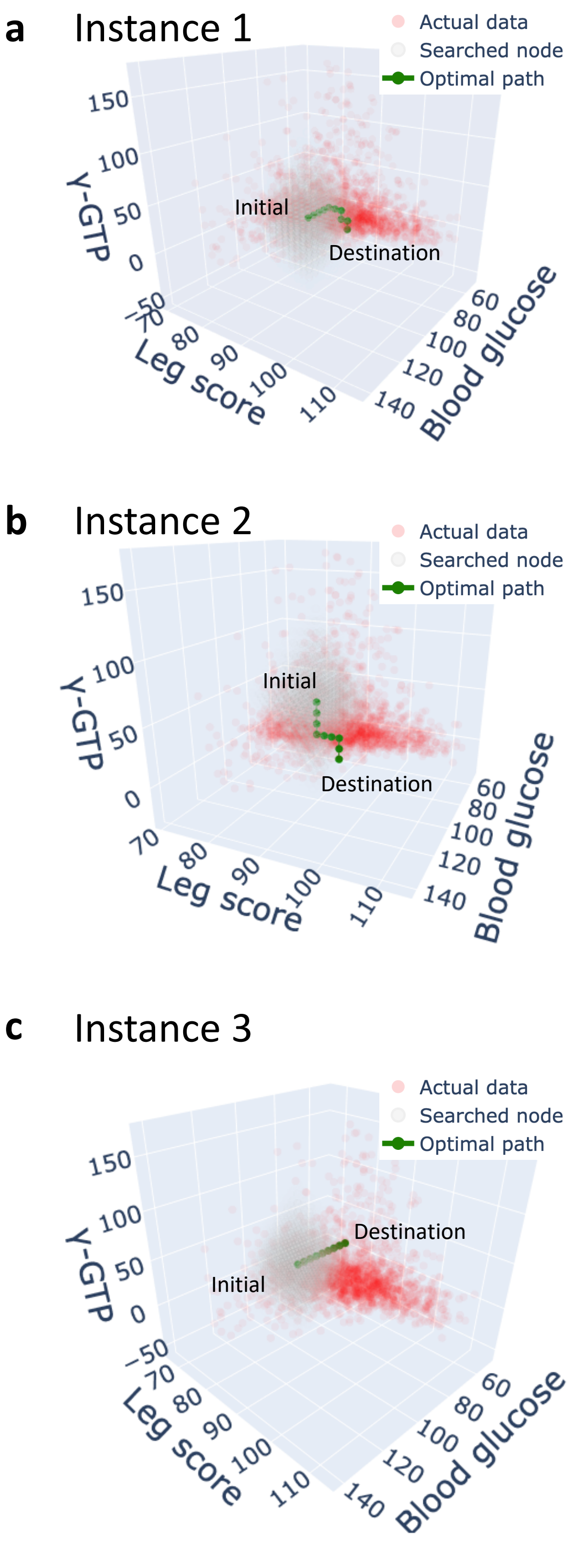

Supplementary Figure 5. 3D plots of optimal paths with intervention variables based on data-driven selection in systolic blood pressure (SBP) regression task. The instances respectively correspond to the instances in Fig. 6 of the main text.

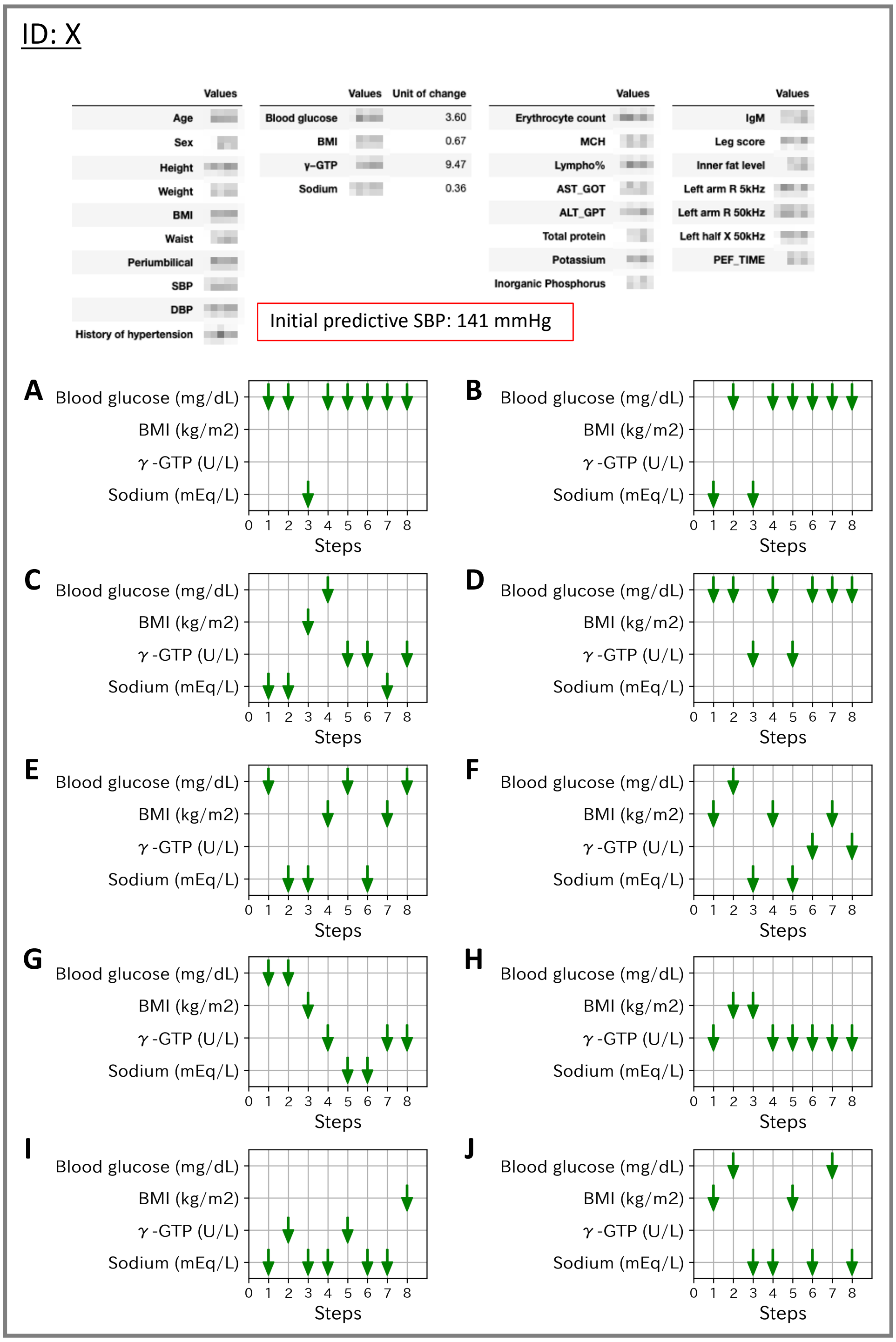

Supplementary Figure 6. Example question sheet for comparison with clinician intervention. An example of an evaluation sheet for the SBP regression task is provided above. The order of interventions and the initial values of measurement items of the instance (masked in this paper to protect personal health information) were presented to clinicians. Clinicians were asked to select an improvement path considered to be the closest to their treatment policy among ten blinded improvement paths (A–J), which comprised of a path planned by our framework and nine random paths. In the transition step, the up or down arrow represents an increase or decrease in the unit change of the intervention variable, respectively. Each clinician evaluated the same ten instances.

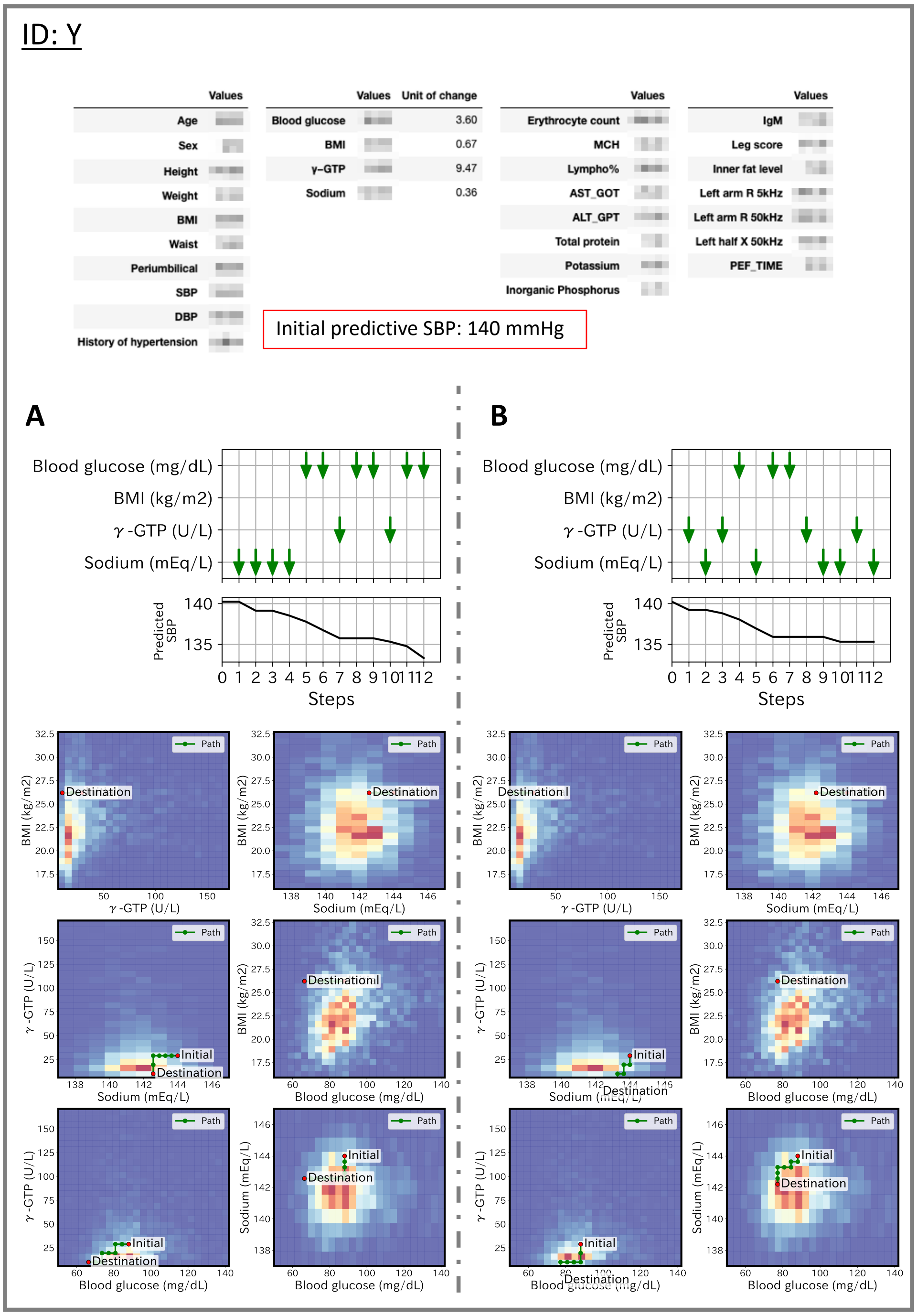

Supplementary Figure 7. Example question sheet for utility assessment. An example of an evaluation sheet for the SBP regression task is provided above. The clinicians were provided with the initial values of measurement items of the instance (masked in this paper to protect personal health information), as well as two blinded paths (A and B). The blinded paths are a path planned by our framework and a random path. For each path, the order of interventions along with the accompanied transition of the predictive response variable and the projection on 2D heatmaps for each pair of intervention variables were presented. In the transition step, the up or down arrow represents an increase or decrease in the unit change of the intervention variable, respectively. Clinicians evaluated the practicality and informativeness of each path with yes/no. Clinicians also commented on the reasons for choosing each evaluation. Each clinician evaluated the same ten instances.

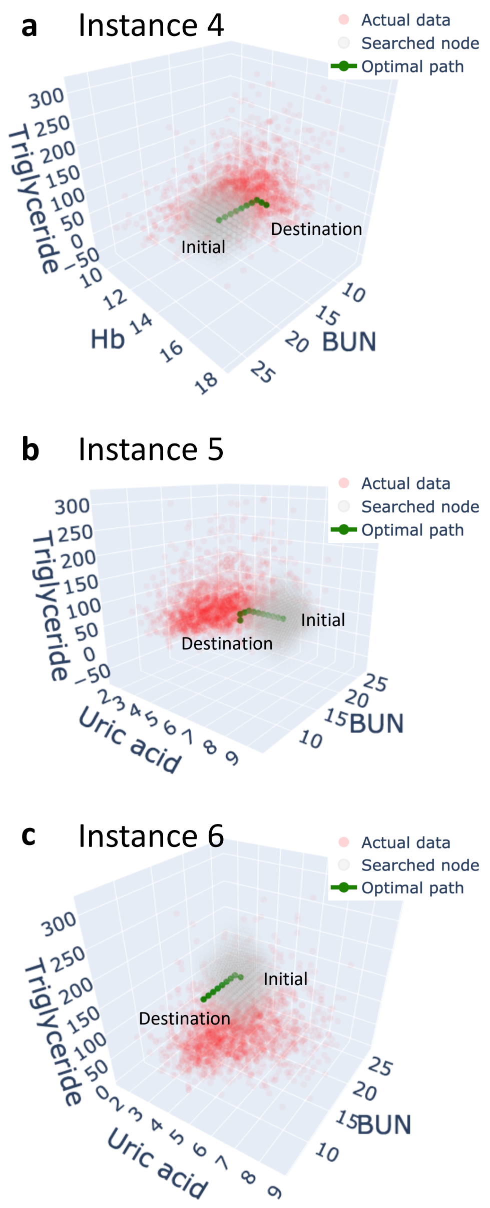

Supplementary Figure 8. 3D plots of optimal paths with intervention variables based on data-driven selection in chronic kidney disease (CKD) risk classification task. The instances respectively correspond to the instances in Fig. 8 of the main text.

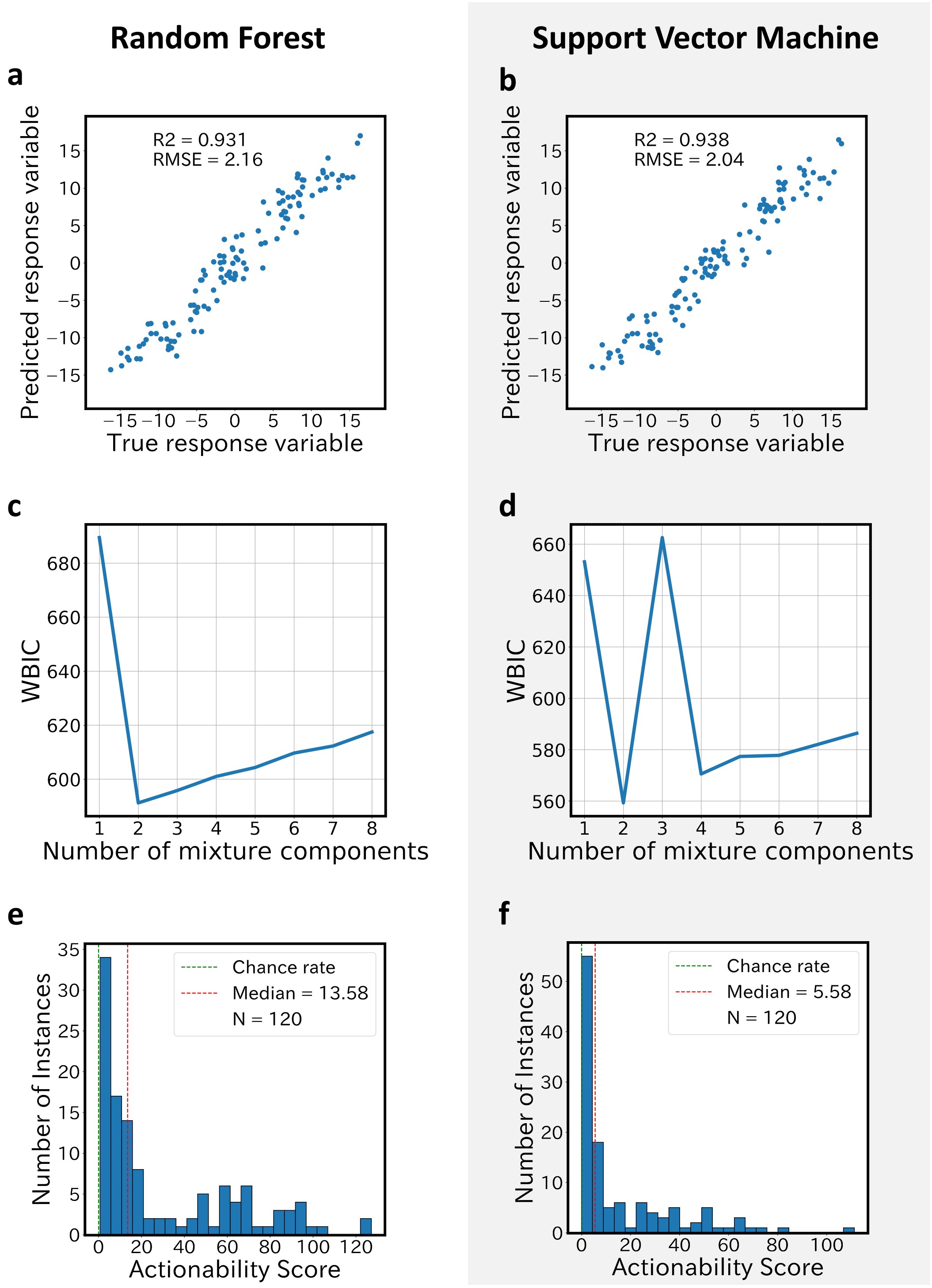

Supplementary Figure 9. Results of the proposed framework on three-dimensional (3D) synthetic dataset using different machine learning (ML) algorithms. The results of applying our framework to prediction models based on nonlinear ML algorithms other than XGBoost are shown: random forest (**a, c, e**) and support vector machine (**b, d, f**). **a, b** Plots for prediction vs. true response variable. **c, d** Widely applicable Bayesian information criterion (WBIC) values of stochastic surrogate models with 1–8 mixture components. **e, f** Histogram of actionability scores with intervention variables based on data-driven selection at different instances. An actionability score of zero indicates that the actionability of the optimal path is equivalent to that of the baseline path.

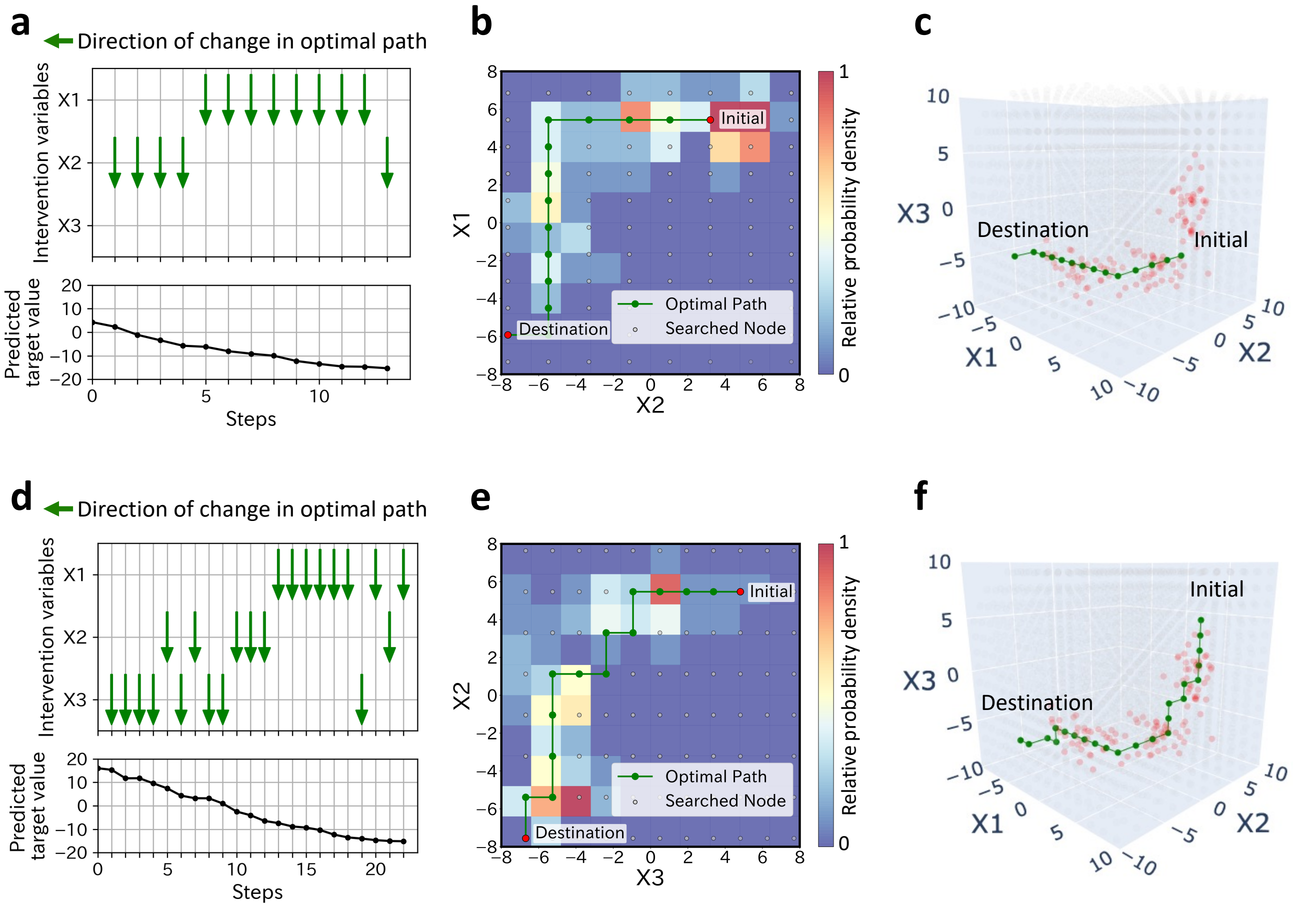

Supplementary Figure 10. Examples of actionable paths planned using random forest (RF) model on three-dimensional (3D) synthetic dataset. The optimal paths for improving the response variable predicted by the RF model are represented for the same instances selected on the application to XGBoost: instance A (**a–c**) and instance B (**d–f**). **a, d** The orders of changes in the explanatory variables in the optimal path and the accompanying changes in the predicted values. In the transition steps, the upward or downward arrow represents a unit increase or decrease in the explanatory variable, respectively. **b, e** 2D plots of the path. The 2D plots are shown regarding the selected two variables: X1 and X2 (**b**), and X2 and X3 (**e**). In the heatmaps, the probability density of the actual data, normalized by the panel with the maximum number of data, is expressed. **c, f** 3D plots of the path.

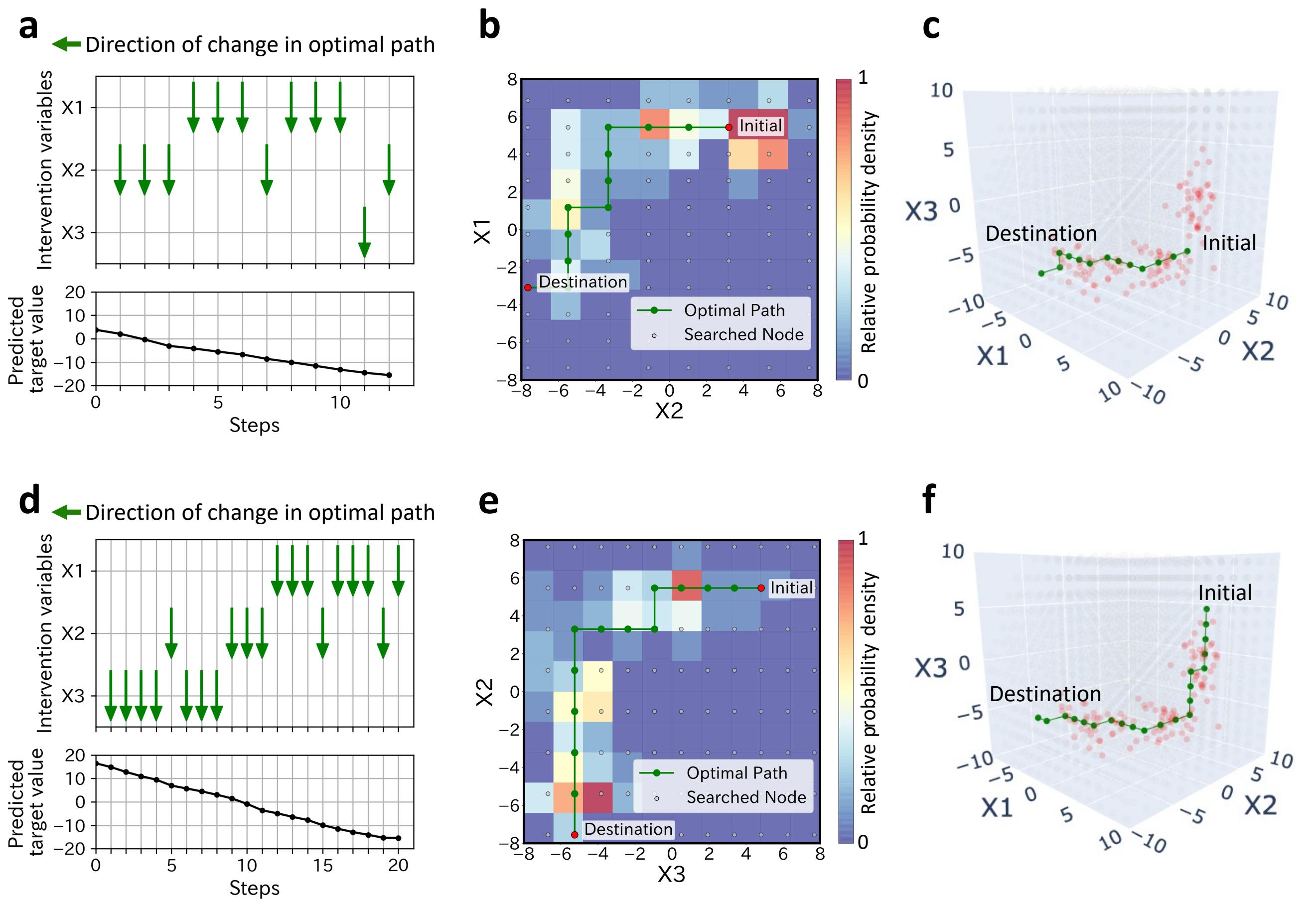

Supplementary Figure 11. Examples of actionable paths planned using support vector machine (SVM) model on three-dimensional (3D) synthetic dataset. The optimal paths for improving the response variable predicted by the SVM model are represented for the same instances selected on the application to XGBoost: instance A (**a–c**) and instance B (**d–f**). **a, d** The orders of changes in the explanatory variables in the optimal path and the accompanying changes in the predicted values. In the transition steps, the upward or downward arrow represents a unit increase or decrease in the explanatory variable, respectively. **b, e** 2D plots of the path. The 2D plots are shown regarding the selected two variables: X1 and X2 (**b**), and X2 and X3 (**e**). In the heatmaps, the probability density of the actual data, normalized by the panel with the maximum number of data, is expressed. **c, f** 3D plots of the path.

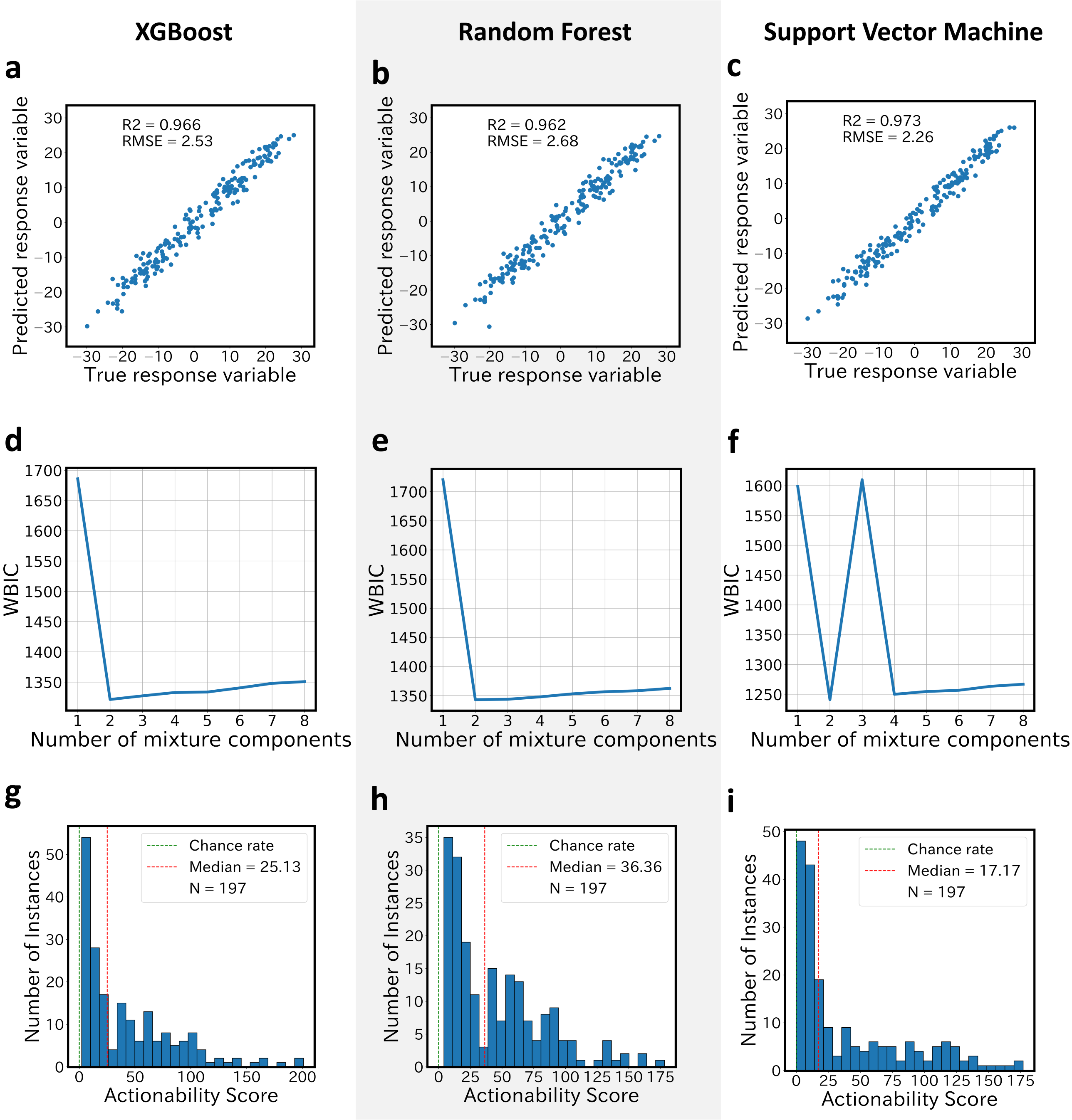

Supplementary Figure 12. Results of the proposed framework on five-dimensional (5D) synthetic dataset. The results of applying our framework to prediction models based on nonlinear machine learning (ML) algorithms are shown: XGBoost (**a, d, g**), random forest (**b, e, h**), and support vector machine (**c, f, i**). **a–c** Plots for prediction vs. true response variable. **d–f** Widely applicable Bayesian information criterion (WBIC) values of stochastic surrogate models with 1–8 mixture components. **g–i** Histogram of actionability scores with intervention variables based on data-driven selection at different instances. An actionability score of zero indicates that the actionability of the optimal path is equivalent to that of the baseline path.

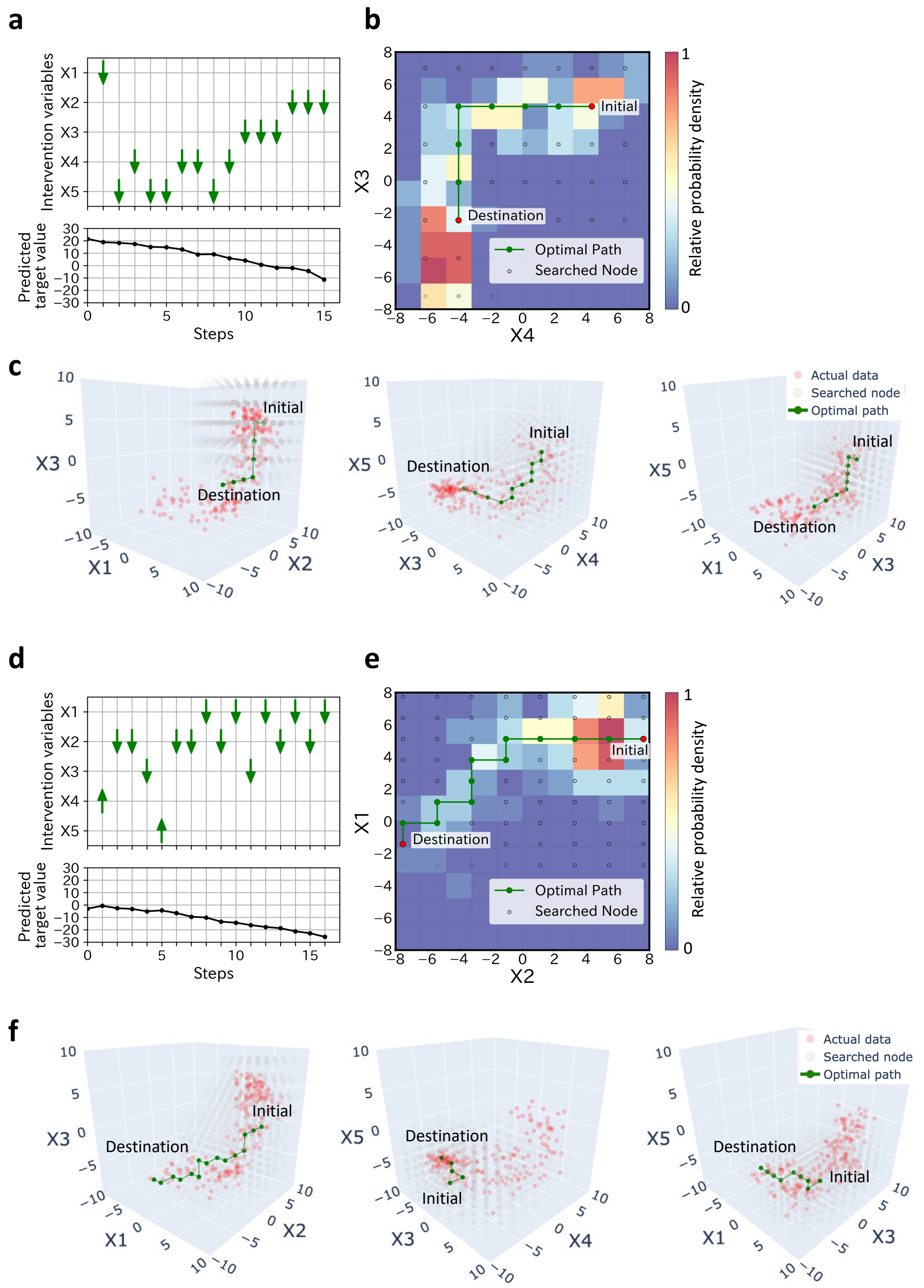

Supplementary Figure 13. Examples of actionable paths planned using XGBoost model on five-dimensional (5D) synthetic dataset. The optimal paths for improving the response variable predicted by the XGBoost model are represented for randomly selected two examples: instance C (**a–c**) and instance D (**d–f**). **a, d** The orders of changes in the explanatory variables in the optimal path and the accompanying changes in the predicted values. In the transition steps, the upward or downward arrow represents a unit increase or decrease in the explanatory variable, respectively. **b, e** 2D plots of the path. The 2D plots are shown regarding the selected two variables: X3 and X4 (**b**), and X1 and X2 (**e**). In the heatmaps, the probability density of the actual data, normalized by the panel with the maximum number of data, is expressed. **c, f** 3D plots of the path.

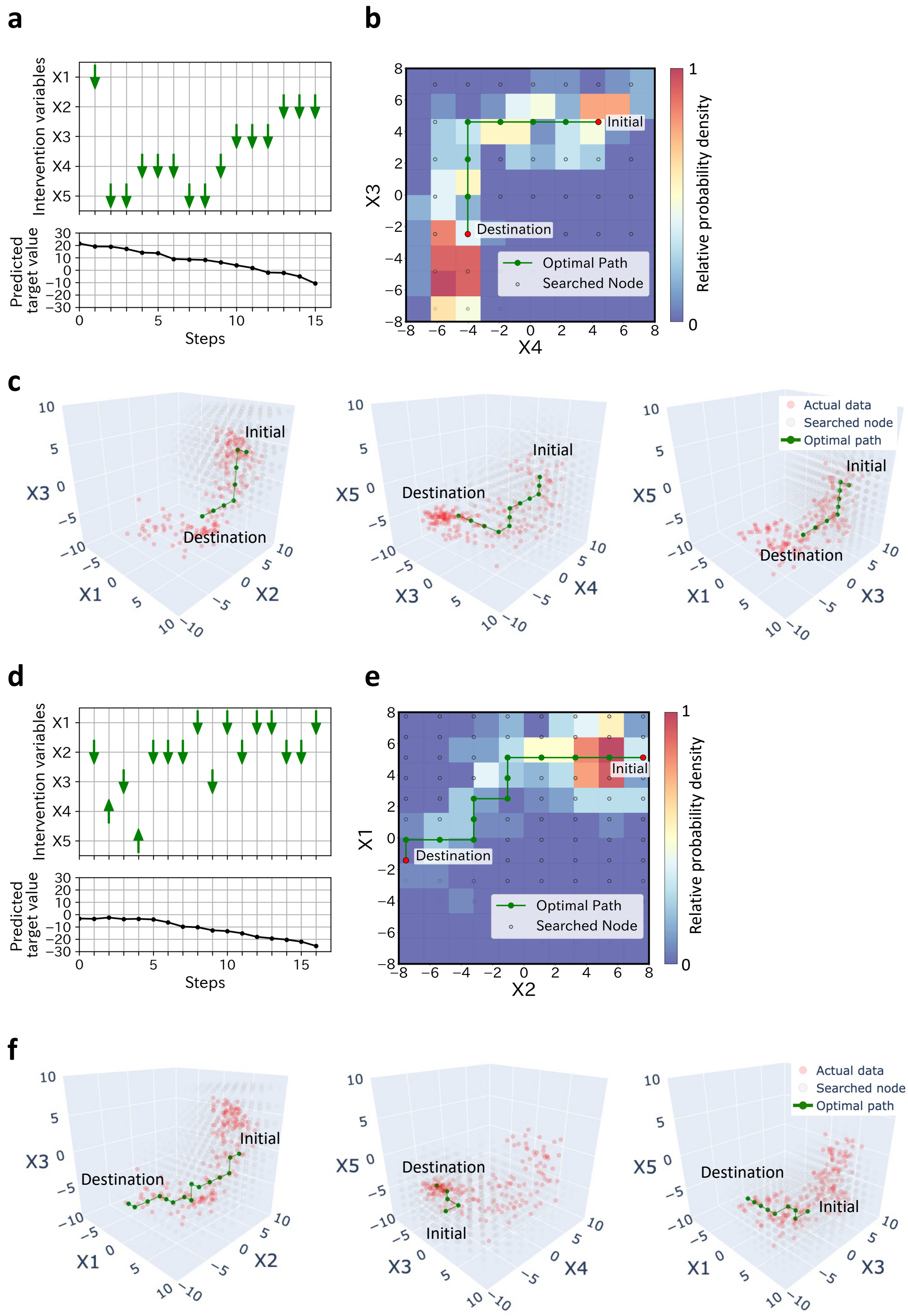

Supplementary Figure 14. Examples of actionable paths planned using random forest (RF) model on five-dimensional (5D) synthetic dataset. The optimal paths for improving the response variable predicted by the RF model are represented for the same instances selected on the application to XGBoost: instance C (**a–c**) and instance D (**d–f**). **a, d** The orders of changes in the explanatory variables in the optimal path and the accompanying changes in the predicted values. In the transition steps, the upward or downward arrow represents a unit increase or decrease in the explanatory variable, respectively. **b, e** 2D plots of the path. The 2D plots are shown regarding the selected two variables: X3 and X4 (**b**), and X1 and X2 (**e**). In the heatmaps, the probability density of the actual data, normalized by the panel with the maximum number of data, is expressed. **c, f** 3D plots of the path.

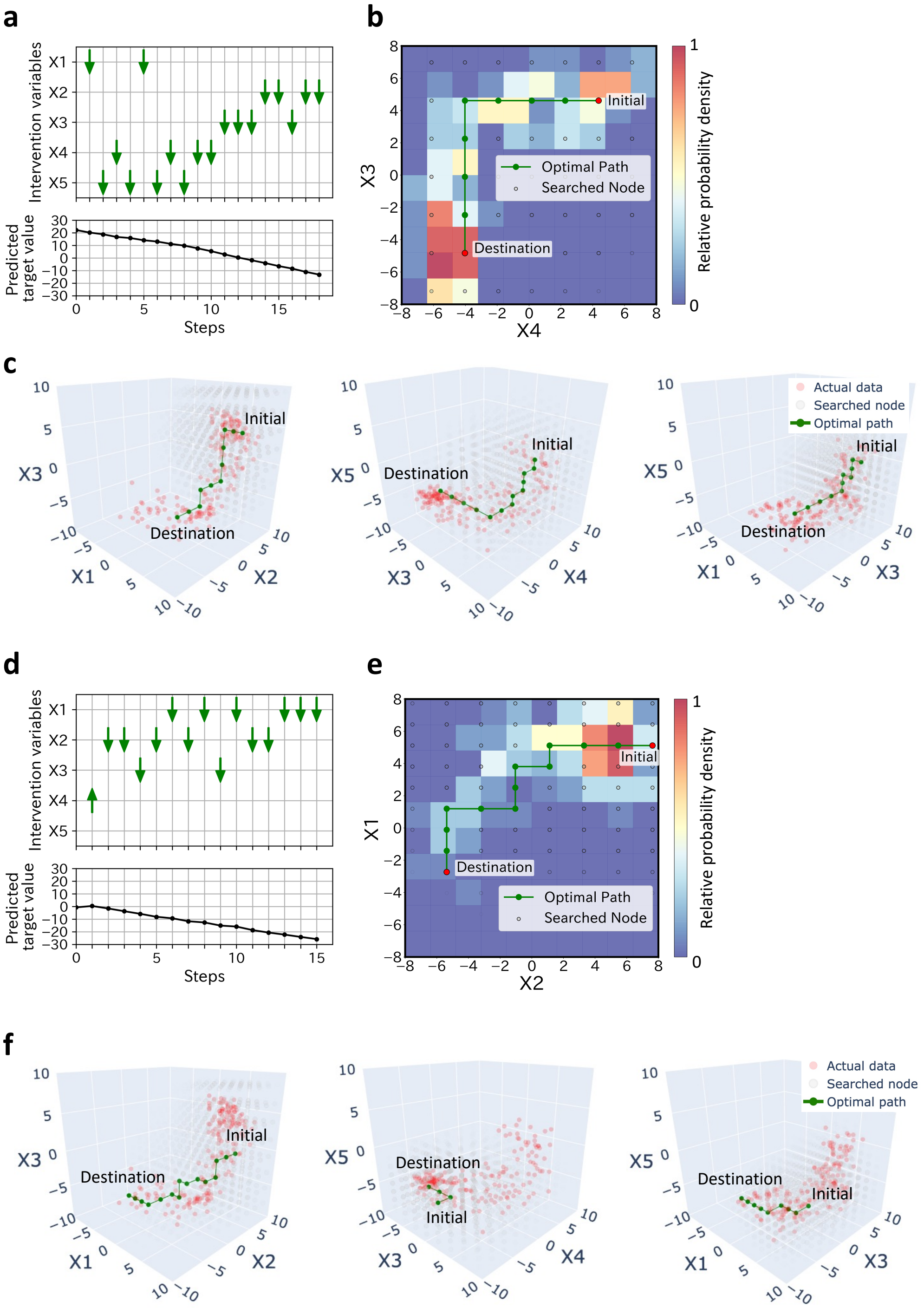

Supplementary Figure 15. Examples of actionable paths planned using support vector machine (SVM) model on five-dimensional (5D) synthetic dataset. The optimal paths for improving the response variable predicted by the SVM model are represented for the same instances selected on the application to XGBoost: instance C (**a–c**) and instance D (**d–f**). **a, d** The orders of changes in the explanatory variables in the optimal path and the accompanying changes in the predicted values. In the transition steps, the upward or downward arrow represents a unit increase or decrease in the explanatory variable, respectively. **B, e** 2D plots of the path. The 2D plots are shown regarding the selected two variables: X3 and X4 (**b**), and X1 and X2 (**e**). In the heatmaps, the probability density of the actual data, normalized by the panel with the maximum number of data, is expressed. **c, f** 3D plots of the path.

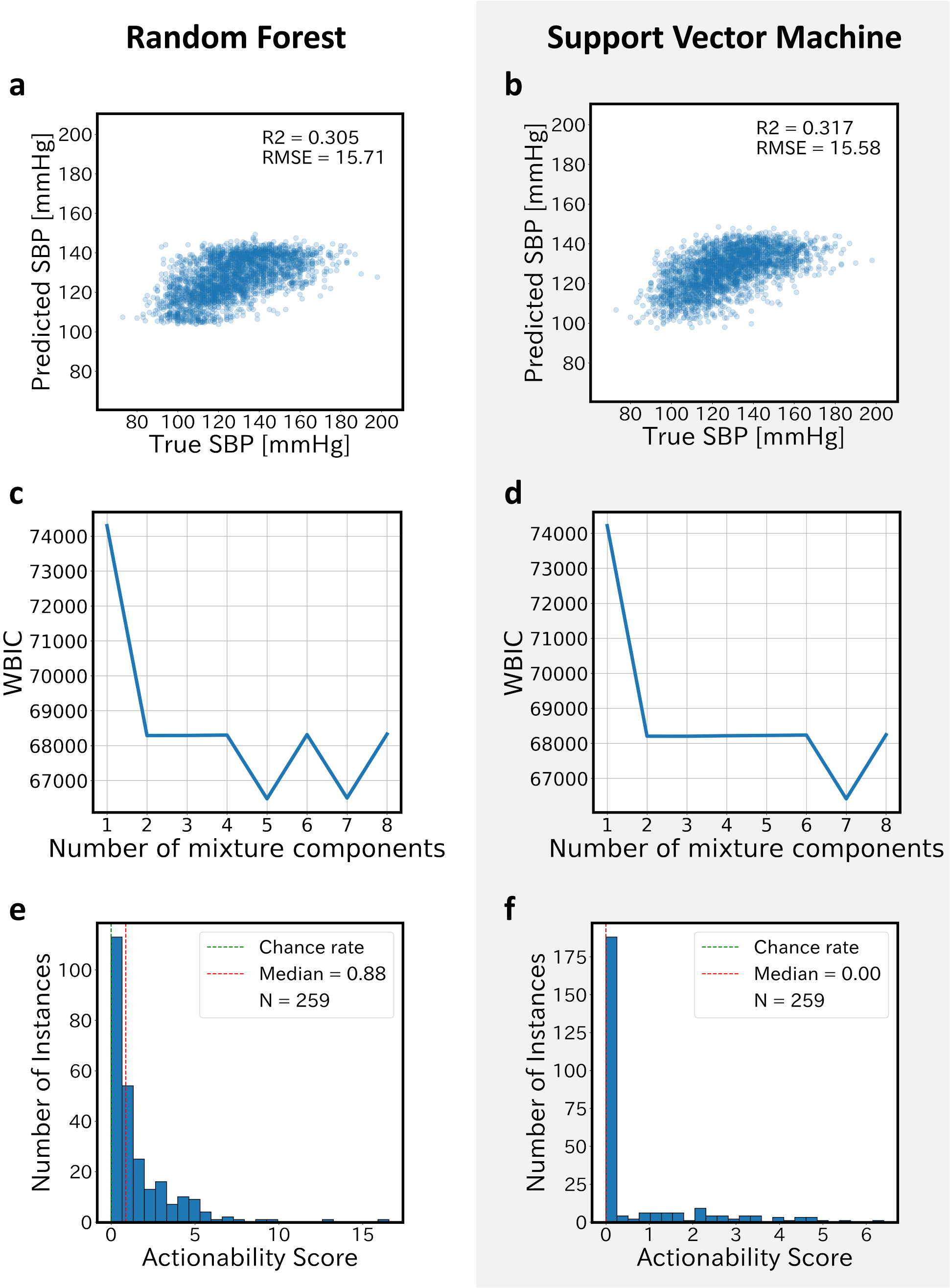

Supplementary Figure 16. Application of proposed framework on systolic blood pressure (SBP) regression task using different machine learning (ML) algorithms. The results of applying our framework to prediction models based on nonlinear ML algorithms other than XGBoost are shown: random forest (**a, c, e**) and support vector machine (**b, d, f**). **a, b** Plots for prediction vs. true response variable. **c, d** Widely applicable Bayesian information criterion (WBIC) values of stochastic surrogate models with 1–8 mixture components. **e, f** Histogram of actionability scores with intervention variables based on data-driven selection at different instances. An actionability score of zero indicates that the actionability of the optimal path is equivalent to that of the baseline path.

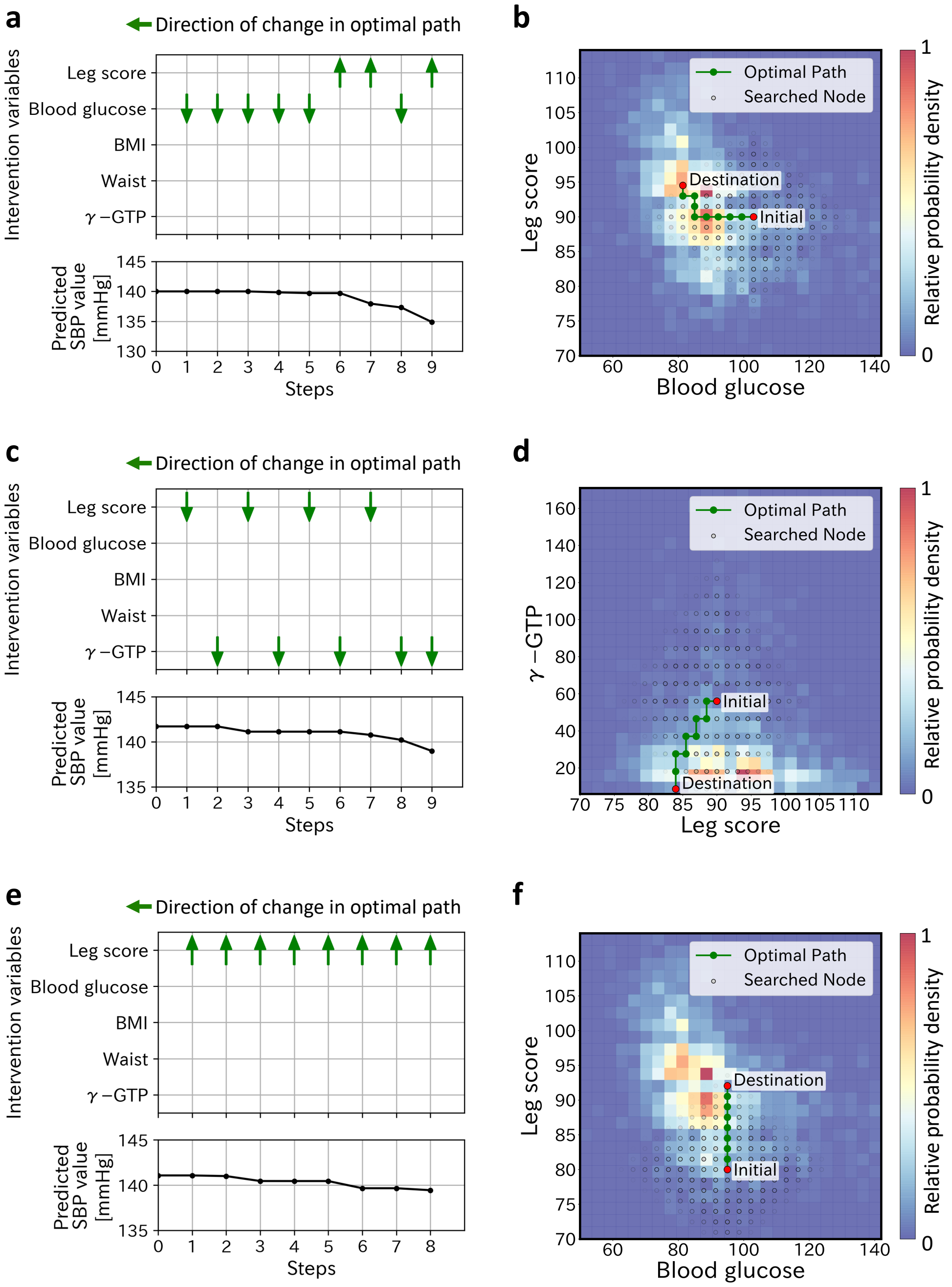

Supplementary Figure 17. Examples of personal actionable paths for treatment in systolic blood pressure (SBP) regression task using random forest (RF). The optimal paths for improving the response variable predicted by the RF model are represented for the same instances selected on the application to XGBoost: instance 1 (**a, b**), instance 2 (**c, d**), and instance 3 (**e, f**). **a, c, e** The orders of changes in the explanatory variables in the optimal path and the accompanying changes in the predicted values. In the transition steps, the upward or downward arrow represents a unit increase or decrease in the explanatory variable, respectively. **b, d, f** 2D plots of the path. The 2D plots are shown regarding the two influential variables in the optimal path: blood glucose and leg score (**b**), leg score and γ-GTP (**d**), and blood glucose and leg score (**f**). In the heatmaps, the probability density of the actual data, normalized by the panel with the maximum number of data, is expressed.

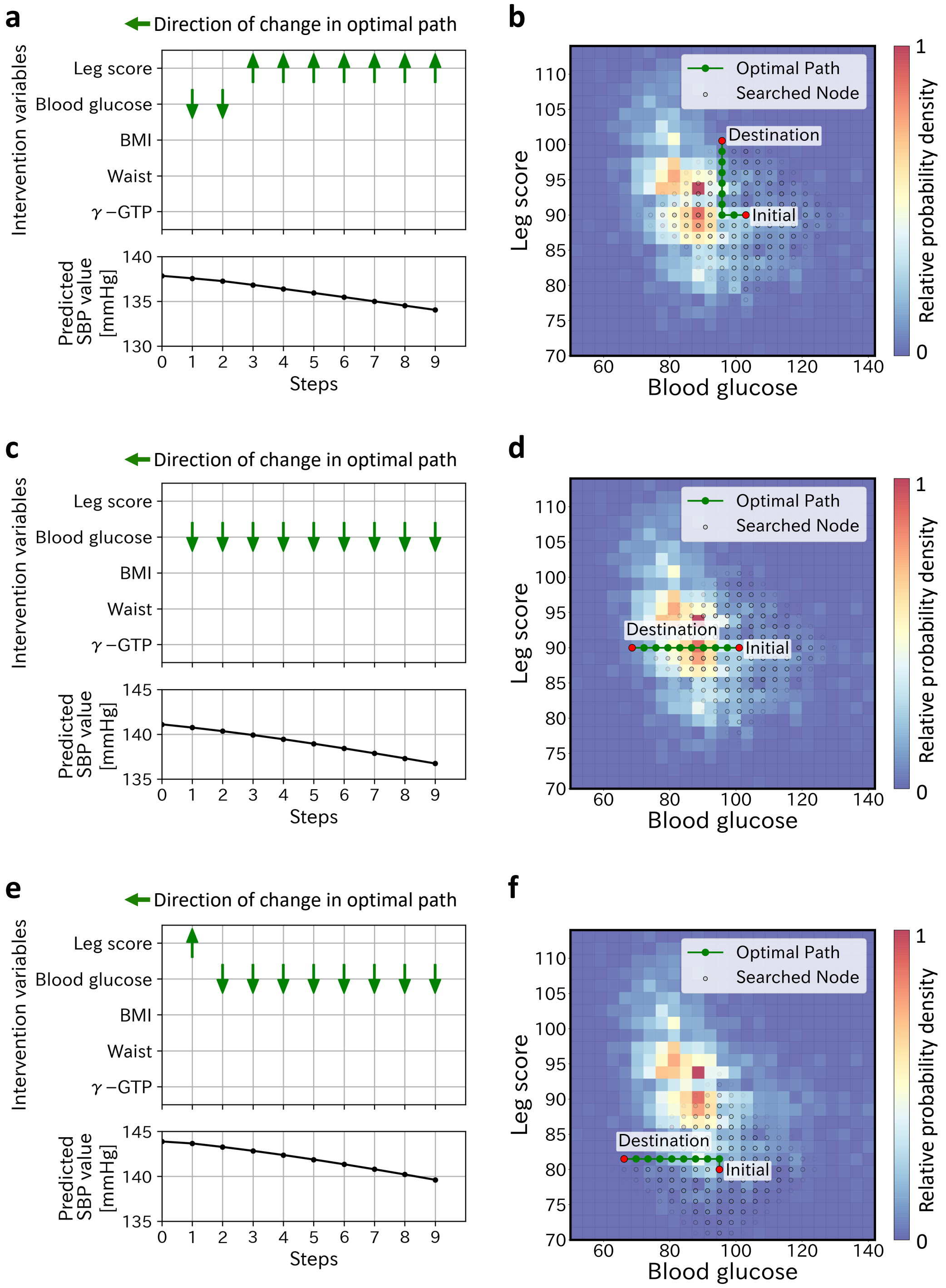

Supplementary Figure 18. Examples of personal actionable paths for treatment in systolic blood pressure (SBP) regression task using support vector machine (SVM). The optimal paths for improving the response variable predicted by the SVM model are represented for the same instances selected on the application to XGBoost: instance 1 (**a, b**), instance 2 (**c, d**), and instance 3 (**e, f**). **a, c, e** The orders of changes in the explanatory variables in the optimal path and the accompanying changes in the predicted values. In the transition steps, the upward or downward arrow represents a unit increase or decrease in the explanatory variable, respectively. **b, d, f** 2D plots of the path regarding blood glucose and leg score. In the heatmaps, the probability density of the actual data, normalized by the panel with the maximum number of data, is expressed.

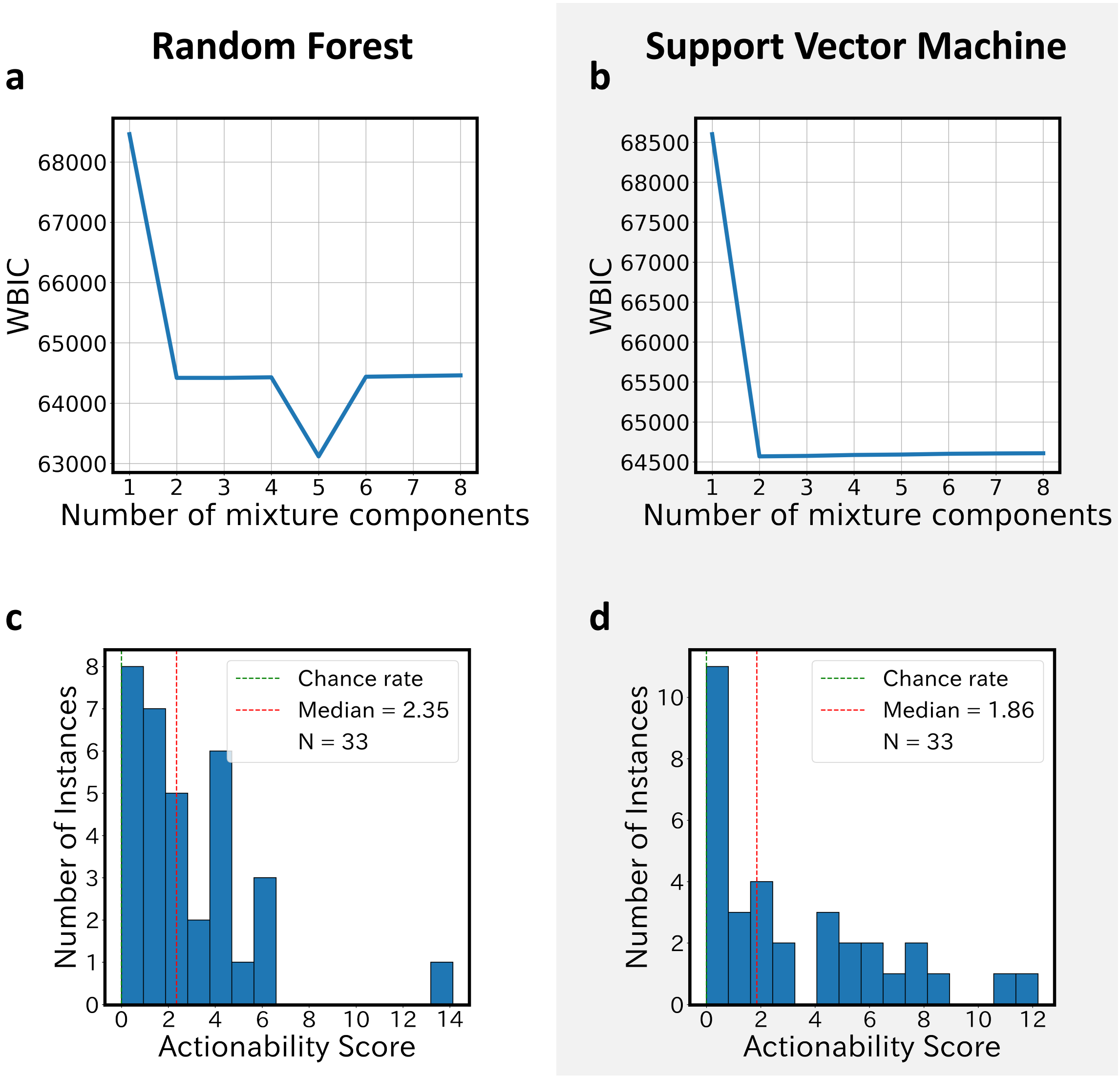

Supplementary Figure 19. Application of proposed framework on chronic kidney disease (CKD) risk classification task using different machine learning (ML) algorithms. The results of applying our framework to prediction models based on nonlinear ML algorithms other than XGBoost are shown: random forest (**a, c**) and support vector machine (**b, d**). **a, b** Widely applicable Bayesian information criterion (WBIC) values of stochastic surrogate models with 1–8 mixture components. **c, d** Histogram of actionability scores with intervention variables based on data-driven selection at different instances. An actionability score of zero indicates that the actionability of the optimal path is equivalent to that of the baseline path.

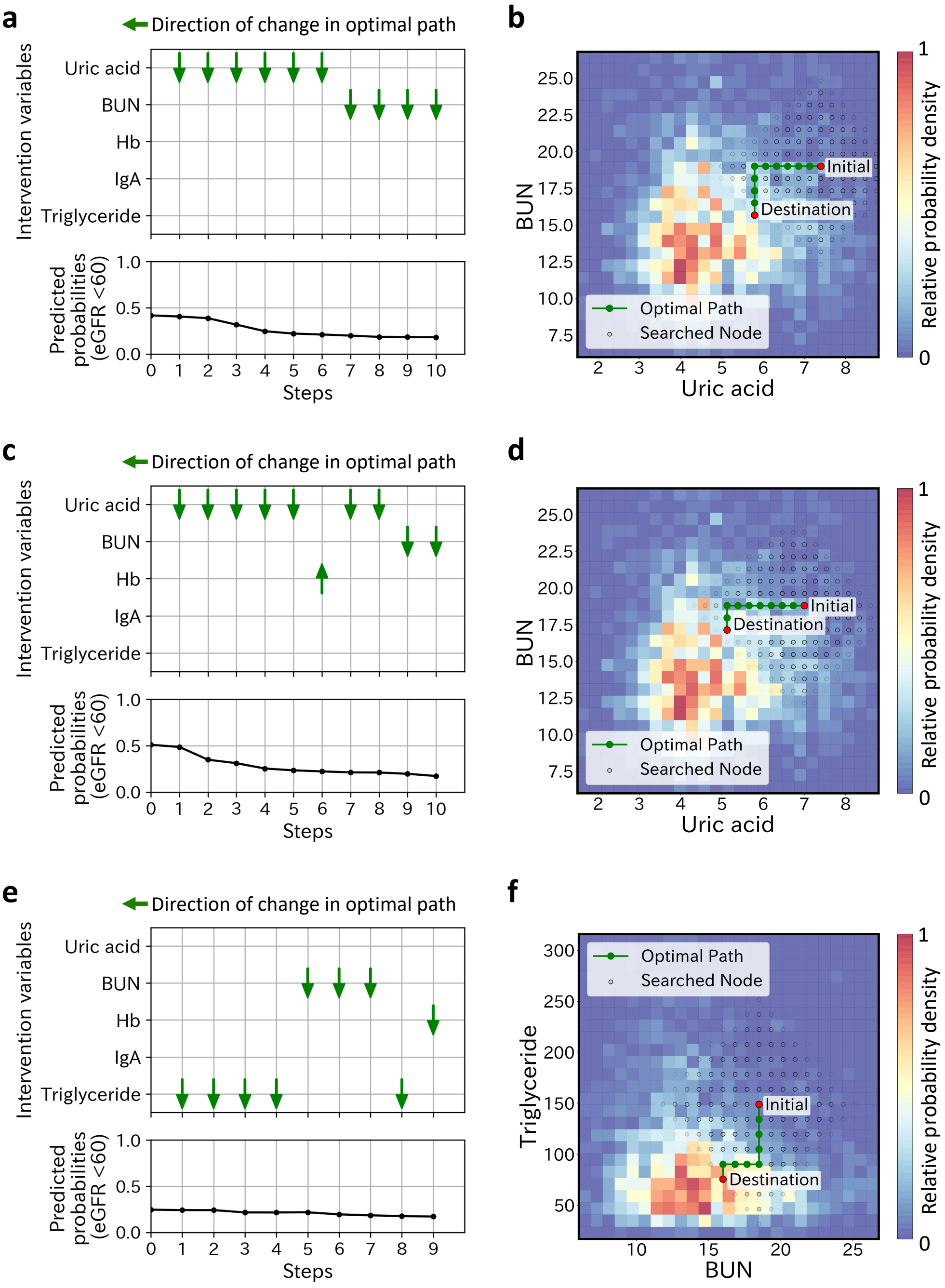

Supplementary Figure 20. Examples of personal actionable paths for treatment in chronic kidney disease (CKD) risk classification task using random forest (RF). The optimal paths for improving the response variable predicted by the RF model are represented for the same instances selected on the application to XGBoost: instance 4 (**a, b**), instance 5 (**c, d**), and instance 6 (**e, f**). **a, c, e** The orders of changes in the explanatory variables in the optimal path and the accompanying changes in the predicted values. In the transition steps, the upward or downward arrow represents a unit increase or decrease in the explanatory variable, respectively. **b, d, f** 2D plots of the path. The 2D plots are shown regarding the two influential variables in the optimal path: uric acid and blood urea nitrogen (BUN) (**b, d**), and triglyceride and BUN (**f**). In the heatmaps, the probability density of the actual data, normalized by the panel with the maximum number of data, is expressed.

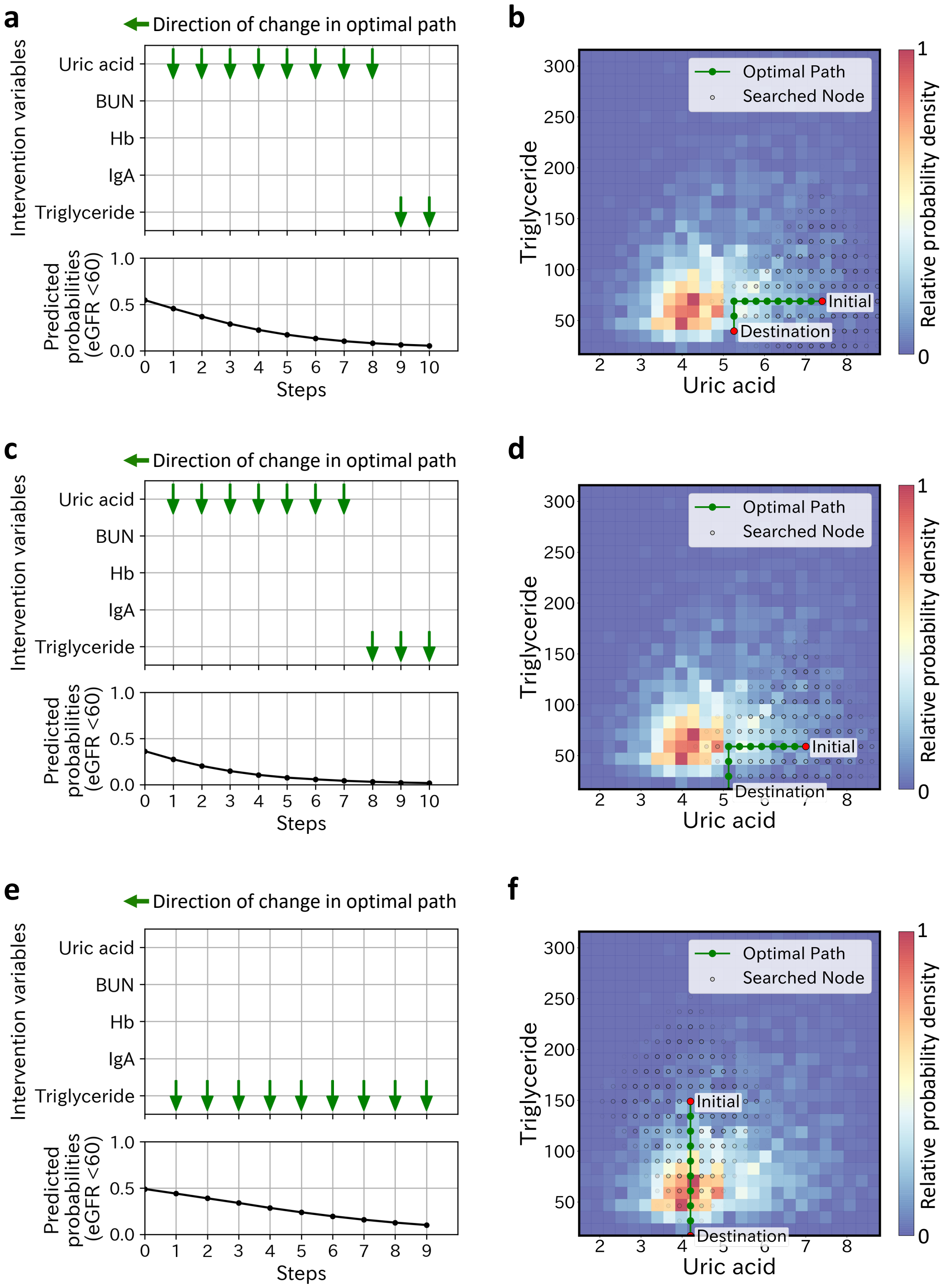

Supplementary Figure 21. Examples of personal actionable paths for treatment in chronic kidney disease (CKD) risk classification task using support vector machine (SVM). The optimal paths for improving the response variable predicted by the SVM model are represented for the same instances selected on the application to XGBoost: instance 4 (**a, b**), instance 5 (**c, d**), and instance 6 (**e, f**). **a, c, e** The orders of changes in the explanatory variables in the optimal path and the accompanying changes in the predicted values. In the transition steps, the upward or downward arrow represents a unit increase or decrease in the explanatory variable, respectively. **b, d, f** 2D plots of the path. The 2D plots are shown regarding triglyceride and uric acid. In the heatmaps, the probability density of the actual data, normalized by the panel with the maximum number of data, is expressed.

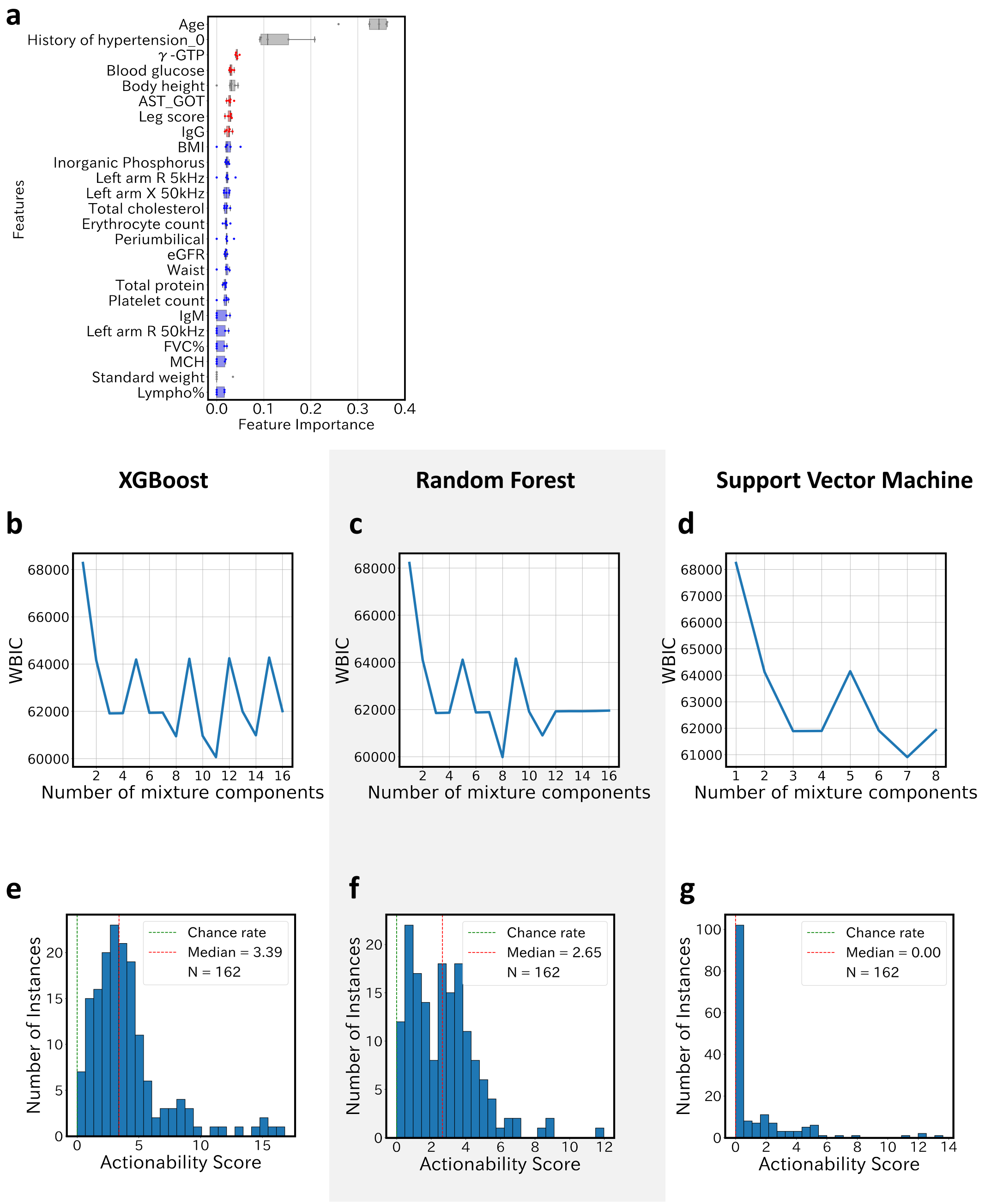

Supplementary Figure 22. Results of the proposed framework on hypertension risk classification task. a Feature importance. These 25 features were selected by recursive feature elimination (RFE). RFE was performed with five-fold cross-validation, and the feature importance when 25 variables remained is shown for each fold (n = 5). The plot color represents the following: red: intervention variables, gray: variables which cannot be intervened, and blue: other variables. In box-plot, center line represents median; box limits, upper and lower quartiles; whiskers, 1.5x interquartile range. Details of features are described in Supplementary Data 1. **b–g** The results of applying our framework to prediction models based on nonlinear machine learning (ML) algorithms: XGBoost (**b**, **e**), random forest (**c**, **f**), and support vector machine (**d**, **g**). **b–d** Widely applicable Bayesian information criterion (WBIC) values of stochastic surrogate models. **e–g** Histogram of actionability scores. An actionability score of zero indicates that the actionability of the optimal path is equivalent to that of the baseline path.

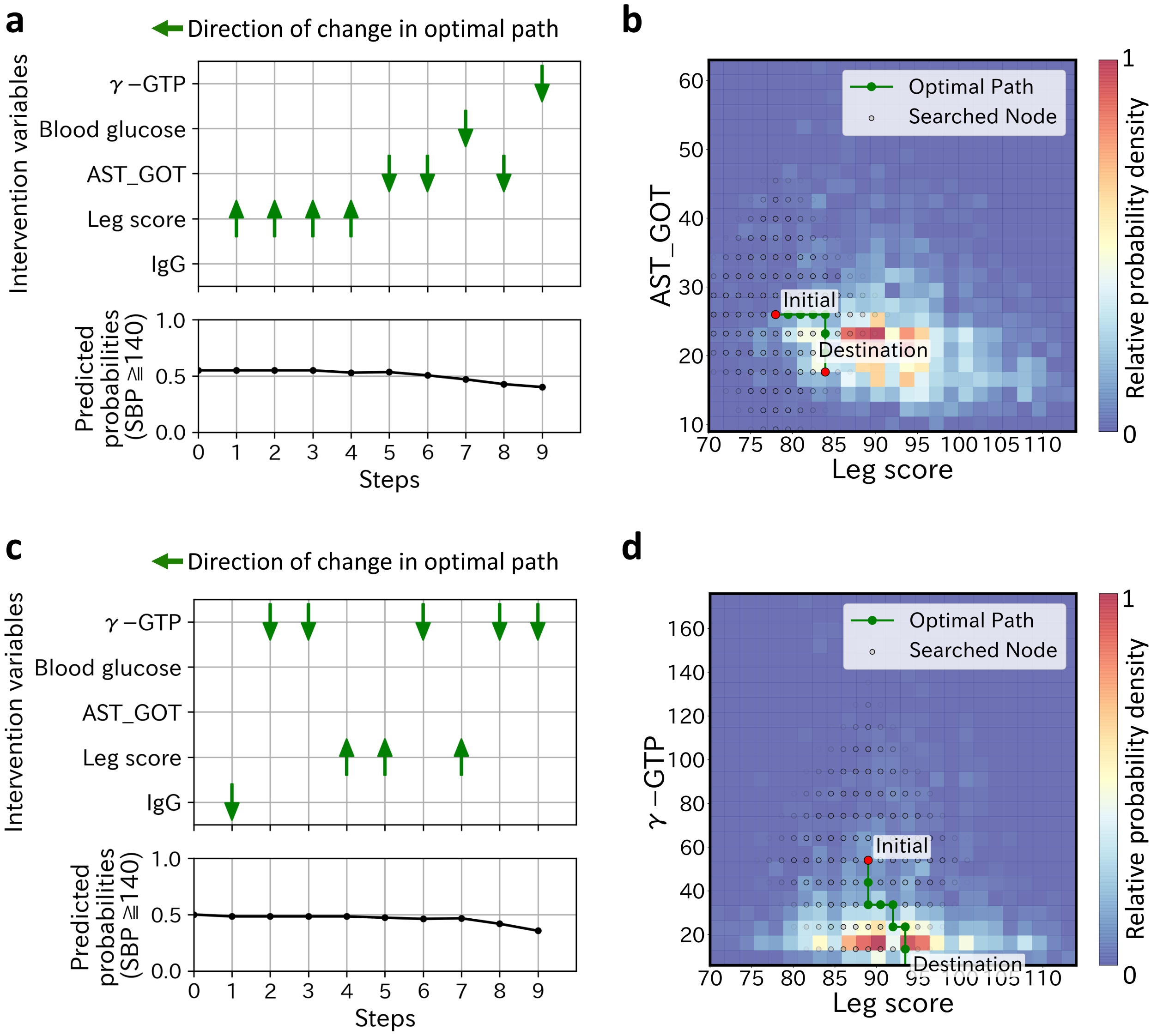

Supplementary Figure 23. Examples of personal actionable paths for treatment in hypertension risk classification task using XGBoost. The optimal paths for improving the response variable predicted by the XGBoost model are represented for randomly selected two examples: instance 7 (**a, b**) and instance 8 (**c, d**). **a, c** The orders of changes in the explanatory variables in the optimal path and the accompanying changes in the predicted values. In the transition steps, the upward or downward arrow represents a unit increase or decrease in the explanatory variable, respectively. **b, d** 2D plots of the path. The 2D plots are shown regarding the two influential variables in the optimal path: aspartate transaminase (AST [GOT]) and leg score (**b**), and gamma glutamyl transferase (g-GTP) and leg score (**d**). In the heatmaps, the probability density of the actual data, normalized by the panel with the maximum number of data, is expressed.

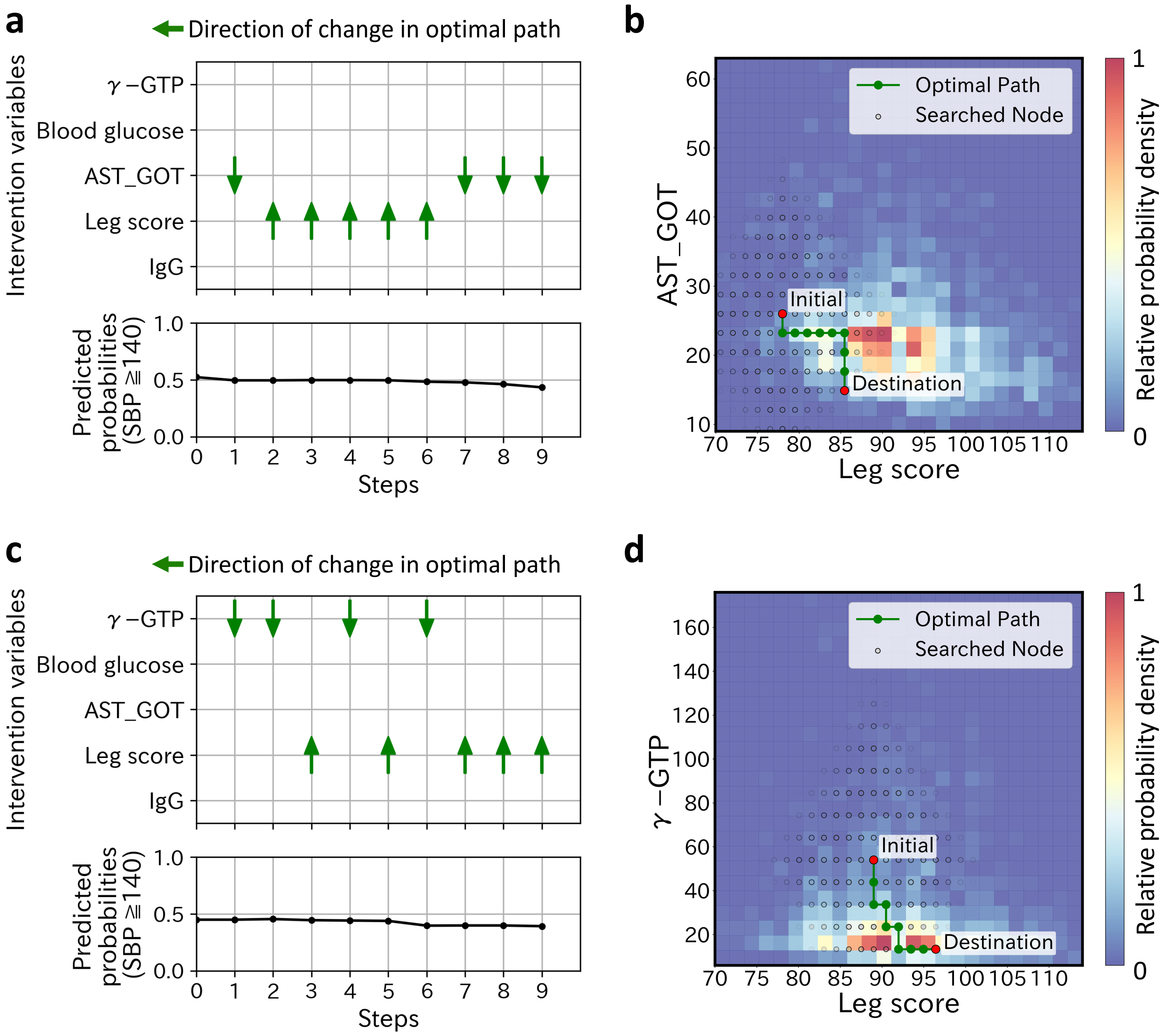

Supplementary Figure 24. Examples of personal actionable paths for treatment in hypertension risk classification task using random forest (RF). The optimal paths for improving the response variable predicted by the RF model are represented for the same instances selected on the application to XGBoost: instance 7 (**a, b**) and instance 8 (**c, d**). **a, c** The orders of changes in the explanatory variables in the optimal path and the accompanying changes in the predicted values. In the transition steps, the upward or downward arrow represents a unit increase or decrease in the explanatory variable, respectively. **b, d** 2D plots of the path. The 2D plots are shown regarding the two influential variables in the optimal path: aspartate transaminase (AST [GOT]) and leg score (**b**), and gamma glutamyl transferase (g-GTP) and leg score (**d**). In the heatmaps, the probability density of the actual data, normalized by the panel with the maximum number of data, is expressed.

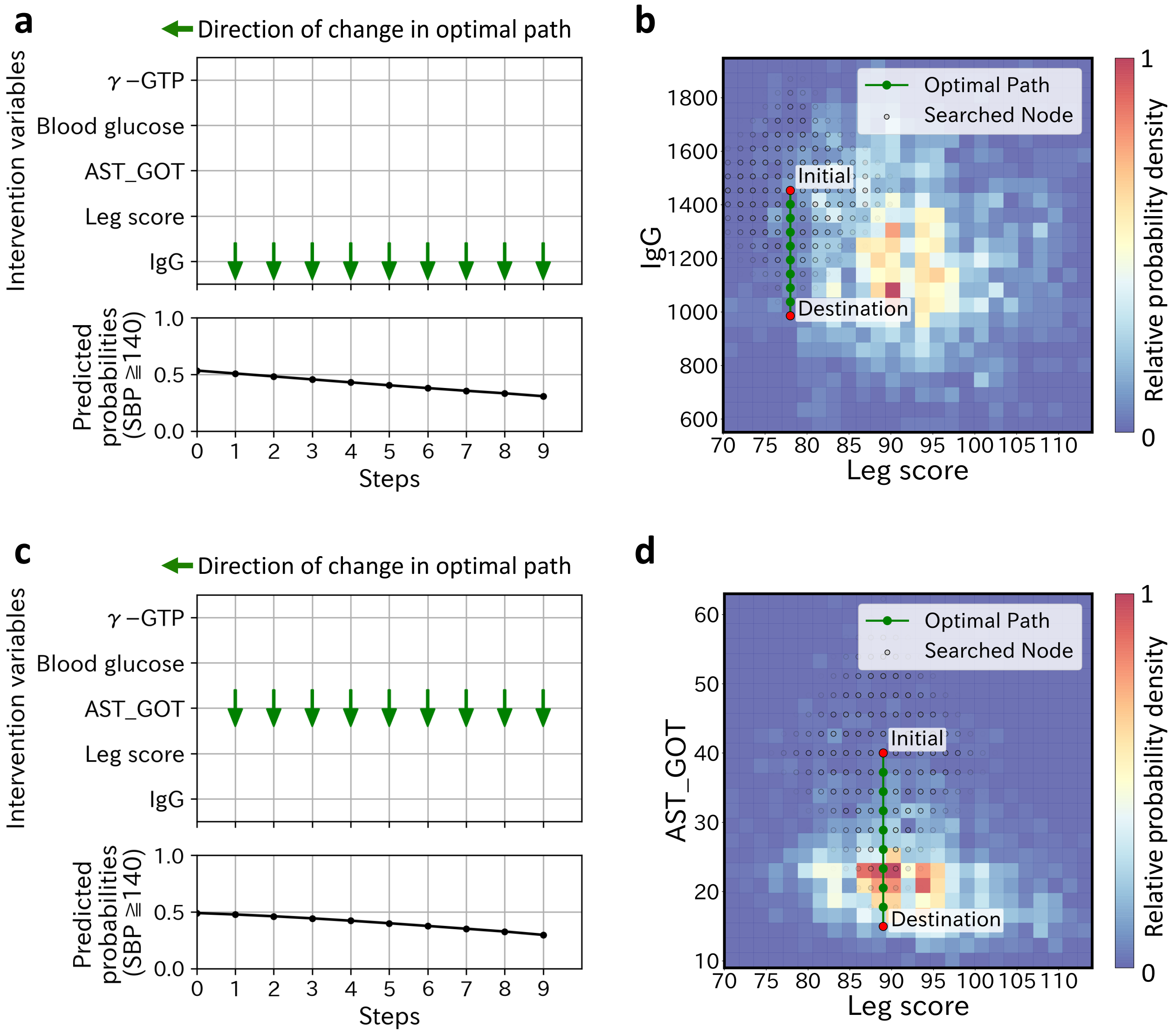

Supplementary Figure 25. Examples of personal actionable paths for treatment in hypertension risk classification task using support vector machine (SVM). The optimal paths for improving the response variable predicted by the SVM model are represented for the same instances selected on the application to XGBoost: instance 7 (**a, b**) and instance 8 (**c, d**). **a, c** The orders of changes in the explanatory variables in the optimal path and the accompanying changes in the predicted values. In the transition steps, the upward or downward arrow represents a unit increase or decrease in the explanatory variable, respectively. **b, d** 2D plots of the path. The 2D plots are shown regarding the two influential variables in the optimal path: immunoglobulin G (IgG) and leg score (**b**), and aspartate transaminase (AST [GOT]) and leg score (**d**). In the heatmaps, the probability density of the actual data, normalized by the panel with the maximum number of data, is expressed.

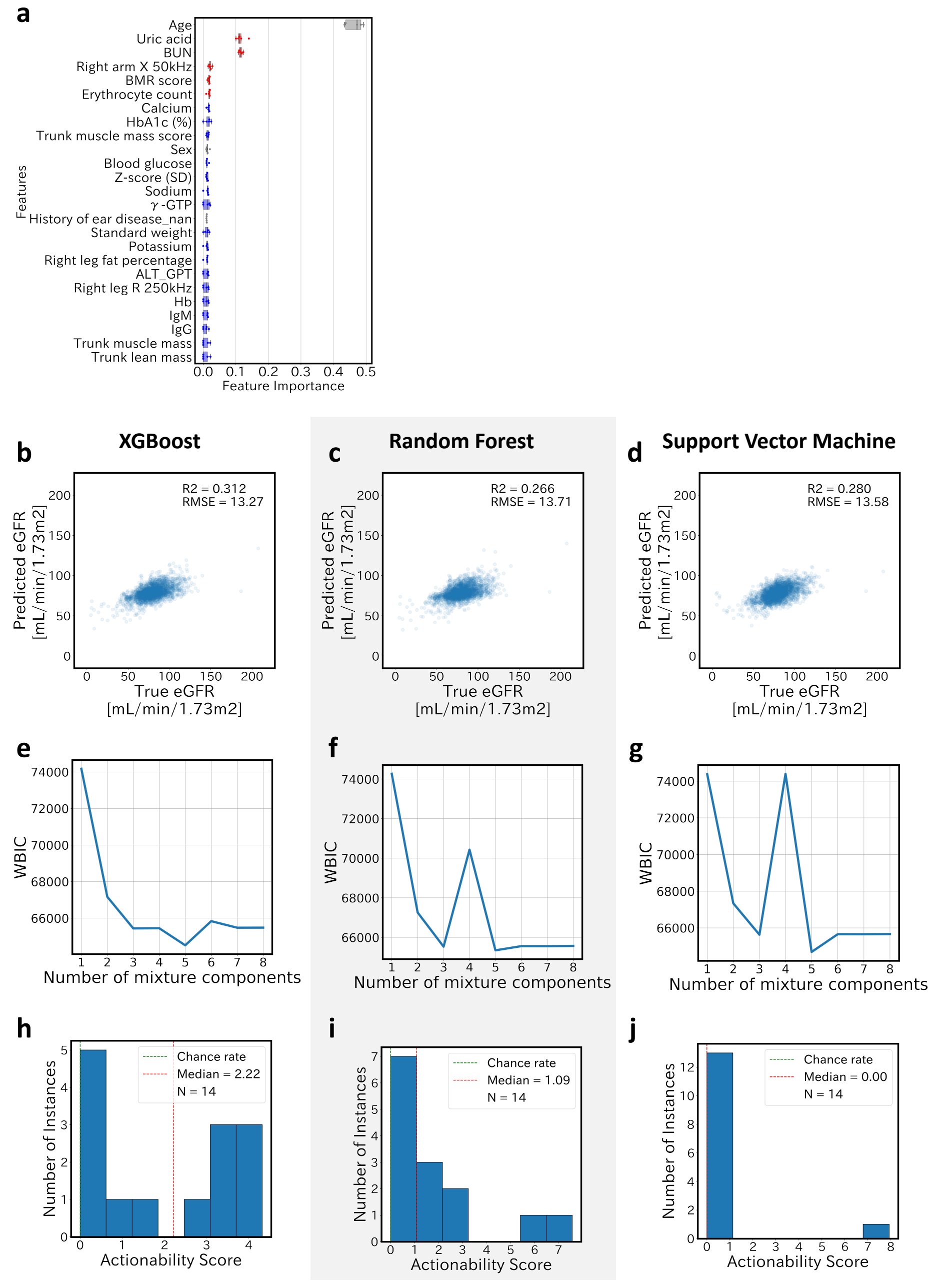

Supplementary Figure 26. Results of the proposed framework estimated glomerular filtration rate (eGFR) regression task. a Feature importance. These 25 features were selected by recursive feature elimination (RFE). RFE was performed with five-fold cross-validation, and the feature importance when 25 variables remained is shown for each fold ($n = 5$). The plot color represents the following: red: intervention variables, gray: variables which cannot be intervened, and blue: other variables. In box-plot, center line represents median; box limits, upper and lower quartiles; whiskers, 1.5x interquartile range. Details of features are described in Supplementary Data 1. **b–j** The results of applying our framework to prediction models based on nonlinear machine learning (ML) algorithms: XGBoost (**b, e, h**), random forest (**c, f, i**), and support vector machine (**d**, **g**, **j**). **b–d** Plots for prediction vs. true response variable. **e–g** Widely applicable Bayesian information criterion (WBIC) values of stochastic surrogate models with 1–8 mixture components. **h–j** Histogram of actionability scores. An actionability score of zero indicates that the actionability of the optimal path is equivalent to that of the baseline path.

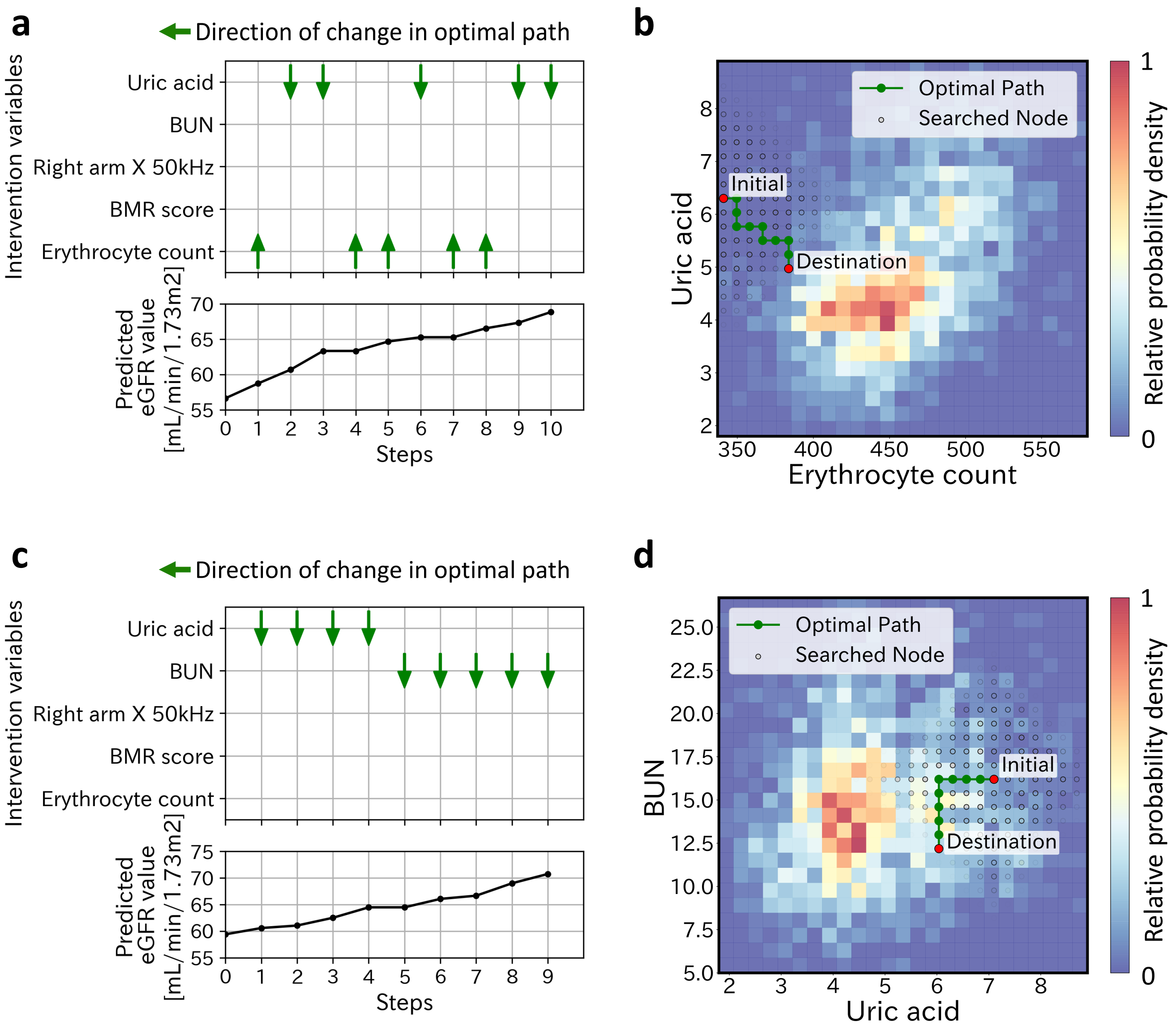

Supplementary Figure 27. Examples of personal actionable paths for treatment in estimated glomerular filtration rate (eGFR) regression task using XGBoost. The optimal paths for improving the response variable predicted by the XGBoost model are represented for randomly selected two examples: instance 9 (**a, b**) and instance 10 (**c, d**). **a, c** The orders of changes in the explanatory variables in the optimal path and the accompanying changes in the predicted values. In the transition steps, the upward or downward arrow represents a unit increase or decrease in the explanatory variable, respectively. **b, d** 2D plots of the path. The 2D plots are shown regarding the two influential variables in the optimal path: uric acid and erythrocyte count (**b**), and uric acid and blood urea nitrogen (BUN) (**d**). In the heatmaps, the probability density of the actual data, normalized by the panel with the maximum number of data, is expressed.

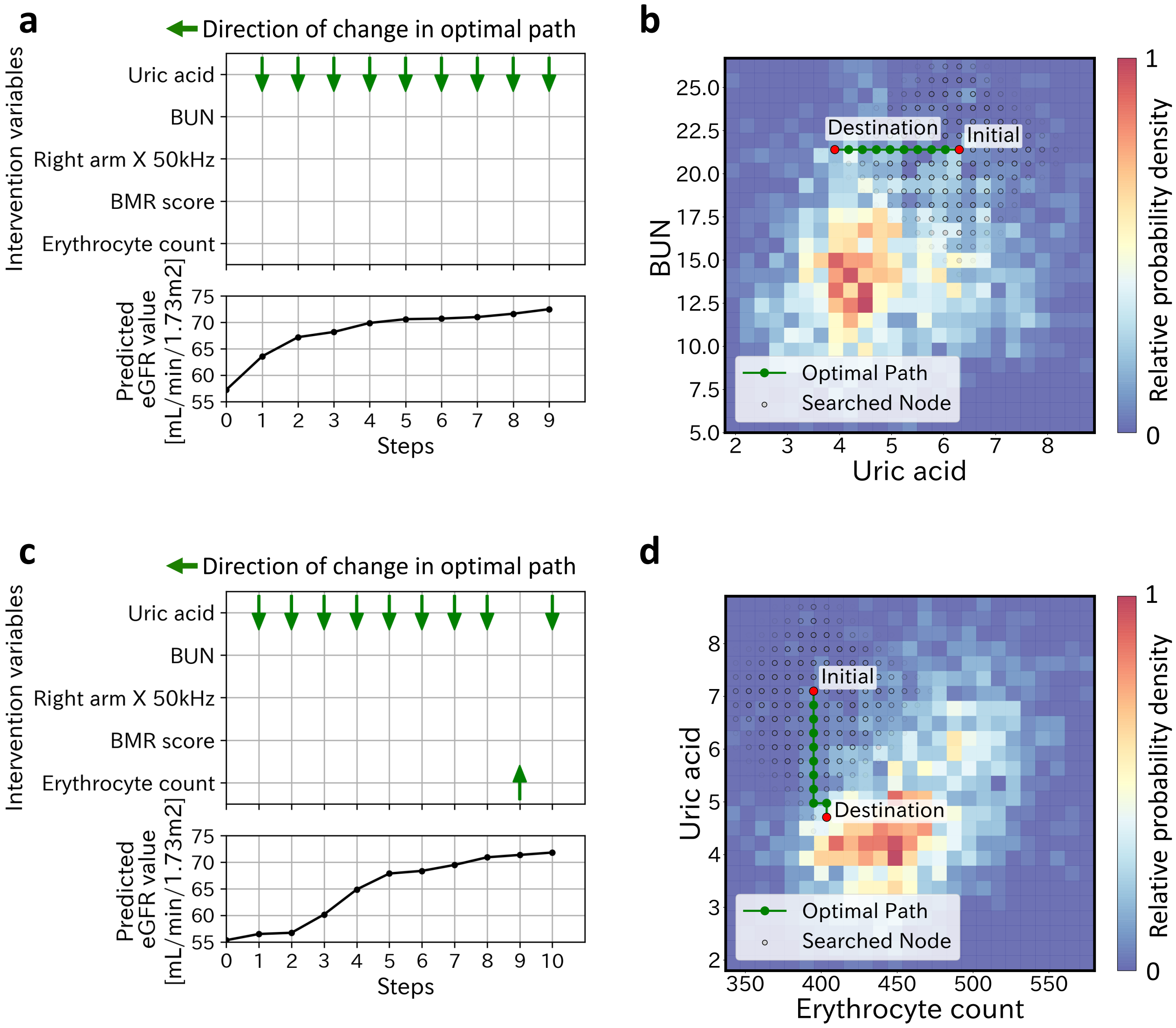

Supplementary Figure 28. Examples of personal actionable paths for treatment in estimated glomerular filtration rate (eGFR) regression task using random forest (RF). The optimal paths for improving the response variable predicted by the RF model are represented for the same instances selected on the application to XGBoost: instance 9 (**a, b**) and instance 10 (**c, d**). **a, c** The orders of changes in the explanatory variables in the optimal path and the accompanying changes in the predicted values. In the transition steps, the upward or downward arrow represents a unit increase or decrease in the explanatory variable, respectively. **b, d** 2D plots of the path. The 2D plots are shown regarding the two influential variables in the optimal path: uric acid and blood urea nitrogen (BUN) (**b**), and uric acid and erythrocyte count (**d**). In the heatmaps, the probability density of the actual data, normalized by the panel with the maximum number of data, is expressed.

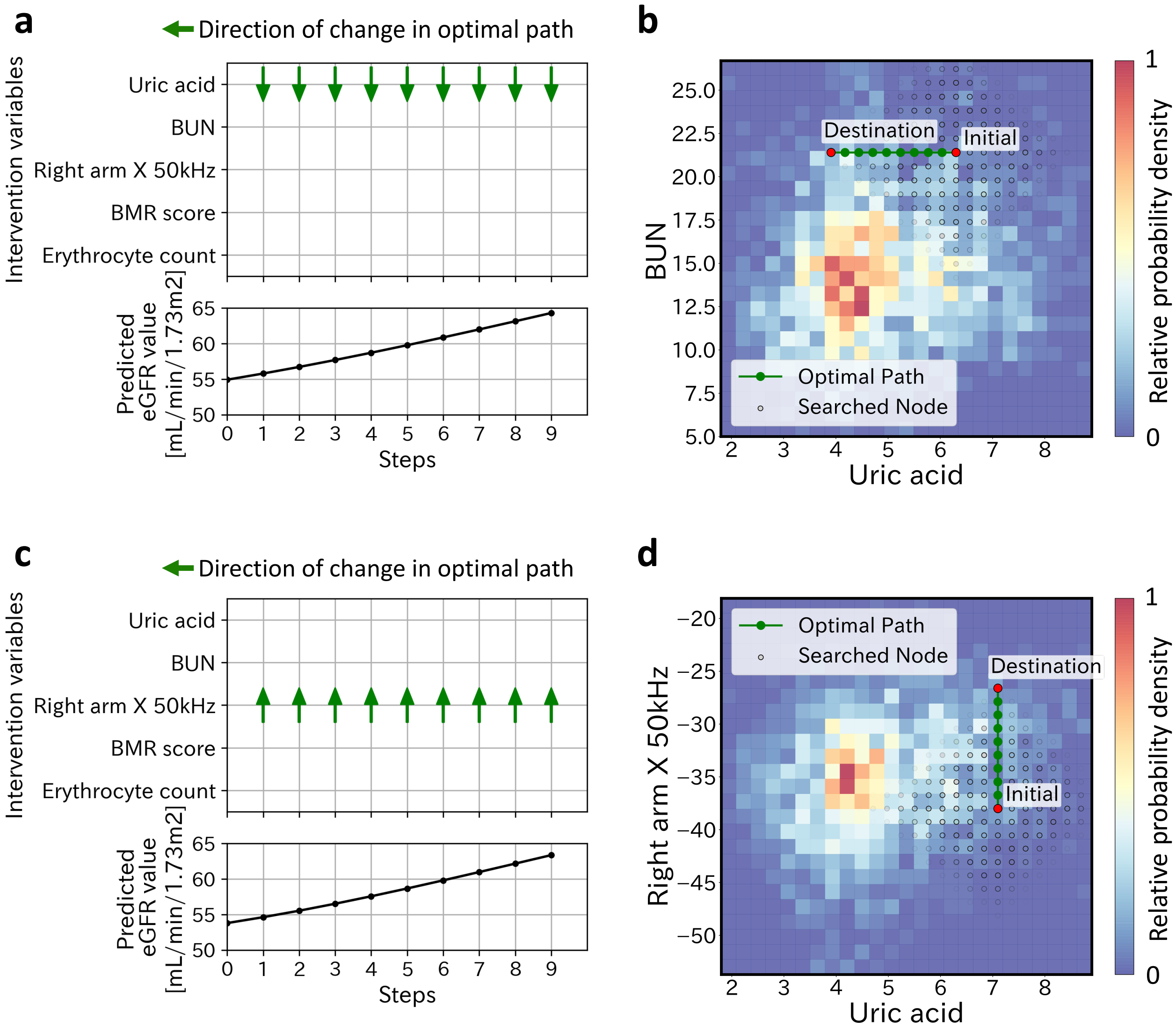

Supplementary Figure 29. Examples of personal actionable paths for treatment in estimated glomerular filtration rate (eGFR) regression task using support vector machine (SVM). The optimal paths for improving the response variable predicted by the SVM model are represented for the same instances selected on the application to XGBoost: instance 9 (**a, b**) and instance 10 (**c, d**). **a, c** The orders of changes in the explanatory variables in the optimal path and the accompanying changes in the predicted values. In the transition steps, the upward or downward arrow represents a unit increase or decrease in the explanatory variable, respectively. **b, d** 2D plots of the path. The 2D plots are shown regarding the two influential variables in the optimal path: uric acid and blood urea nitrogen (BUN) (**b**), and uric acid and right arm X 50 kHz (**d**). In the heatmaps, the probability density of the actual data, normalized by the panel with the maximum number of data, is expressed.

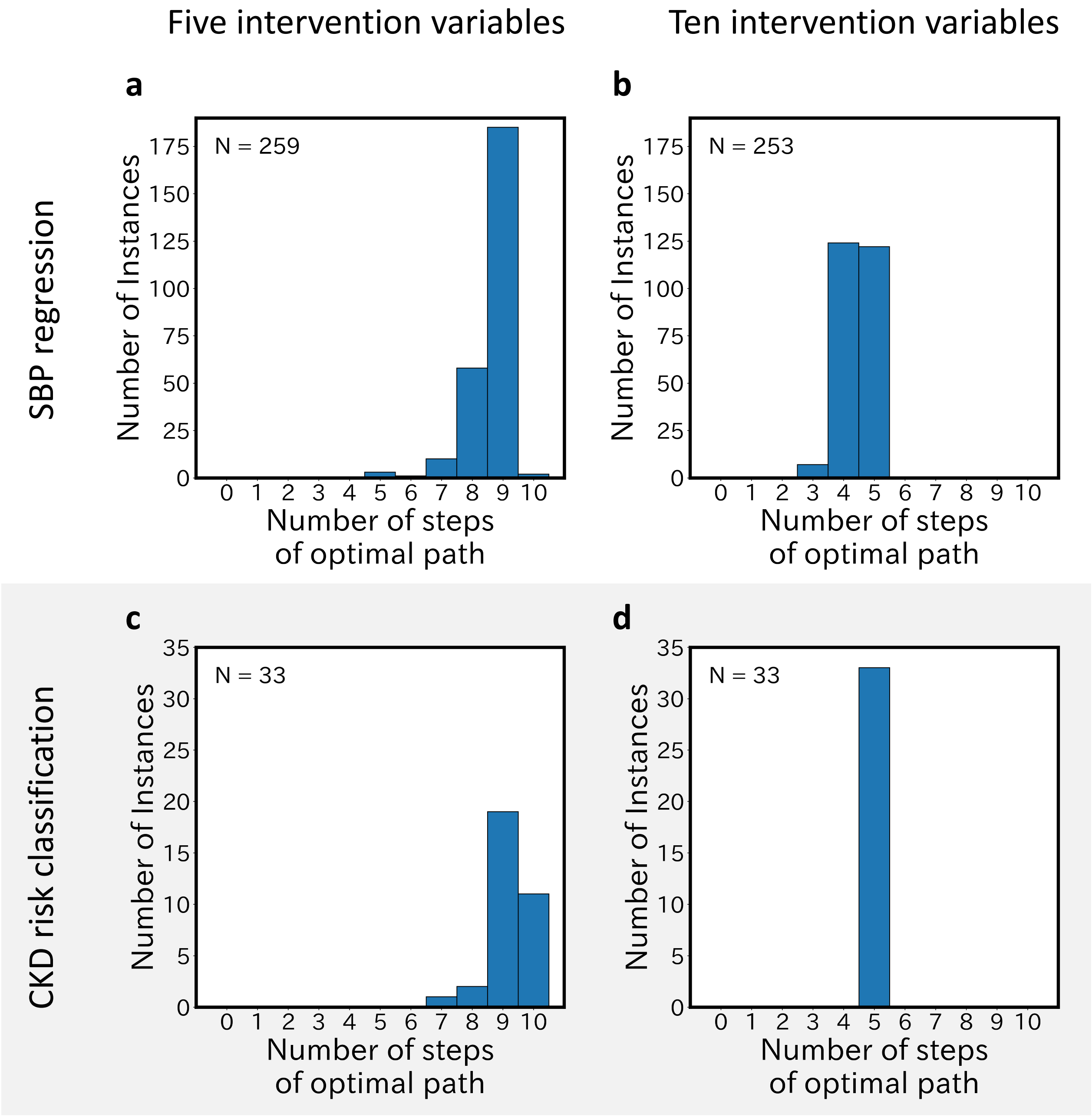

Supplementary Figure 30. Number of steps in optimal paths. The number of steps in the optimal paths for each instance is shown. **a, b** Top-five (**a**) or top-ten (**b**) important features were selected as intervention variables on systolic blood pressure (SBP) regression task. **c, d** Top-five (**c**) or top-ten (**d**) important features were selected as intervention variables on chronic kidney disease (CKD) risk classification task.

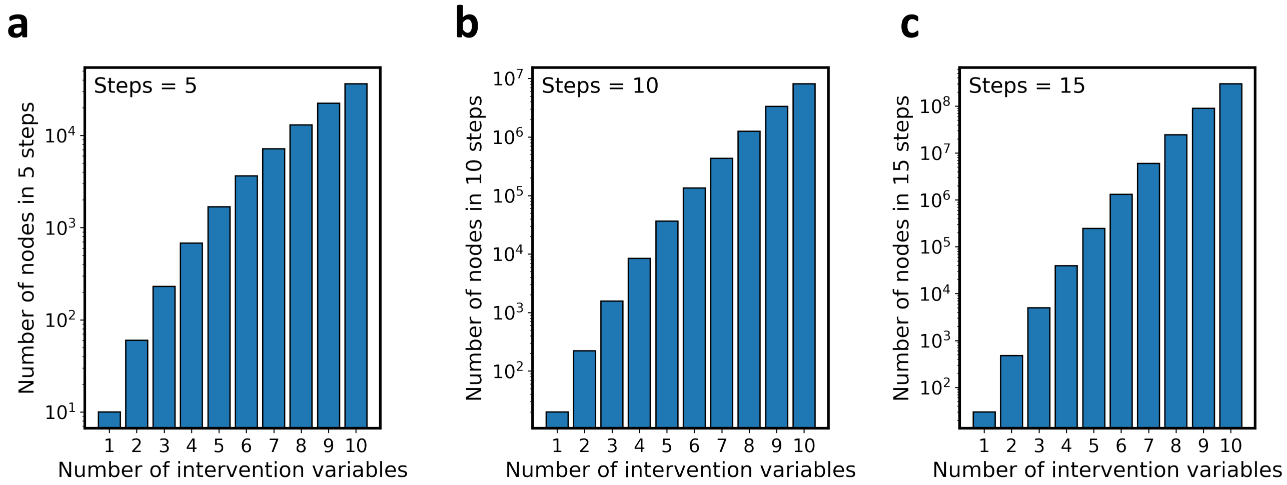

Supplementary Figure 31. Number of nodes within certain number of steps without detours. The number of nodes within 5, 10, or 15 steps are shown. The vertical axis is represented on a logarithmic scale. As the intervention variable increases, the number of nodes increases exponentially, which causes multistep path planning difficult.

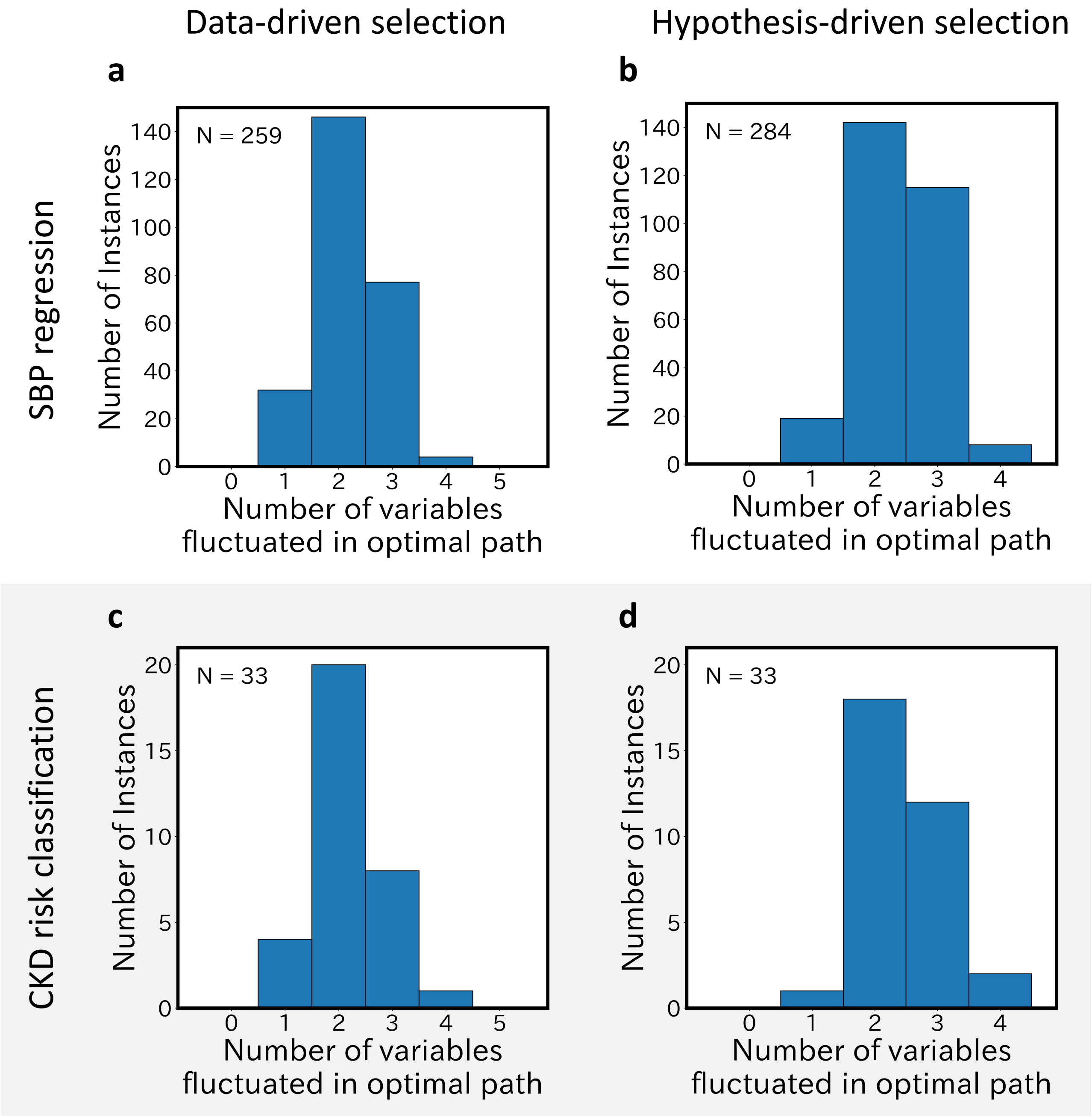

Supplementary Figure 32. Number of intervention variables fluctuated in optimal paths. The number of intervention variables that fluctuated in the optimal path for each instance is shown. **a, b** Data-driven (**a**) or hypothesis-driven (**b**) selected intervention variables on systolic blood pressure (SBP) regression task. **c, d** Data-driven (**c**) or hypothesis-driven (**d**) selected intervention variables on chronic kidney disease (CKD) risk classification task.

a

	Changes from our model
Our model	-
1	$\sigma = \mathrm{RMSE}_{\mathrm{test}}$
2	$\sigma = 2\mathrm{RMSE}_{\mathrm{test}}$
3	$\beta_{1,k} \sim N(0,1)$
4	$\beta_{1,k} \sim N(0,3)$
5	$\boldsymbol{\beta}_{2,k} \sim \mathrm{DoubleExponential}(0, 0.1)$ $\boldsymbol{\beta}_{3,k} \sim \mathrm{DoubleExponential}(0, 0.1)$
6	$\boldsymbol{\beta}_{2,k} \sim \mathrm{DoubleExponential}(0, 5)$ $\boldsymbol{\beta}_{3,k} \sim \mathrm{DoubleExponential}(0, 5)$

	Changes from our model
7	$\boldsymbol{\beta}_{2,k} \sim \mathrm{DoubleExponential}(0, 10)$ $\boldsymbol{\beta}_{3,k} \sim \mathrm{DoubleExponential}(0, 10)$
8	$\boldsymbol{\pi} \sim \mathrm{Dirichlet}(\mathbf{0.5})$
9	$\boldsymbol{\pi} \sim \mathrm{Dirichlet}(\mathbf{2})$
10	$\mathbf{m}_k \sim N(0, \mathbf{I})$
11	$\mathbf{m}_k \sim N(0, 3\mathbf{I})$
12	$\boldsymbol{\Sigma}_k \sim \mathrm{diag}(\mathrm{Cauchy}(0,1))$
13	$\boldsymbol{\Sigma}_k \sim \mathrm{diag}(\mathrm{Cauchy}(0,5))$

b

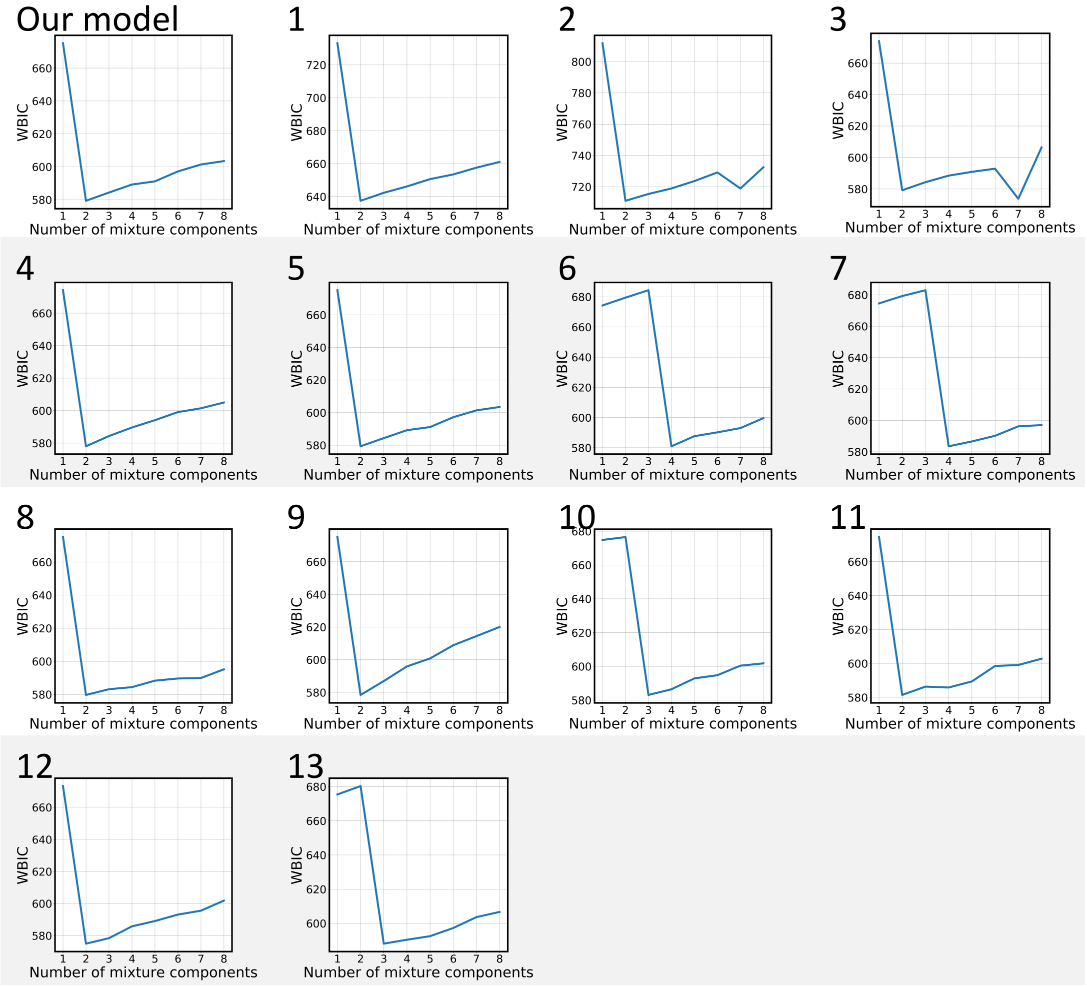

Supplementary Figure 33. Sensitivity analysis of hyperparameters in priors of hierarchical Bayesian model using three-dimensional (3D) synthetic dataset. a List of hyperparameters examined. The hyperparameters are changed from our model setting in the main manuscript. **b** Widely applicable Bayesian information criterion (WBIC) of stochastic surrogate models with 1–8 mixture components.

a

	Changes from our model
Our model	-
1	$\sigma = \mathrm{RMSE}_{\mathrm{test}}$
2	$\sigma = 2\mathrm{RMSE}_{\mathrm{test}}$
3	$\beta_{1,k} \sim N(0, 1)$
4	$\beta_{1,k} \sim N(0, 3)$
5	$\boldsymbol{\beta}_{2,k} \sim \mathrm{DoubleExponential}(0, 0.1)$ $\boldsymbol{\beta}_{3,k} \sim \mathrm{DoubleExponential}(0, 0.1)$
6	$\boldsymbol{\beta}_{2,k} \sim \mathrm{DoubleExponential}(0, 5)$ $\boldsymbol{\beta}_{3,k} \sim \mathrm{DoubleExponential}(0, 5)$

	Changes from our model
7	$\boldsymbol{\beta}_{2,k} \sim \mathrm{DoubleExponential}(0, 10)$ $\boldsymbol{\beta}_{3,k} \sim \mathrm{DoubleExponential}(0, 10)$
8	$\boldsymbol{\pi} \sim \mathrm{Dirichlet}(\mathbf{0.5})$
9	$\boldsymbol{\pi} \sim \mathrm{Dirichlet}(\mathbf{2})$
10	$\mathbf{m}_k \sim N(0, \mathbf{I})$
11	$\mathbf{m}_k \sim N(0, 3\mathbf{I})$
12	$\boldsymbol{\Sigma}_k \sim \mathrm{diag}(\mathrm{Cauchy}(0, 1))$
13	$\boldsymbol{\Sigma}_k \sim \mathrm{diag}(\mathrm{Cauchy}(0, 5))$

b

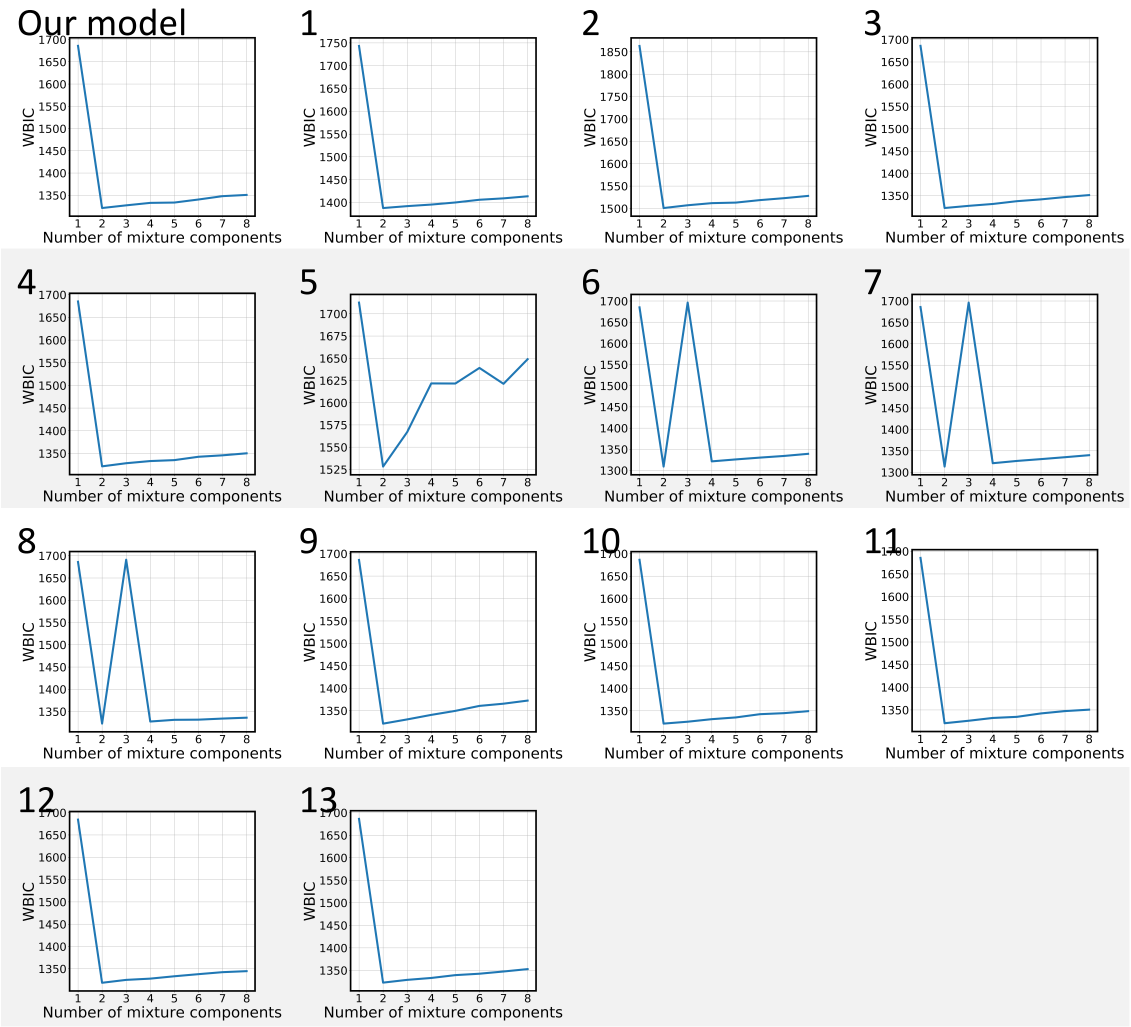

Supplementary Figure 34. Sensitivity analysis of hyperparameters in priors of hierarchical Bayesian model using five-dimensional (5D) synthetic dataset. a List of hyperparameters examined. The hyperparameters are changed from our model setting in the main manuscript. **b** Widely applicable Bayesian information criterion (WBIC) of stochastic surrogate models with 1–8 mixture components.

a

	Changes from our model
Our model	-
1	$\sigma = \mathrm{RMSE}_{\mathrm{test}}$
2	$\sigma = 2\mathrm{RMSE}_{\mathrm{test}}$
3	$\beta_{1,k} \sim N(0,1)$
4	$\beta_{1,k} \sim N(0,3)$
5	$\boldsymbol{\beta}_{2,k} \sim \mathrm{DoubleExponential}(0, 0.1)$ $\boldsymbol{\beta}_{3,k} \sim \mathrm{DoubleExponential}(0, 0.1)$
6	$\boldsymbol{\beta}_{2,k} \sim \mathrm{DoubleExponential}(0, 5)$ $\boldsymbol{\beta}_{3,k} \sim \mathrm{DoubleExponential}(0, 5)$
7	$\boldsymbol{\beta}_{2,k} \sim \mathrm{DoubleExponential}(0, 10)$ $\boldsymbol{\beta}_{3,k} \sim \mathrm{DoubleExponential}(0, 10)$
8	$\boldsymbol{\pi} \sim \mathrm{Dirichlet}(\mathbf{0.5})$
9	$\boldsymbol{\pi} \sim \mathrm{Dirichlet}(\mathbf{2})$
10	$\mathbf{m}_k \sim N(0, \mathbf{I})$
11	$\mathbf{m}_k \sim N(0, 3\mathbf{I})$
12	$\boldsymbol{\Sigma}_k \sim \mathrm{diag}(\mathrm{Cauchy}(0,1))$
13	$\boldsymbol{\Sigma}_k \sim \mathrm{diag}(\mathrm{Cauchy}(0,5))$

b

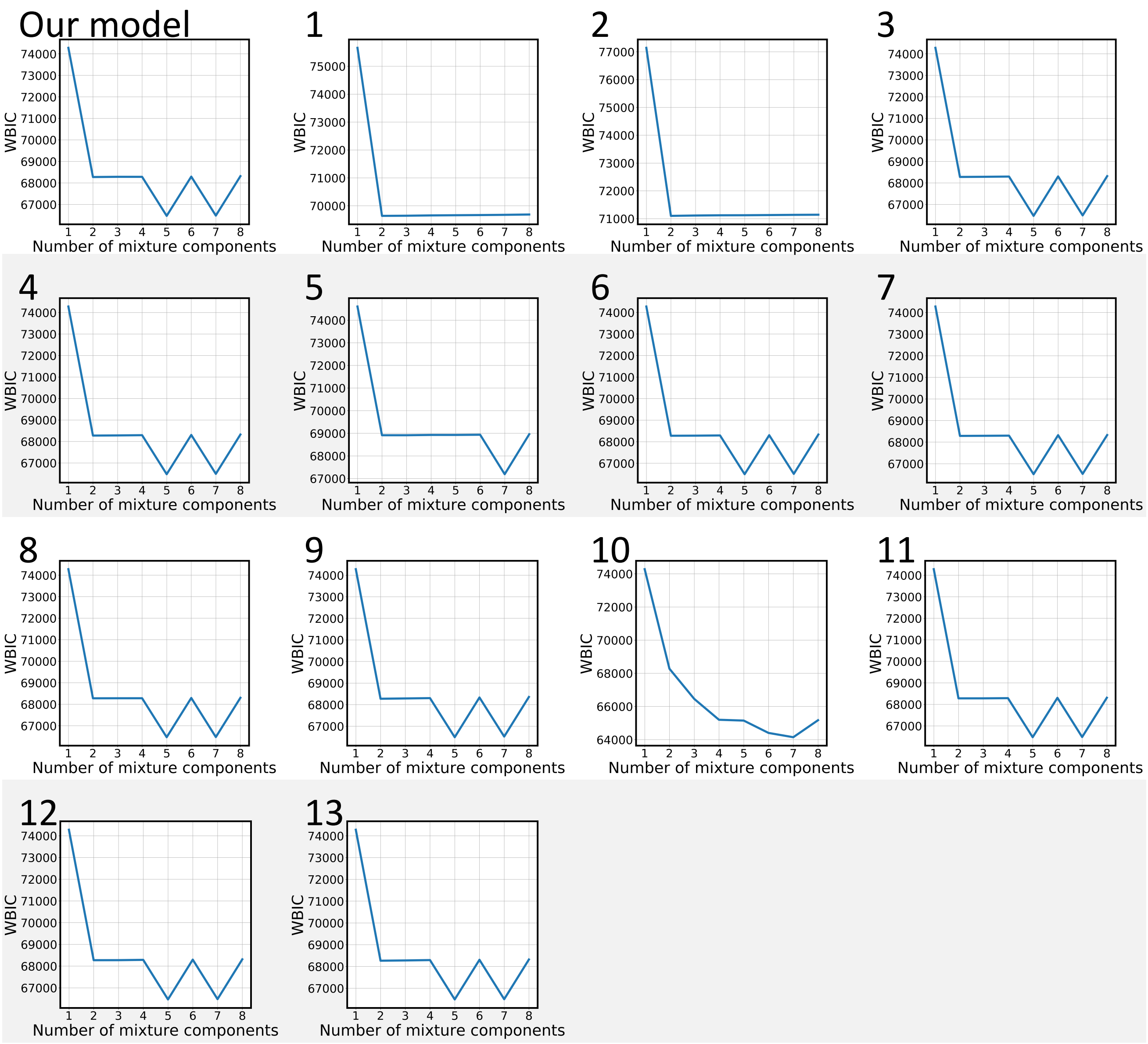

c Instance 1

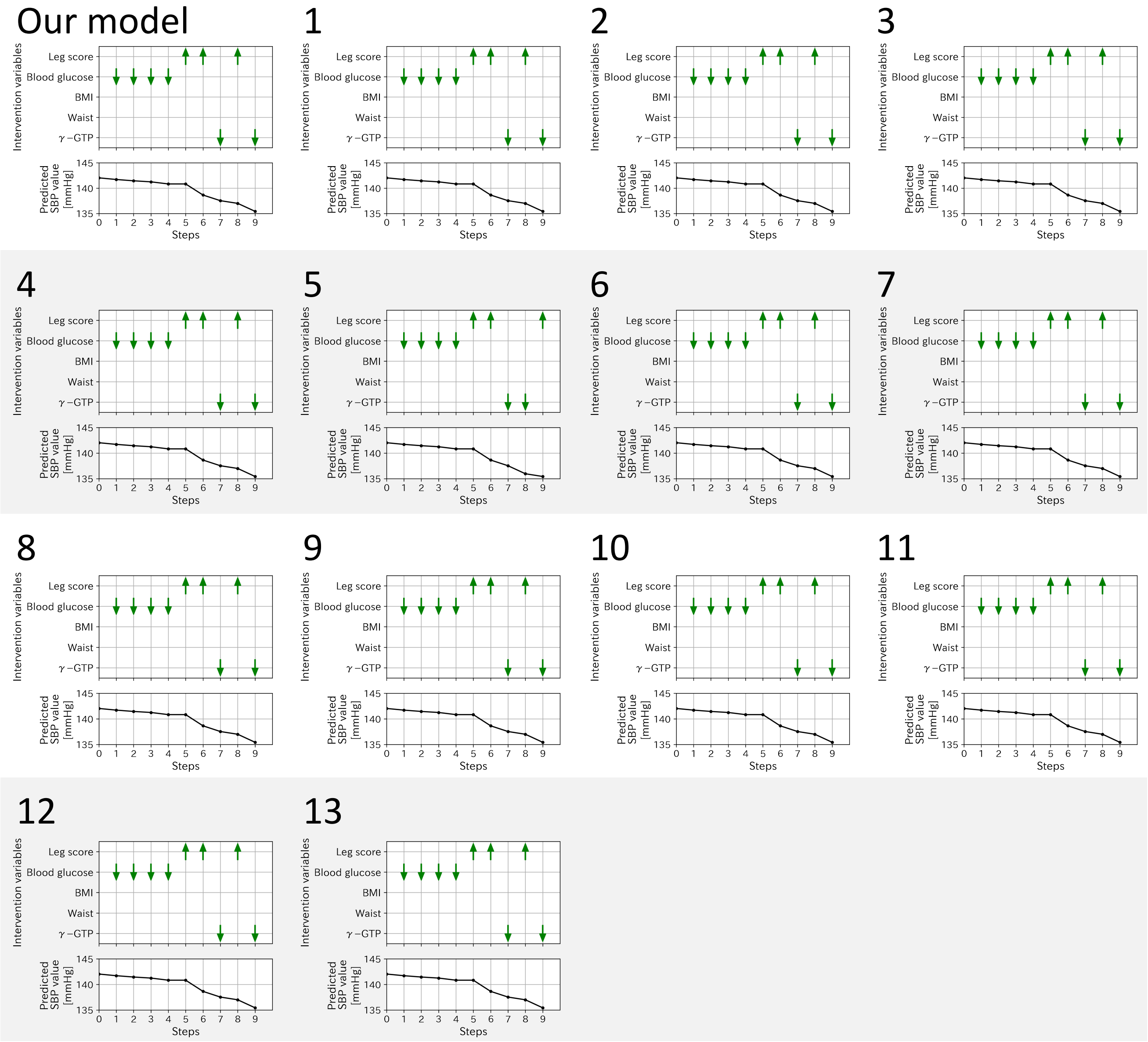

Our model
1
2
3
4
5
6
7
8
9
10
11
12
13
Intervention variables
Leg score
Blood glucose
BMI
Waist
γ−GTP
Predicted SBP value [mmHg]
145
140
135
0 1 2 3 4 5 6 7 8 9
Steps

d Instance 2

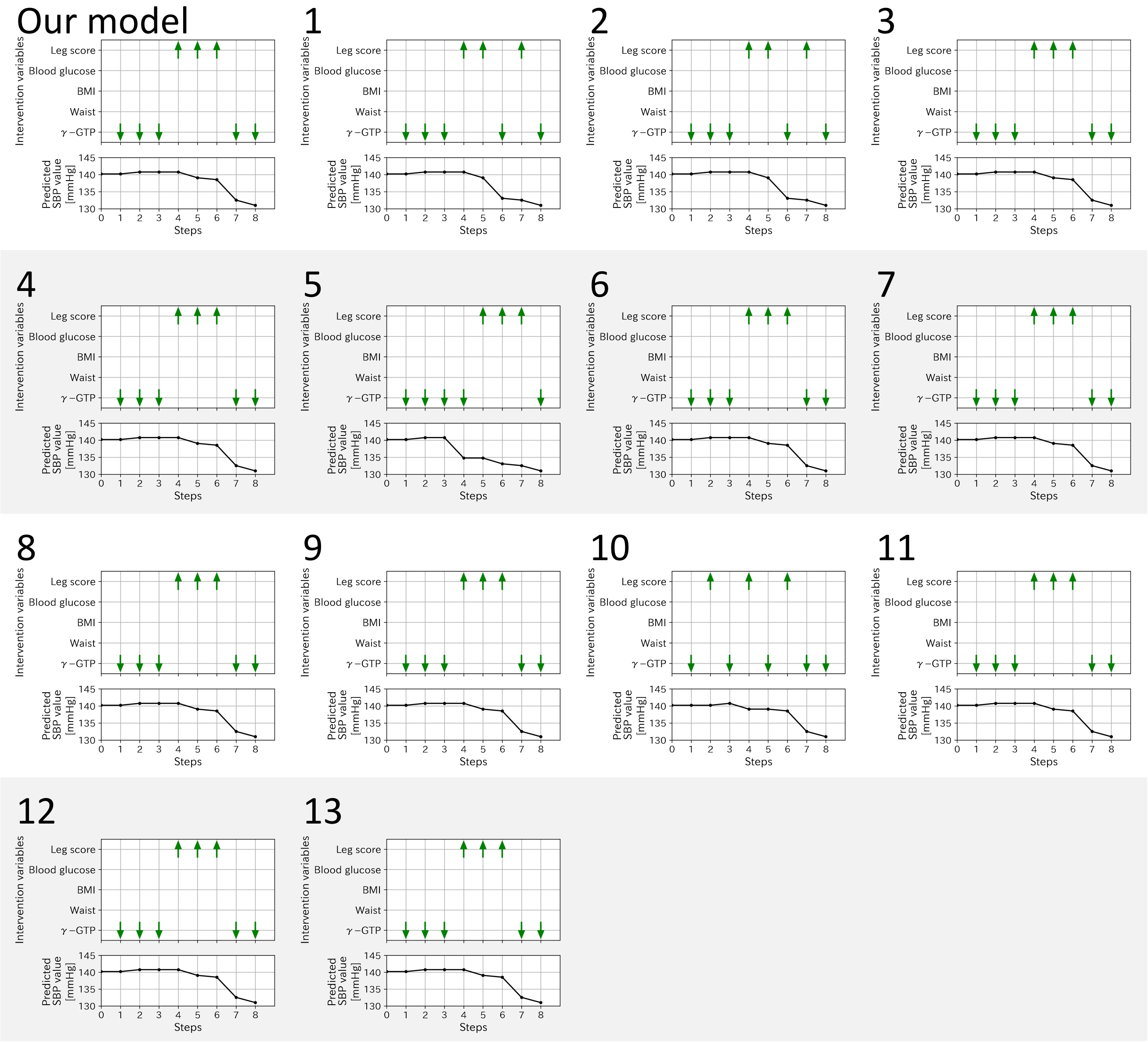

Our model
1
2
3
4
5
6
7
8
9
10
11
12
13
Intervention variables
Leg score
Blood glucose
BMI
Waist
γ−GTP
Predicted SBP value [mmHg]
145
140
135
130
0 1 2 3 4 5 6 7 8
Steps

e Instance 3

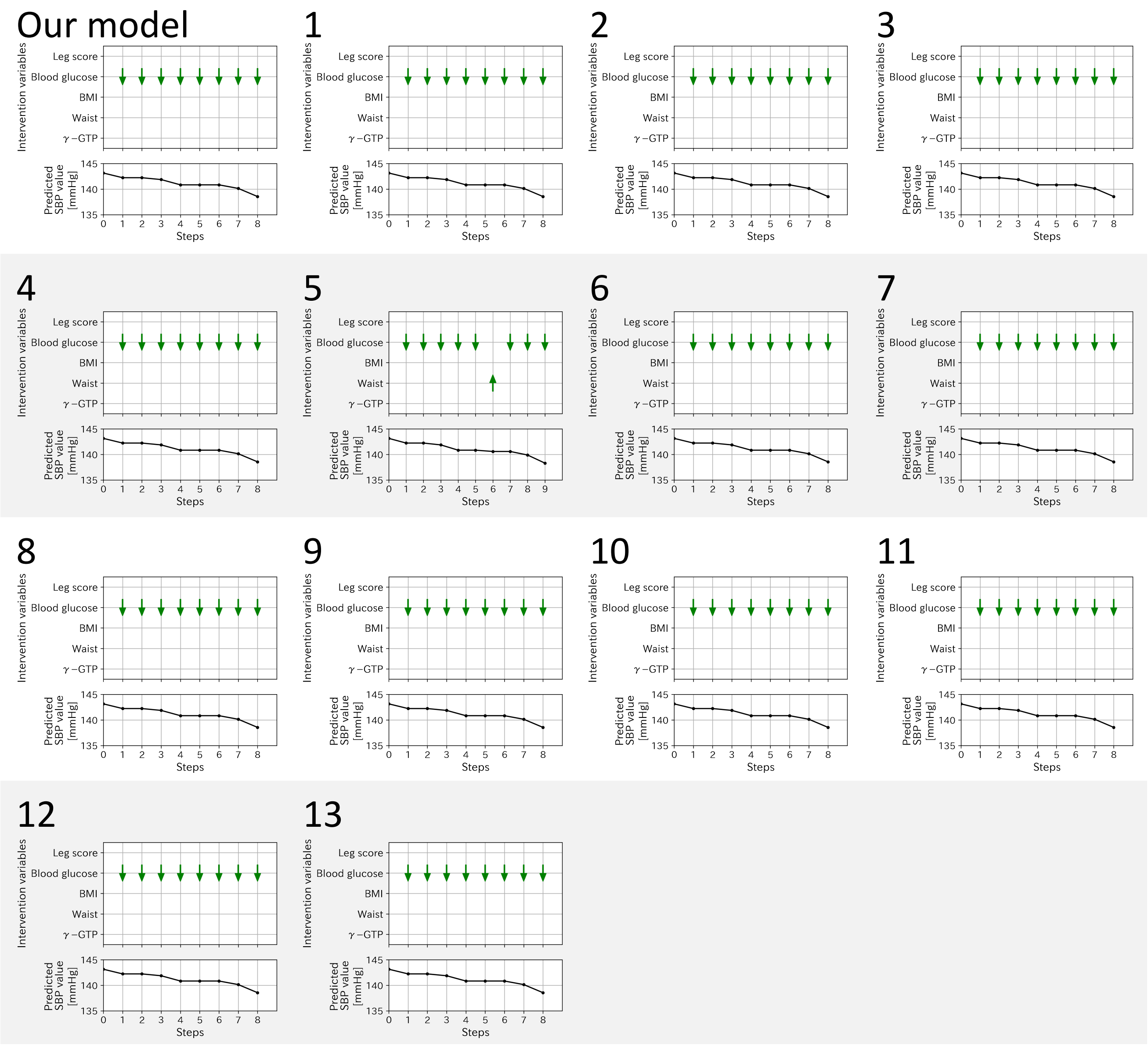

Supplementary Figure 35. Sensitivity analysis of hyperparameters in priors of hierarchical Bayesian model on systolic blood pressure (SBP) regression task. a List of hyperparameters examined. The hyperparameters are changed from our model setting in the main manuscript. **b** Widely applicable Bayesian information criterion (WBIC) of stochastic surrogate models with 1–8 mixture components. **c–e** Paths planned using stochastic surrogate models for each hyperparameter setting. Each subfigure corresponds to the instances described in Fig. 6 in the main text: instance 1 (**c**), instance 2 (**d**), and instance 3 (**e**).

a

	Changes from our model
Our model	-
1	$\beta_{1,k} \sim N(0,1)$
2	$\beta_{1,k} \sim N(0,3)$
3	$\boldsymbol{\beta}_{2,k} \sim \text{DoubleExponential}(0, 0.1)$<br>$\boldsymbol{\beta}_{3,k} \sim \text{DoubleExponential}(0, 0.1)$
4	$\boldsymbol{\beta}_{2,k} \sim \text{DoubleExponential}(0, 5)$<br>$\boldsymbol{\beta}_{3,k} \sim \text{DoubleExponential}(0, 5)$
5	$\boldsymbol{\beta}_{2,k} \sim \text{DoubleExponential}(0, 10)$<br>$\boldsymbol{\beta}_{3,k} \sim \text{DoubleExponential}(0, 10)$

	Changes from our model
6	$\boldsymbol{\pi} \sim \text{Dirichlet}(\mathbf{0.5})$
7	$\boldsymbol{\pi} \sim \text{Dirichlet}(\mathbf{2})$
8	$\mathbf{m}_k \sim N(0, \mathbf{I})$
9	$\mathbf{m}_k \sim N(0, 3\mathbf{I})$
10	$\boldsymbol{\Sigma}_k \sim \text{diag}(\text{Cauchy}(0, 1))$
11	$\boldsymbol{\Sigma}_k \sim \text{diag}(\text{Cauchy}(0, 5))$

b

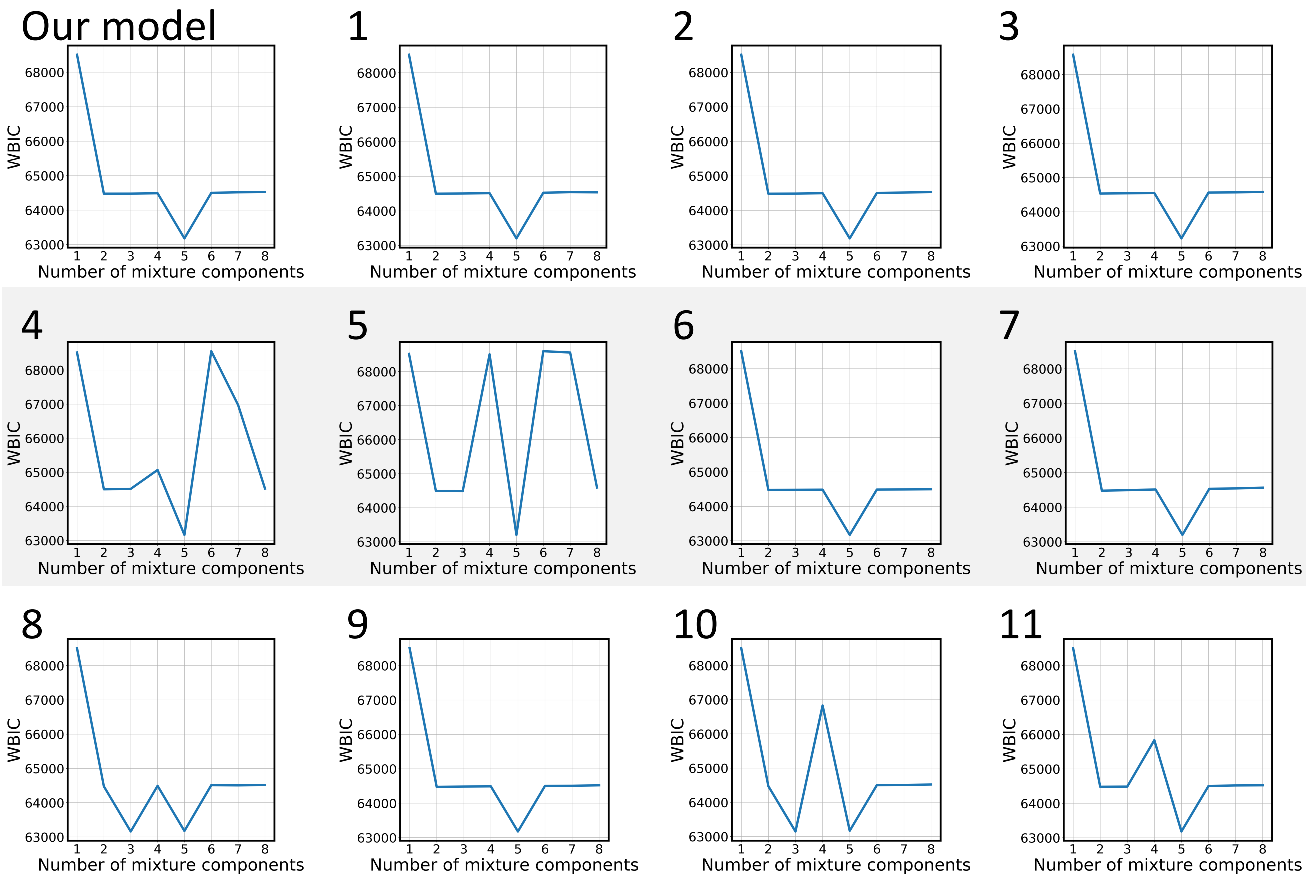

c Instance 4

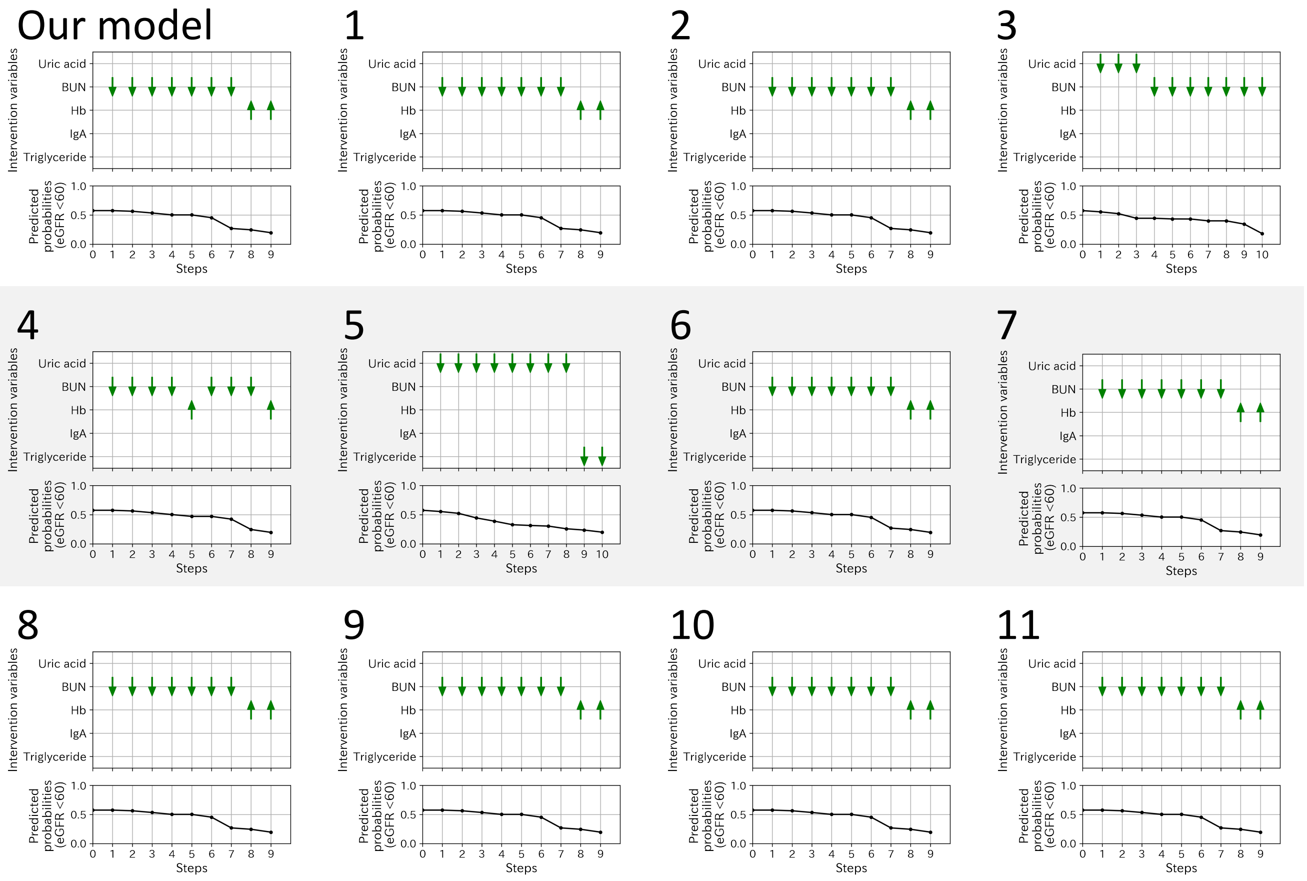
Our model
1
2
3
4
5
6
7
8
9
10
11
Intervention variables
Uric acid
BUN
Hb
IgA
Triglyceride
Predicted probabilities (eGFR <60)
1.0
0.5
0.0
Steps

d Instance 5

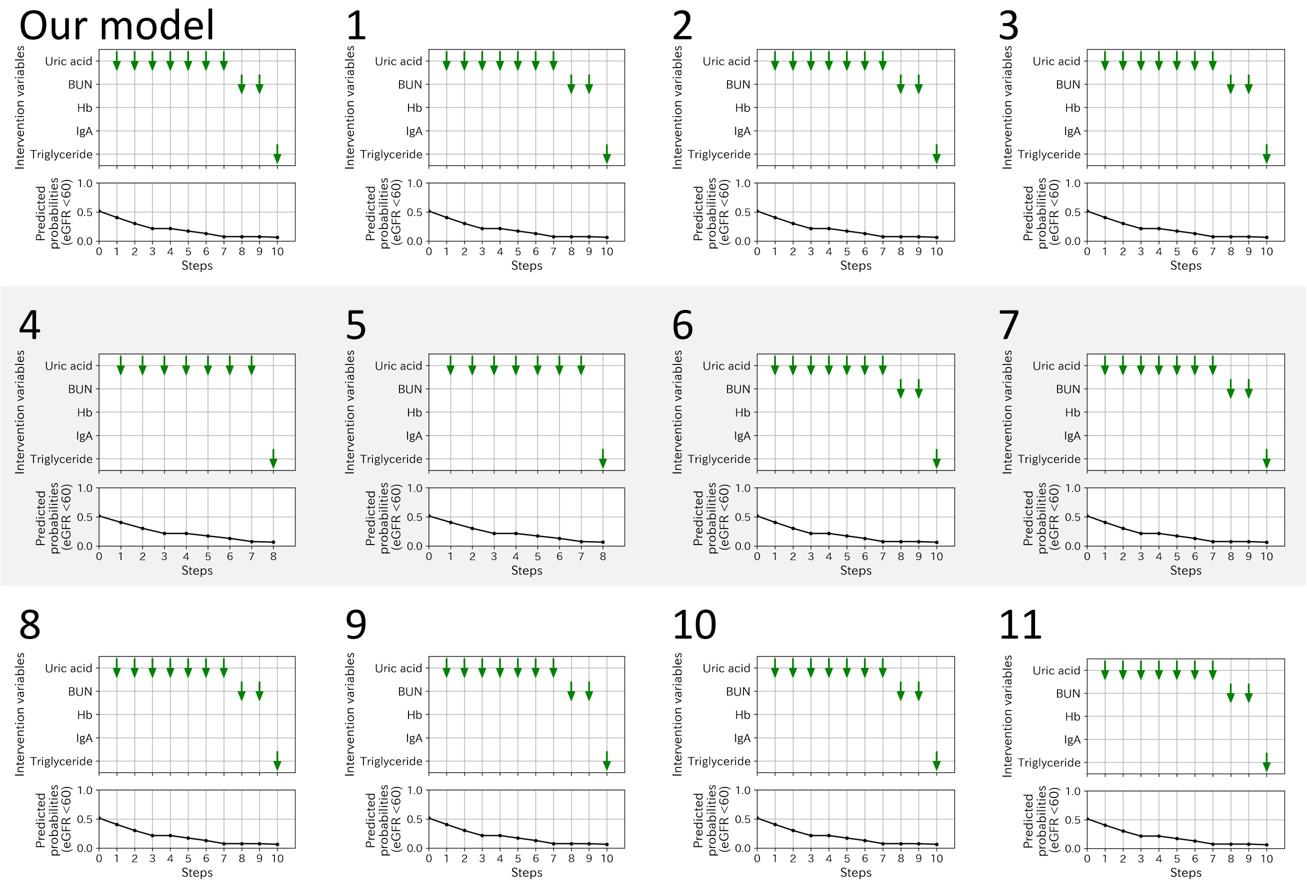
Our model
1
2
3
4
5
6
7
8
9
10
11
Intervention variables
Uric acid
BUN
Hb
IgA
Triglyceride
Predicted probabilities (eGFR <60)
Steps

e Instance 6

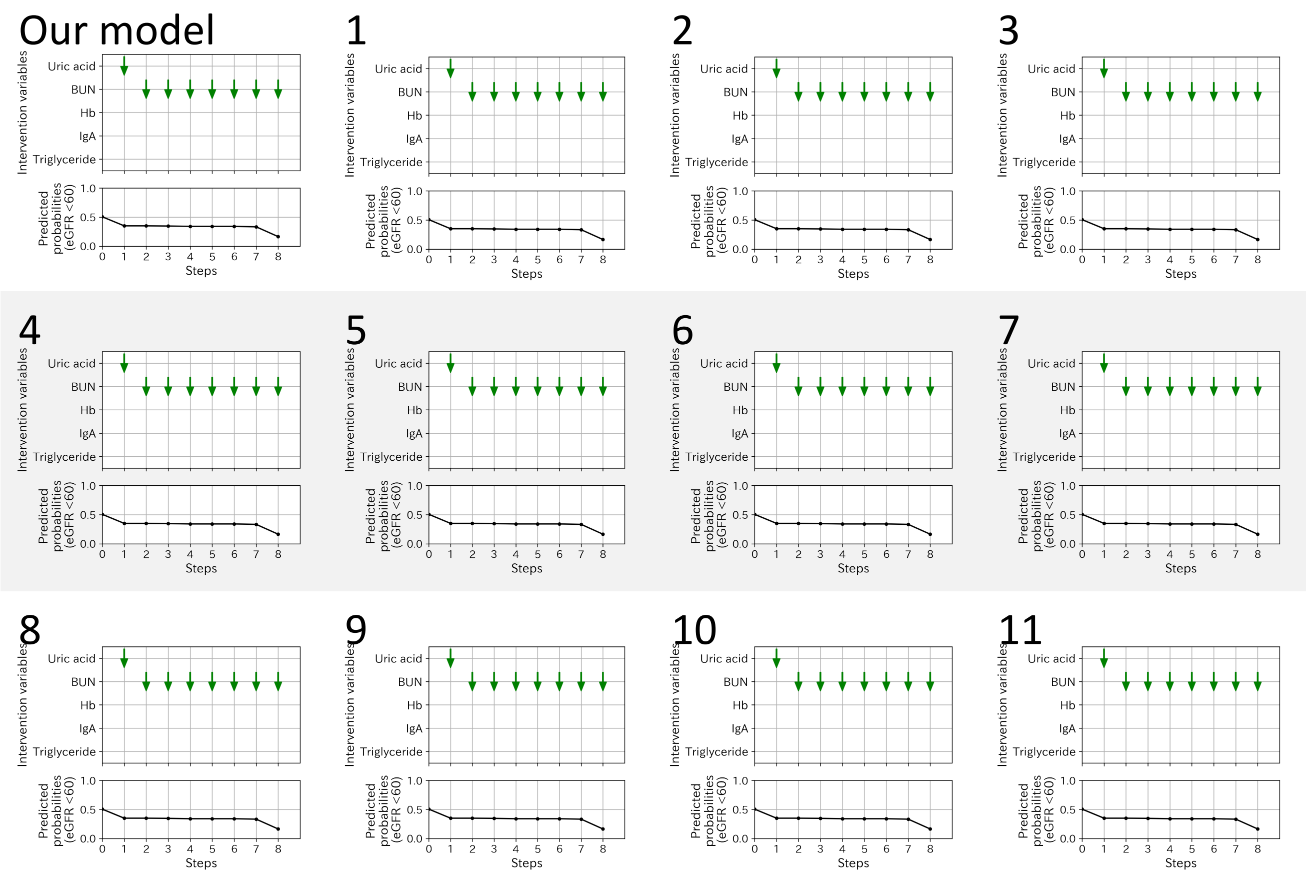

Supplementary Figure 36. Sensitivity analysis of hyperparameters in priors of hierarchical Bayesian model on chronic kidney disease (CKD) risk classification task. a List of hyperparameters examined. The hyperparameters are changed from our model setting in the main manuscript. **b** Widely applicable Bayesian information criterion (WBIC) of stochastic surrogate models with 1–8 mixture components. **c–e** Paths planned using stochastic surrogate models for each hyperparameter setting. Each subfigure corresponds to the instances described in Fig. 8 in the main text: instance 4 (**c**), instance 5 (**d**), and instance 6 (**e**).

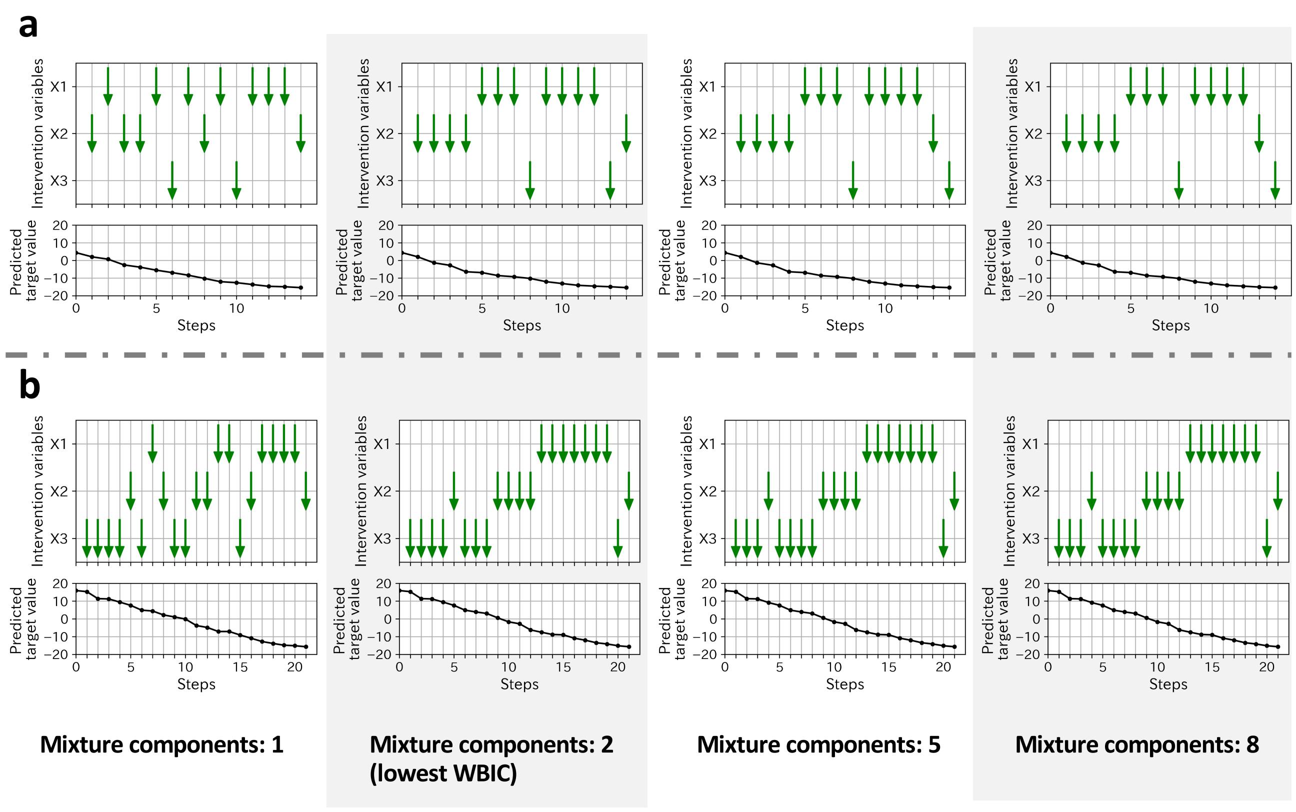

Supplementary Figure 37. Sensitivity analysis of path planning using hierarchical Bayesian model with different number of mixture components on three-dimensional (3D) synthetic dataset. The optimal paths for improving the response variable predicted by the XGBoost model were planned using hierarchical Bayesian models with different number of mixture components. Displayed instances are the same as in Fig. 4: instance A (**a**) and instance B (**b**).

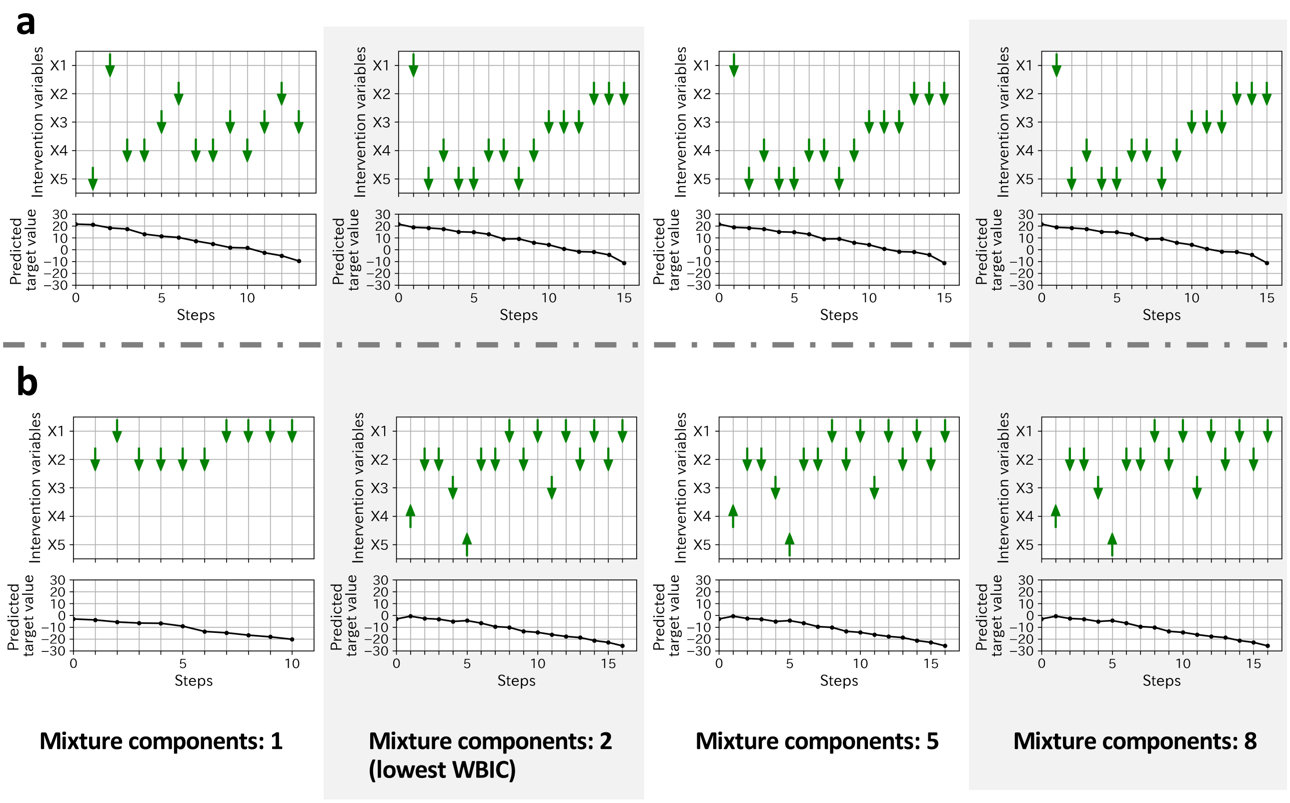

Supplementary Figure 38. Sensitivity analysis of path planning using hierarchical Bayesian model with different number of mixture components on five-dimensional (5D) synthetic dataset. The optimal paths for improving the response variable predicted by the XGBoost model were planned using hierarchical Bayesian models with different number of mixture components. Displayed instances are the same as in Supplementary Fig. 13: instance C (**a**) and instance D (**b**).

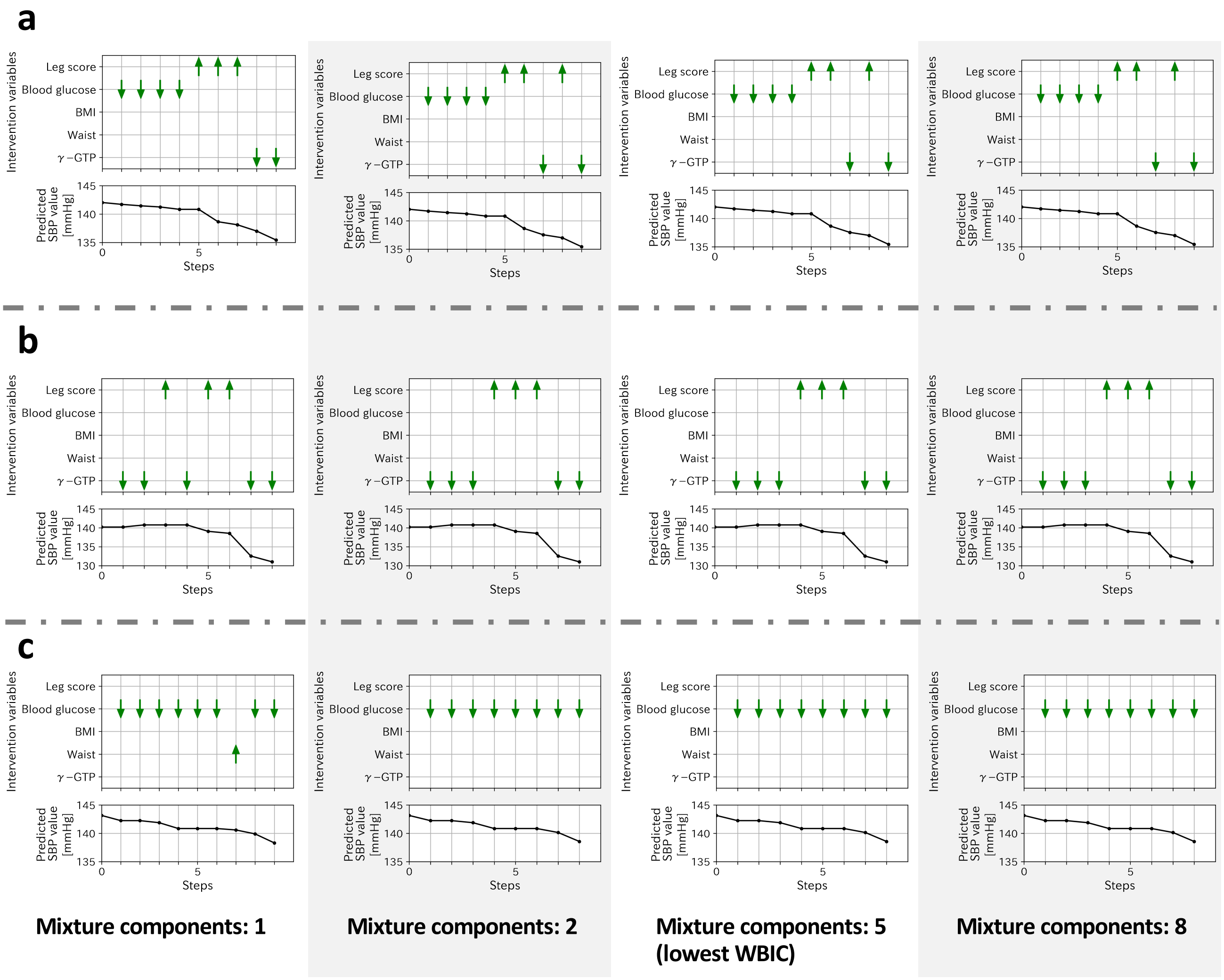

Supplementary Figure 39. Sensitivity analysis of path planning using hierarchical Bayesian model with different number of mixture components on systolic blood pressure (SBP) regression task. The optimal paths for improving the response variable predicted by the XGBoost model were planned using hierarchical Bayesian models with different number of mixture components. Displayed instances are the same as in Fig. 6: instance 1 (**a**), instance 2 (**b**), and instance 3 (**c**).

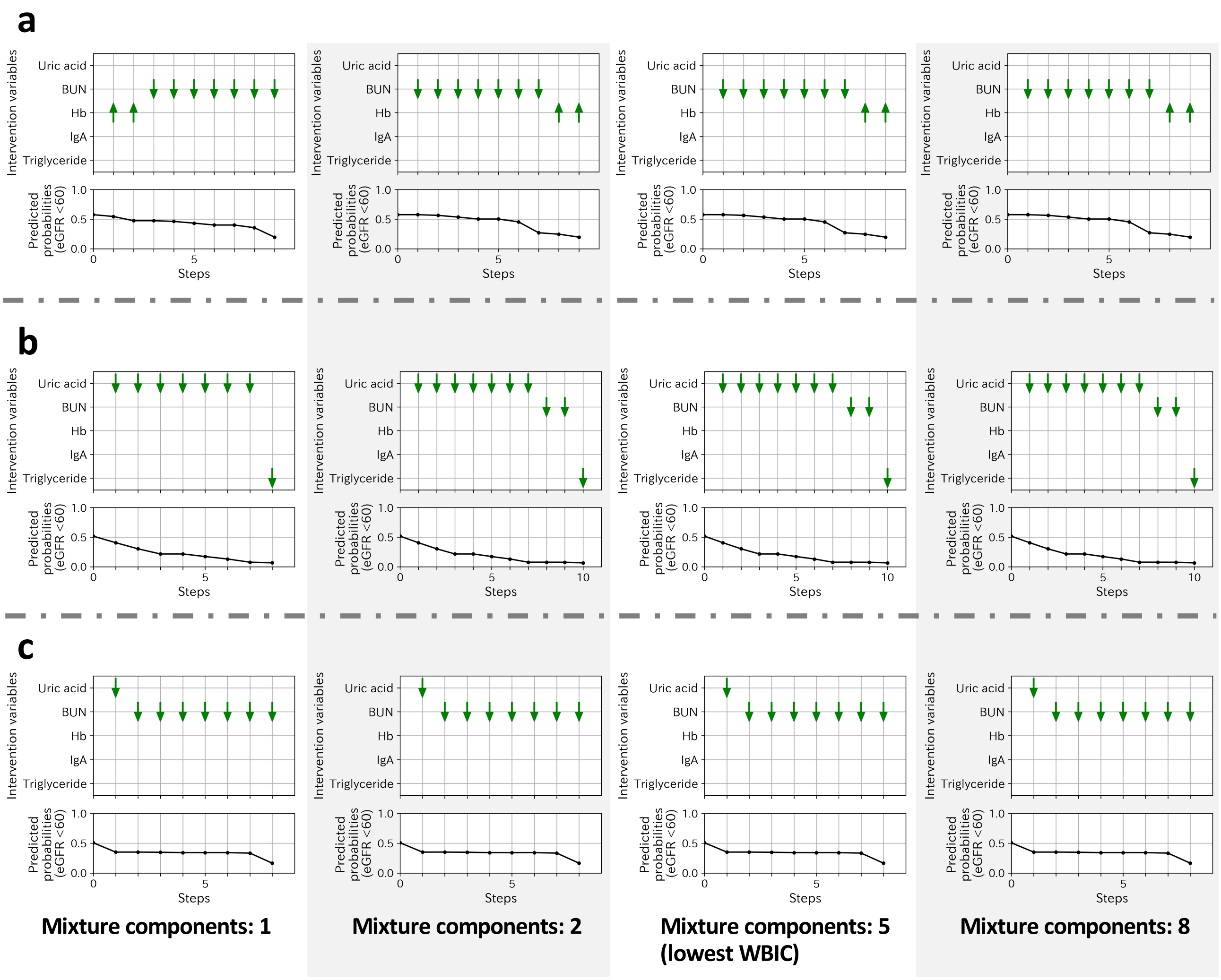

Supplementary Figure 40. Sensitivity analysis of path planning using hierarchical Bayesian model with different number of mixture components on chronic kidney disease (CKD) risk classification task. The optimal paths for improving the response variable predicted by the XGBoost model were planned using hierarchical Bayesian models with different number of mixture components. Displayed instances are the same as in Fig. 8: instance 4 (**a**), instance 5 (**b**), and instance 6 (**c**).

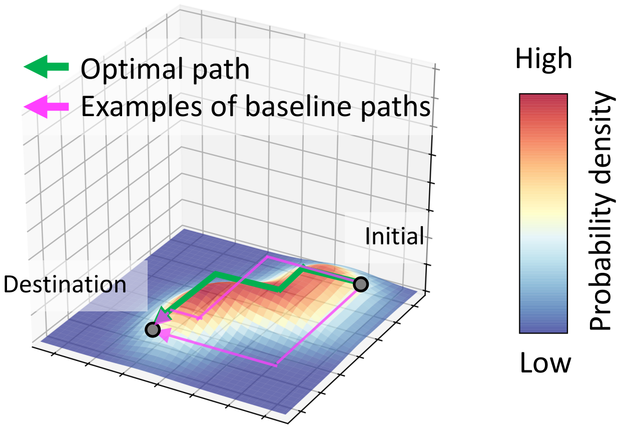

Supplementary Figure 41. Schematic explanation about actionability score. A baseline path is defined as a path that connects the initial node and destination node of the optimal path presented by the framework with a random minimum step, that is, without detour. Actionability score is defined as the logarithm of the ratio of the optimal path-actionability to the baseline path-actionability. Due to the large number of baseline paths, we used the geometric mean of the actionability of ten random baseline paths to calculate the score. The higher the actionability score, the optimal path is constructed through nodes with higher probabilities than the baseline path.

$$\begin{bmatrix} x_1 \\ \vdots \\ x_5 \end{bmatrix} \sim \mathcal{N}(\boldsymbol{\mu}_k, \boldsymbol{\Sigma}_k) \qquad k \in \{1, \cdots, 5\}$$

$$\boldsymbol{\mu}_k = \begin{bmatrix} \mu_1 \\ \vdots \\ \mu_5 \end{bmatrix} \qquad \mu_i = \begin{cases} 5 \; (i < k) \\ 0 \; (i = k) \\ -5 \; (i > k) \end{cases}$$

$$\boldsymbol{\Sigma}_k = \begin{bmatrix} \sigma_{11} & \cdots & \sigma_{15} \\ \vdots & \ddots & \vdots \\ \sigma_{51} & \cdots & \sigma_{55} \end{bmatrix} \qquad \sigma_{ij} = \begin{cases} 5 \; (i = j = k) \\ 1 \; (i = j \neq k) \\ \mathcal{N}(0,1) \; (i < j) \\ \sigma_{ji} \; (i > j) \end{cases}$$

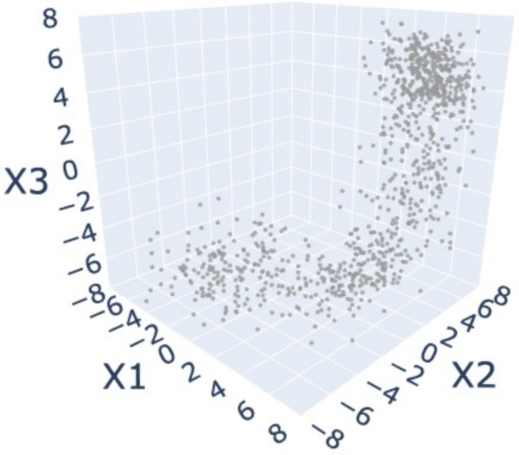

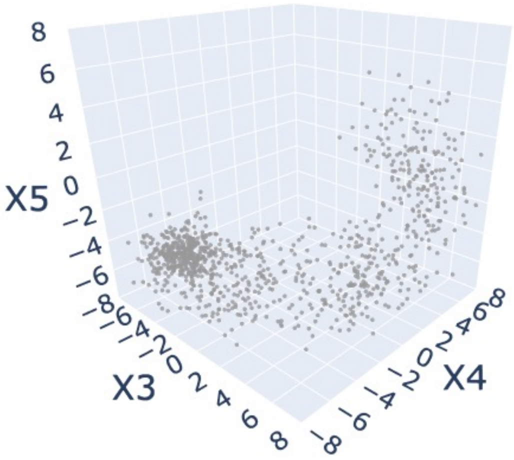

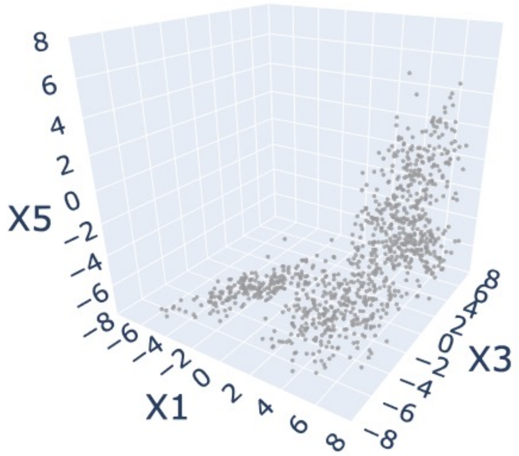

Supplementary Figure 42. Generation of five-dimensional (5D) synthetic dataset. The five 5D normal distributions generated 200 data points that consisted of x_i ($i \in \{1, \ldots, 5\}$). Subsequently, a response variable was set to the sum of x_i with Gaussian noise ($\sigma = 2$). The synthetic dataset consisted of a total of 1,000 data points with explanatory variables (X1, …, X5) and a response variable.

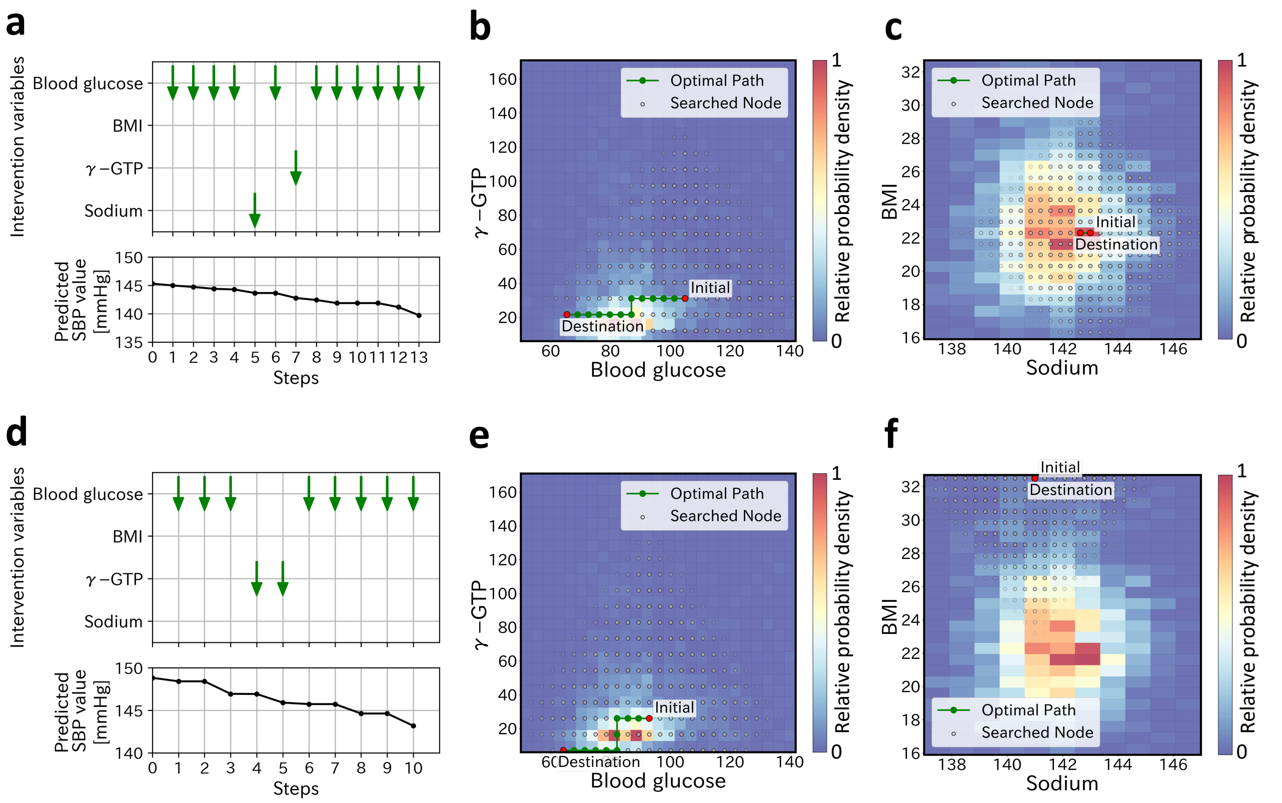

Supplementary Figure 43. Examples of personal actionable paths for treatment with intervention variables based on hypothesis-driven selection in systolic blood pressure (SBP) regression task. The optimal paths for improving the response variable predicted by the ML model are represented for two examples: instance 11 (**a–c**) and instance 12 (**d–f**). **a, d** The orders of changes in the explanatory variables in the optimal path and the accompanying changes in the predicted values. In the transition steps, the upward or downward arrow represents a unit increase or decrease in the explanatory variable, respectively. **b, c, e, f** 2D plots of the path. In the heatmaps, the probability density of the actual data, normalized by the panel with the maximum number of data, is expressed.

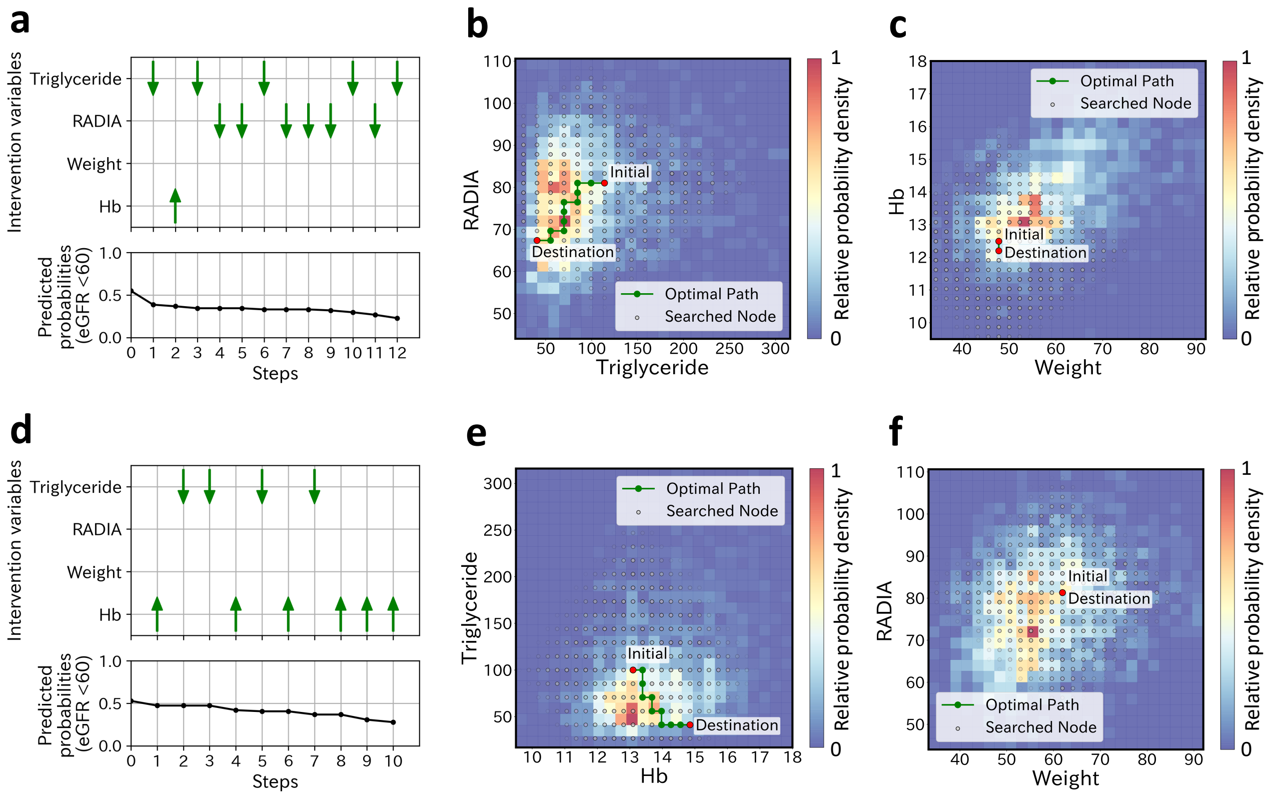

Supplementary Figure 44. Examples of personal actionable paths for treatment with intervention variables based on hypothesis-driven selection in chronic kidney disease (CKD) risk classification task. The optimal paths for improving the response variable predicted by the ML model are represented for two examples: instance 13 (**a**–**c**) and instance 14 (**d**–**f**). **a, d** The orders of changes in the explanatory variables in the optimal path and the accompanying changes in the predicted values. In the transition steps, the upward or downward arrow represents a unit increase or decrease in the explanatory variable, respectively. **b, c, e, f** 2D plots of the path. In the heatmaps, the probability density of the actual data, normalized by the panel with the maximum number of data, is expressed.

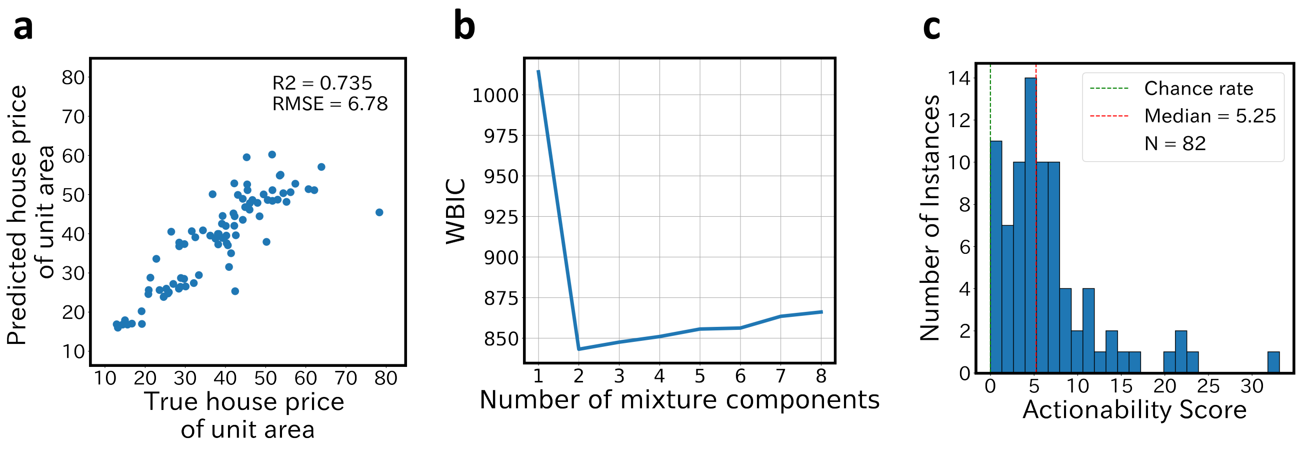

Supplementary Figure 45. Results of proposed framework on real estate dataset. a Plot for prediction vs. true response variable. **b** WBIC values of the stochastic surrogate models with 1–8 mixture components. **c** Histogram of actionability scores at different instances. An actionability score of zero indicates that the actionability of the optimal path is equivalent to that of the baseline path.

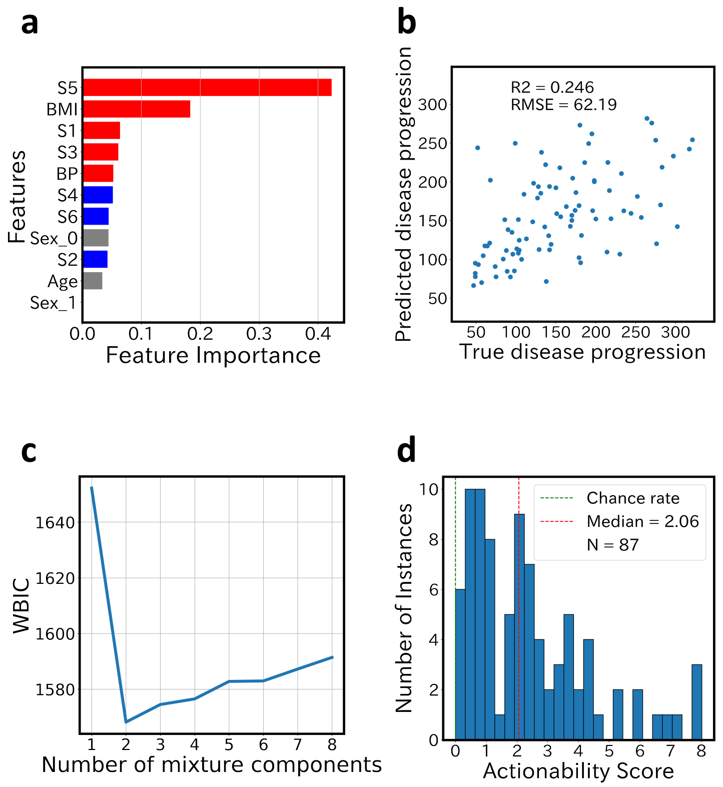

Supplementary Figure 46. Results of proposed framework on diabetes progression dataset. a Feature importance of the regression model. The color of each bar represents the following: red: intervention variables in path planning, gray: variables which are difficult to be intervened, and blue: other variables. Details of features are described in Supplementary Table 7. **b** Plot for prediction vs. true response variable. **c** WBIC values of stochastic surrogate models with 1–8 mixture components. **d** Histogram of actionability scores for each instance. An actionability score of zero indicates that the actionability of the optimal path is equivalent to that of the baseline path.

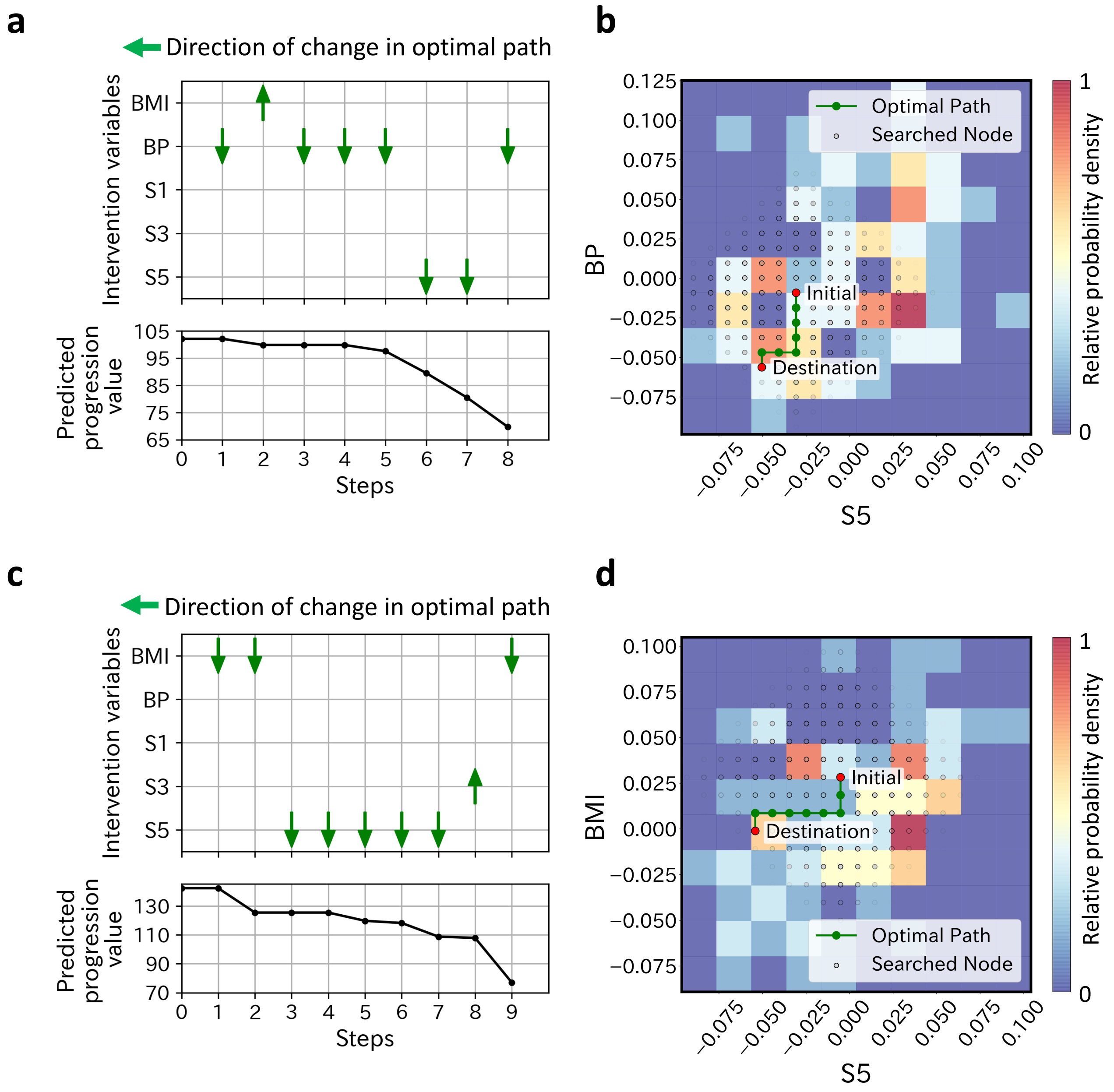

Supplementary Figure 47. Examples of personal actionable paths for treatment on diabetes progression dataset. The optimal paths needed for the improvement of the response variables predicted by the machine learning model are represented for randomly selected two examples: instance D1 (**a, b**) and instance D2 (**c, d**). **a, c** The orders of changes in the explanatory variables in the optimal path and the accompanying changes in the predicted values. In the transition steps, the upward or downward arrow represents a unit increase or decrease in the explanatory variable, respectively. **b, d** Two-dimensional (2D) plots of the path. The 2D plots are shown regarding the two influential variables: S5 and average blood pressure (BP) (**b**), and S5 and body mass index (BMI) (**d**). In the heatmaps, the probability density of the actual data, normalized by the panel with the maximum number of data, is expressed.

Supplementary Tables

Supplementary Table 1. The number of instances.

Task	Training	Test	Surrogate modeling
Regression task on SBP	10,260	2,543	2,222
Classification task on SBP (Hypertension risk)	10,308	2,495	2,185
Regression task on eGFR	10,233	2,563	2,230
Classification task on eGFR (CKD risk)	10,194	2,602	2,255

Supplementary Table 2. Correspondence between clinical guideline–recommended treatments for improving blood pressure and variables used in our framework.

Guideline recommended treatments	Examples of corresponding variables
Salt reduction	Serum sodium
Exercise	BMI<br>Waist<br>Leg score<br>Blood glucose
Alcohol restriction	γ -GTP
Alleviation of obesity	BMI<br>Waist
Nutrients and dietary patterns	γ -GTP<br>Blood glucose

BMI: body mass index, γ-GTP: gamma-glutamyltransferase.

Supplementary Table 3. Clinicians' assessments on utility of health-improvement paths.

	Practical		Informative	
	**Optimal path**	**Random path**	**Optimal path**	**Random path**
**Cardiologists**	**0.20 ± 0.21**<br>[0, 5, 3, 2, 0]	**0.22 ± 0.18**<br>[1, 4, 2, 4, 0]	**0.42 ± 0.30**<br>[3, 8, 6, 4, 0]	**0.38 ± 0.33**<br>[1, 7, 4, 7, 0]
**Nephrologists**	**0.30 ± 0.29**<br>[1, 0, 2, 7, 5]	**0.26 ± 0.23**<br>[3, 0, 3, 1, 6]	**0.52 ± 0.26**<br>[2, 4, 6, 9, 5]	**0.46 ± 0.19**<br>[4, 2, 7, 6, 4]

Ratio of paths clinicians evaluated as practical or informative (Mean ± standard deviation). The numbers in brackets indicate the instances each clinician assessed as practical or informative in ten instances.

Supplementary Table 4. Correspondence between clinical guideline–recommended treatments for patients with lower renal functions and variables used in our framework.

Guideline recommended treatments	Examples of corresponding variables
Nutrients and dietary patterns	Weight<br>BMR score<br>Right arm X 50kHz<br>Triglyceride
Lowering blood pressure<br>(Salt reduction, anti-hypertensive drugs)	RADIA
Iron supplementation and other erythropoiesis-stimulating agents	Hb<br>Erythrocyte count
Lipid-lowering treatment	Triglyceride
Alleviation of obesity<br>Regular exercise	Weight<br>BMR score<br>Right arm X 50kHz
Lowering uric acid<br>(Hyperuricemia drugs)	Uric acid

BMR score: basal metabolic rate score, RADIA: right ankle diastolic blood pressure, Hb: hemoglobin.

Supplementary Table 5. Classification model scores on chronic kidney disease (CKD) risk.

Model	AUC	TP	FN	FP	TN
XGBoost	0.844	35	171	19	2,377
Random Forest	0.833	9	197	4	2,392
Support vector machine	0.790	43	163	43	2,353

AUC: area under the curve, TP: true positive, FN: false negative, FP: false positive, TN: true negative.

Supplementary Table 6. Classification model scores on hypertension risk.

Model	AUC	TP	FN	FP	TN
XGBoost	0.772	127	515	98	1,755
Random Forest	0.771	72	570	59	1,794
Support vector machine	0.733	104	538	81	1,772

AUC: area under the curve, TP: true positive, FN: false negative, FP: false positive, TN: true negative.

Supplementary Table 7. Description of the diabetes progression dataset features.

Feature	Description
Response variable	
Disease progression	A quantitative measure of disease progression one year after baseline
Explanatory variables	
Age	Age in years
Sex	
BMI	Body mass index
BP	Average blood pressure
S1	T-cells (a type of white blood cells)
S2	Low-density lipoproteins
S3	High-density lipoproteins
S4	Thyroid stimulating hormone
S5	Lamotrigine
S6	Blood sugar level
