# Supplementary material for: Health improvement framework for actionable treatment planning using a surrogate Bayesian model
Source: Nat Commun. 2021 May 25;12:3088. doi: 10.1038/s41467-021-23319-1 (PMC8149666; doi:10.1038/s41467-021-23319-1)
Supplement: Supplementary file 2 — Description of Additional Supplementary Files [file 41467_2021_23319_MOESM2_ESM.pdf]

Description of Additional Supplementary File

Supplementary Data 1

Description: The items on the Iwaki Health Promotion Project (IHPP) dataset.
