# Supplementary material for: Health improvement framework for actionable treatment planning using a surrogate Bayesian model
Source: Nat Commun. 2021 May 25;12:3088. doi: 10.1038/s41467-021-23319-1 (PMC8149666; doi:10.1038/s41467-021-23319-1)
Supplement: Supplementary file 4 — Reporting Summary [file 41467_2021_23319_MOESM4_ESM.pdf]

nature research

Corresponding author(s): Yasushi Okuno

Last updated by author(s): Apr 22, 2021

Reporting Summary

Nature Research wishes to improve the reproducibility of the work that we publish. This form provides structure for consistency and transparency in reporting. For further information on Nature Research policies, see our Editorial Policies and the Editorial Policy Checklist.

Statistics

For all statistical analyses, confirm that the following items are present in the figure legend, table legend, main text, or Methods section.

n/a	Confirmed	
☐	☒	The exact sample size (n) for each experimental group/condition, given as a discrete number and unit of measurement
☐	☒	A statement on whether measurements were taken from distinct samples or whether the same sample was measured repeatedly
☐	☒	The statistical test(s) used AND whether they are one- or two-sided<br>*Only common tests should be described solely by name; describe more complex techniques in the Methods section.*
☐	☒	A description of all covariates tested
☒	☐	A description of any assumptions or corrections, such as tests of normality and adjustment for multiple comparisons
☐	☒	A full description of the statistical parameters including central tendency (e.g. means) or other basic estimates (e.g. regression coefficient) AND variation (e.g. standard deviation) or associated estimates of uncertainty (e.g. confidence intervals)
☐	☒	For null hypothesis testing, the test statistic (e.g. F, t, r) with confidence intervals, effect sizes, degrees of freedom and P value noted<br>*Give P values as exact values whenever suitable.*
☐	☒	For Bayesian analysis, information on the choice of priors and Markov chain Monte Carlo settings
☐	☒	For hierarchical and complex designs, identification of the appropriate level for tests and full reporting of outcomes
☒	☐	Estimates of effect sizes (e.g. Cohen's d, Pearson's r), indicating how they were calculated

Our web collection on statistics for biologists contains articles on many of the points above.

Software and code

Policy information about availability of computer code

Data collection	No software was used.
Data analysis	We used publicly available software for the data analysis (Python 3.7.3, pandas 0.25.3, numpy 1.16.0, scipy 1.3.2, xgboost 0.82, scikit-learn 0.21.2, matplotlib 3.1.1, and pystan 2.19.1.1 [package for performing Markov chain Monte Carlo algorithm]). We also used custom script to perform hierarchical Bayesian modeling and path planning. We provided these scripts at https://github.com/clinfo/actionable_path_planning.

For manuscripts utilizing custom algorithms or software that are central to the research but not yet described in published literature, software must be made available to editors and reviewers. We strongly encourage code deposition in a community repository (e.g. GitHub). See the Nature Research guidelines for submitting code & software for further information.

Data

Policy information about availability of data

All manuscripts must include a data availability statement. This statement should provide the following information, where applicable:

- Accession codes, unique identifiers, or web links for publicly available datasets
- A list of figures that have associated raw data
- A description of any restrictions on data availability

The synthetic datasets can be generated from the code in the repository provided in the Code availability section. The datasets used in the supplementary information are open available on UCI Machine Learning Repository at http://archive.ics.uci.edu/ml and Trevor Hastie's Software page at https://web.stanford.edu/~hastie/Papers/LARS/. The health checkup data used in this study were collected in the Iwaki Health Promotion Project (IHPP) and transferred to a secure data center with restricted access controls in a de-identified format. The de-identified dataset may be available from Hirosaki University School of Medicine, subjective to individual institution's and IHPP's data governance and ethical approval, and the national law and regulations. All other data in this study are included in this article or are available from the corresponding author upon reasonable request.

Field-specific reporting

Please select the one below that is the best fit for your research. If you are not sure, read the appropriate sections before making your selection.

☒ Life sciences ☐ Behavioural & social sciences ☐ Ecological, evolutionary & environmental sciences

For a reference copy of the document with all sections, see nature.com/documents/nr-reporting-summary-flat.pdf

Life sciences study design

All studies must disclose on these points even when the disclosure is negative.

Sample size	Health checkup data used in this study was obtained from the Iwaki Health Promotion Project (IHPP), which is a retrospective, observational study for residents of the Iwaki district, Hirosaki City, Aomori Prefecture, Japan. The number of unique participants was 3,132, and the IHPP dataset consisted of 12,803 instances. No sample size calculation was performed prior to this study because of the nature of observational study data. However, it was confirmed that the framework worked well with at least 600 data points in the synthetic dataset experiment in the main manuscript. The IHPP dataset exceeded this requirement by two orders of magnitude larger.
Data exclusions	Exclusion criteria were pre-established. We excluded the instances for which the response variable was missing before building each predictive model because response variable values are required to build a predictive model in supervised learning.
Replication	IHPP is an observational study. Replication is not applicable.
Randomization	Sample allocation into training and test datasets are random.
Blinding	In the clinician assessment, the clinicians evaluated the framework-proposed path and random paths in a blinded manner.

Reporting for specific materials, systems and methods

We require information from authors about some types of materials, experimental systems and methods used in many studies. Here, indicate whether each material, system or method listed is relevant to your study. If you are not sure if a list item applies to your research, read the appropriate section before selecting a response.

Materials & experimental systems

n/a	Involved in the study	
☒	☐	Antibodies
☒	☐	Eukaryotic cell lines
☒	☐	Palaeontology and archaeology
☒	☐	Animals and other organisms
☐	☒	Human research participants
☒	☐	Clinical data
☒	☐	Dual use research of concern

Methods

n/a	Involved in the study	
☒	☐	ChIP-seq
☒	☐	Flow cytometry
☒	☐	MRI-based neuroimaging

Human research participants

Policy information about studies involving human research participants

Population characteristics	IHPP data is health checkup data for residents in the Iwaki district of Hirosaki City, Aomori Prefecture, Japan, and consists mainly of Japanese. The number of unique participants was 3,132, and 12,803 health checkup instances for 13 years (2005 to 2017) were used in this study. As the subject characteristics during first-time participation shown in Table 1, IHPP data consisted of 1,234 (39.4%) male and 1,898 (60.6%) female. The mean age of the study was 51.3 and the standard deviation was 16.0.
Recruitment	IHPP recruited the participants from residents in the Iwaki district of Hirosaki City, Aomori Prefecture, Japan. IHPP data is the health checkup data for the specific local inhabitant and may differ from the characteristics of ordinary Japanese people. Due to the nature of the health checkup data, the data are primarily comprised of healthy individuals rather than seriously ill individuals requiring hospitalization, which would not matter in verifying the effectiveness of our framework.
Ethics oversight	This study was approved by the Ethics Committee of Hirosaki University School of Medicine (approval number: 2019–009) and was conducted according to the recommendations of the Declaration of Helsinki. All participants provided written informed consent.

Note that full information on the approval of the study protocol must also be provided in the manuscript.
